# Hairy Conjugated Microporous Polymer Nanoparticles Facilitate Heterogeneous Photoredox Catalysis with Solvent-Specific Dispersibility

**DOI:** 10.1021/acsnano.2c07156

**Published:** 2022-10-12

**Authors:** Seunghyeon Kim, Katharina Landfester, Calum T. J. Ferguson

**Affiliations:** † Max Planck Institute for Polymer Research, Ackermannweg 10, 55128 Mainz, Germany; ‡ School of Chemistry, University of Birmingham, Edgbaston, Birmingham, B15 2TT, United Kingdom

**Keywords:** conjugated microporous polymer, hairy nanoparticles, dispersibility, heterogeneous photocatalysts, photoredox catalysis

## Abstract

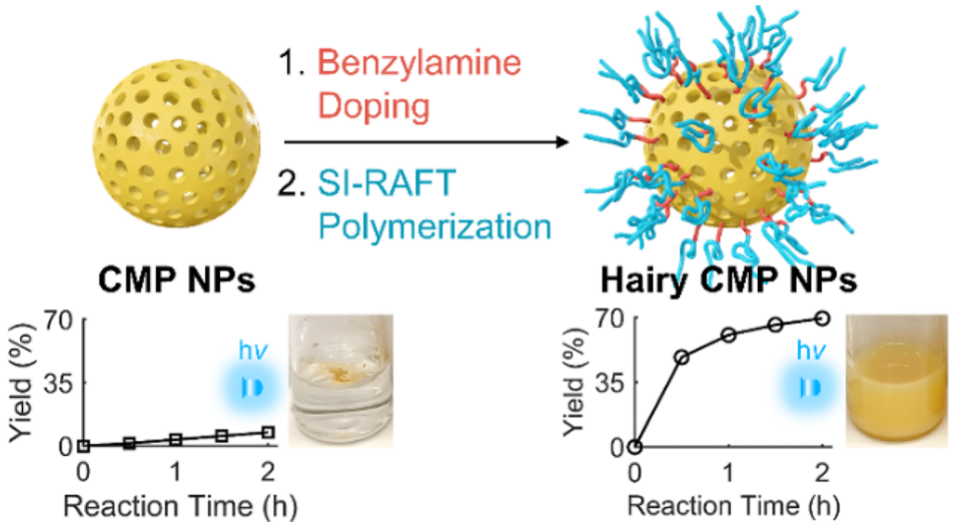

Substrate accessibility is a key limiting factor for the efficiency of heterogeneous photoredox catalysis. Recently, a high photoactive surface area of conjugated microporous polymer nanoparticles (CMP NPs) has made them promising candidates for overcoming the mass transfer limitation to achieve high photocatalytic efficiency. However, this potential has not been realized due to limited dispersibility of CMP NPs in many solvents, particularly in water. Here, we report a polymer grafting strategy that furnishes versatile hairy CMP NPs with enhanced solvent-specific dispersibility. The method associates hundreds of solvent-miscible repeating units with one chain end of the photocatalyst surface, allowing minimal modification to the CMP network that preserves its photocatalytic activity. Therefore, the enhanced dispersibility of hairy CMP NPs in organic solvents or aqueous solutions affords high efficiency in various photocatalytic organic transformations.

Visible light photoredox catalysis is a powerful tool in synthetic organic chemistry due to its unparalleled reactivity and greener synthetic routes, enabled by its facile access to radical species in a controlled and mild fashion.^1−8^ Various photocatalytic organic transformations have been developed using molecular photocatalysts such as transition metal complexes and organic dyes.^4−6^ Practically, though, these methods are limited by the toxicity and the high cost of rare metals, photocatalyst deactivation, and poor recyclability. To address these issues, organic heterogeneous photocatalysts, featuring low cost, high stability, and good recyclability, have been explored as alternatives for the wide use of organic photoredox catalysis in industrial applications.^9−13^

Conjugated microporous polymers (CMPs) have recently been demonstrated as versatile heterogeneous photocatalysts for organic transformations^11,14^ and solar fuel applications^15−17^ in that optical and electronic properties of the microporous semiconducting polymers can be easily tailored to individual applications. Moreover, the formation of CMP nanoparticles (NPs) by miniemulsion polymerization^18−20^ can greatly increase photoactive surface area-to-volume ratios. This large surface area can potentially improve substrate accessibility to the active sites on the photocatalysts^21,22^ as demonstrated by linear conjugated polymer nanoparticles with hydrophilic oligomer or polymer chains achieving good water dispersibility and considerable improvement in hydrogen evolution rates.^23−25^ However, this underexplored potentiality of the CMP NPs as efficient and broadly applicable photocatalysts has not been accessed owing to their limited dispersibility and colloidal stability in many solvents, particularly in water.

A handful of methods have been proposed to improve the dispersibility of bulk CMPs, but these methods could be too destructive to maintain original photocatalytic activity of CMP NPs. For instance, copolymerization of solvent-miscible monomers^26,27^ or ionizable ones, such as azulene,^28^ or postmodification of the alkyne-bearing CMPs with solvent-miscible functional groups via thiol–yne chemistry^29^ should introduce considerable amounts of defects to achieve sufficient dispersibility of CMP NPs. In addition to the devastating nature, incompatibility of hydrophilic monomers with oil-in-water miniemulsions and requirements of protonation of the azulene in acidic aqueous medium or alkyne groups in CMPs for the thiol–yne chemistry constrain the applicability of the methods. Ideally, a strategy for improving the dispersibility of CMP NPs should not only preserve their photocatalytic activity with minimal modification to the CMP network but also be compatible with their synthesis and downstream applications. Therefore, it is highly desirable to develop a mild and versatile strategy that would provide additional access to the photoactive sites of CMP NPs and enhance their photocatalytic efficiency in various applications.

Polymer-grafted NPs, also called hairy NPs, exhibit extraordinary dispersibility and colloidal stability in good solvents for the grafted polymers because of the favorable enthalpic interactions between the grafted polymer and the solvent and the steric repulsion between the hairy NPs.^30−32^ Besides, the polymer grafting can easily associate hundreds of solvent-miscible repeating units with one chain end anchored on the substrate surface,^33^ likely achieving better dispersibility with negligible modification to the CMP NPs.

Here, we present a polymer grafting strategy based on benzylamine doping of CMP NPs and surface-initiated reversible addition–fragmentation chain transfer (RAFT) polymerization. Varying the extent of benzylamine doping and the surface grafting density, the study reveals that the polymer grafting strategy can successfully enhance the solvent-specific dispersibility of CMP NPs without significantly altering their photocatalytic activity. The effects of the improved dispersibility and excessive benzylamine doping on the photocatalytic efficiency of CMP NPs are described using various photocatalytic organic transformations such as oxidative cycloaddition reactions and aza-Henry reaction in nitromethane. Finally, we demonstrate that the dispersibility of CMP NPs in aqueous solutions can be considerably enhanced by grafting water-soluble polymers, which drastically increases photocatalytic thiol–ene reaction kinetics and selectivity.

## Results and Discussion

**Figure 1:**
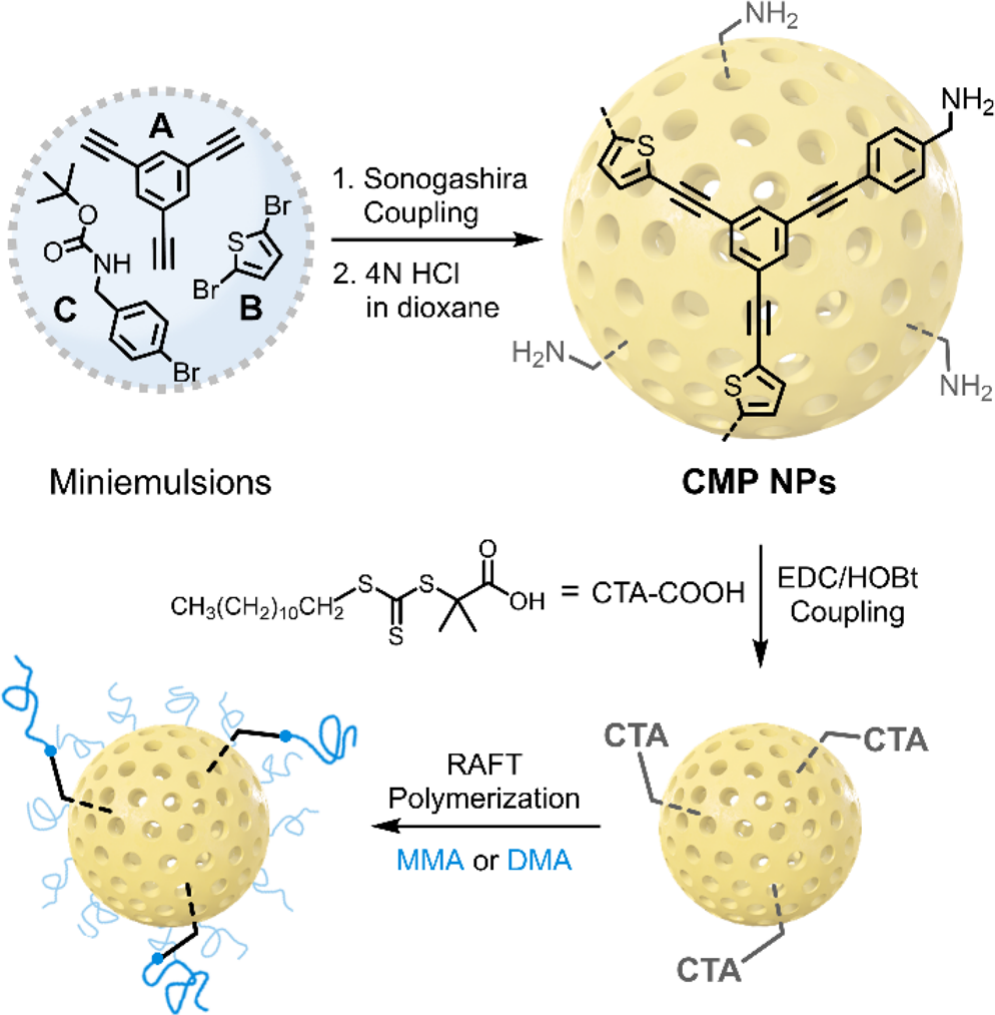
Synthetic route to conjugated microporous polymer nanoparticles (CMP NPs) and polymer grafting scheme. CTA: chain transfer agent, EDC: 1-ethyl-3-(3-(dimethylamino)propyl)carbodiimide, HOBt: 1-hydroxybenzotriazole, MMA: methyl methacrylate, DMA: *N*,*N*-dimethylacrylamide.

In this study, a cross-linked poly(*p*-phenylene ethynylene thiophene) network, which is made of 1,3,5-triethynylbenzene (**A**) and 2,5-bromothiophene (**B**), was selected as a pristine CMP backbone structure. To investigate impacts of benzylamine doping on the degree of polymerization and electronic structures of the CMP, its nanoparticle form and the benzylamine-doped CMP NPs were prepared in miniemulsions via Sonogashira cross-coupling of **A**, **B**, and *N*-Boc-4-bromobenzylamine (**C**) (Figure 1) with specific molar feed ratios of **B**:**C** = 100:0, 78:22, 60:40, and 45:55 considering the stoichiometry (Table S1). The resulting CMP NPs were denoted as CMP-*x* NPs with *x* = 0, 22, 40, and 55 depending on the molar fraction of **C**. Followed by *N*-Boc deprotection in 4 N HCl/dioxane, the primary amine groups on the CMP-*x* NPs were then employed to immobilize the chain transfer agent (CTA) by forming amide bonds to give CMP-*x*-CTA NPs. Finally, surface-initiated RAFT polymerization was conducted to graft poly(methyl methacrylate) (PMMA) or poly(*N*,*N*-dimethylacrylamide) (PDMA) chains from the surface of CMP NPs. The corresponding hairy CMP NPs were denoted as CMP-*x*-PMMA or CMP-*x*-PDMA NPs in this paper.

The prepared CMP-*x* NPs exhibited comparable Brunauer–Emmett–Teller (BET) surface areas of 326, 388, 392, and 337 m^2^ g^–1^ for CMP-0, CMP-22, CMP-40, and CMP-55, respectively (Table S2 and Figure S1). All of the CMP-*x* NPs showed microporosity, whose pore size distribution is centered at 1.1 and 1.3 nm (Figure S2). Interestingly, the surface area and volume of the micropores increase with the benzylamine content in CMP-*x* NPs (Table S3), which is presumably because the removal of Boc groups enlarged the micropores (Figure S2).^34^

Solid-state ^1^H magic-angle spinning (MAS) NMR spectra of CMP-*x* NPs (*x* = 22, 40, and 55) were employed to calculate the molar ratios of monomers **B** and **C** incorporated into the CMP network, which were **B**:**C** = 79.5:20.5, 69.8:30.2, and 65.8:34.2, respectively (Table S4). The molar fractions of **C** from the solid-state ^1^H NMR were smaller than those in the feed, and the differences were positively correlated with the amounts of **C** in the feed. This result indicates that more oligomers were formed when preparing CMP-40 and CMP-55 than CMP-22 and removed during purification. Solid-state ^13^C cross-polarization magic-angle spinning (CP-MAS) NMR spectra showed benzylic carbon signals at 67 ppm, aromatic carbon signals between 120 and 140 ppm, and triple-bond carbon signals at 83 and 93 ppm (Figure S3), supporting the proposed molecular structures of CMP-*x* NPs in Figure 1.

**Figure 2:**
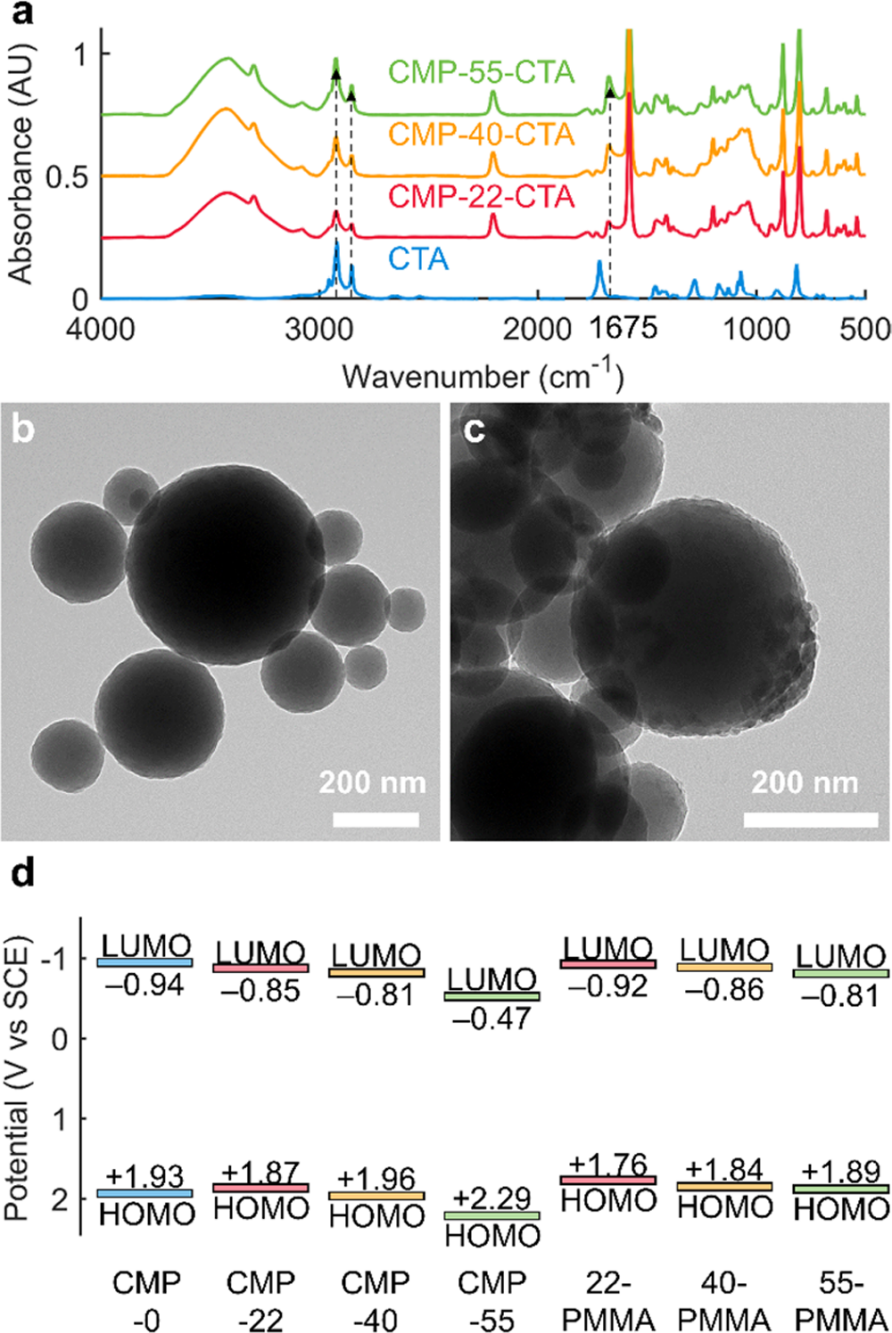
Characterization of CMP NPs. (a) FT-IR absorbance spectra of CTA-functionalized CMP NPs. TEM images of (b) CMP-55 and (c) CMP-55-PMMA. (d) Lowest unoccupied molecular orbital (LUMO) and highest occupied molecular orbital (HOMO) levels of CMP NPs estimated from their reduction potential and optical band gaps. SCE: saturated calomel electrode.

FT-IR spectra of CMP-*x* NPs (Figure S4) also featured characteristic absorptions by aromatic hydrocarbons (1583, 1400–1450, 1195, 876, and 800 cm^–1^) and alkyne (3300 and 2206 cm^–1^), complementing the solid-state NMR results. Furthermore, the FT-IR spectra demonstrated a positive correlation between the FT-IR signals of primary amine and the molar fractions of benzylamine groups in CMP-*x* NPs, which allowed us to expect the same trends in the CTA immobilization and polymer grafting. Gratifyingly, FT-IR spectroscopy of the CTA-immobilized CMP NPs revealed that amide C=O stretching (1675 cm^–1^) and alkane C–H stretching (2850–3000 cm^–1^) signals of immobilized CTA increased with the molar fractions of benzylamine groups as intended (Figure 2a), even though the same excess amount of CTA was used for all of the coupling reactions. Using a calibration curve with a CTA–benzylamine conjugate as a standard, we could estimate the number of the immobilized CTA (8.8 × 10^–8^ mol/mg CMP-22-CTA, 2.06 × 10^–7^ mol/mg CMP-40-CTA, and 2.58 × 10^–7^ mol/mg CMP-55-CTA) (Figure S5). The results indicate that not all of the primary amine ((1.3–3.3) × 10^–6^/mg CMP-*x* NPs) groups were accessible for the CTA coupling, explaining the origin of residual primary amine signals in the FT-IR spectra (Figure 2a).

TEM images confirmed that the CMP-*x* NPs obtained bumps on their surface upon polymer grafting (Figures 2b,c and S6). Assuming that free polymers and the surface-bound ones would exhibit similar molecular weight and dispersity,^32^ the gel permeation chromatography (GPC) results of the free polymers indicated that the grafted polymers—both PMMA and PDMA—would show unimodal molecular weight distributions (Figure S7), albeit relatively broad with the dispersity of 1.24 to 1.63 (Table S5). The reason might be that some propagating chains were terminated by irreversible processes occurring inside the pores of CMP NPs.^35,36^ Thermogravimetic analysis (TGA) results (Figure S8) indicated that more polymers were grafted from the CMP-*x*-CTA NPs with more surface-bound CTA (Figure S5). Indeed, the surface grafting density increased with the molar fractions of benzylamine groups (Table S5), verifying the hypothesis that the surface grafting density could be controlled by the extent of benzylamine doping in CMP-*x* NPs.

The absorption spectra of the CMP NPs were not significantly altered by the benzylamine doping and polymer grafting, and no clear connection between the extent of doping and the optical band gaps was observed: 2.87, 2.72, 2.77, and 2.76 eV for CMP-0, CMP-22, CMP-40, and CMP-55, respectively, and 2.68, 2.70, and 2.70 eV for CMP-22-PMMA, CMP-40-PMMA, and CMP-55-PMMA, respectively (Figure S9). The fluorescence emission spectra of polymer-grafted CMP NPs, on the other hand, revealed that excessive benzylamine doping and polymer grafting might change optical properties or polarity around the CMP NPs, leading to blue-shifted emission (Figure S10). Furthermore, the LUMO levels of the CMP-*x* NPs were lowered to more positive potential values versus saturated calomel electrode (SCE) as the benzylamine content increased (Figures 2d and S11). The origin of this shift in the LUMO could be explained by the replacement of electron-donating thiophene with the relatively more electron-withdrawing, hydrochloride salt of benzylamine, R–NH_3_^+^Cl^–^, lowering the molecular frontier orbitals of the conjugated polymers. Compared to CMP-*x* NPs, the LUMO levels of CMP-*x*-PMMA NPs were shifted to more negative potential values because the ammonium ion converted to electron-donating amide (Figures 2d and S12). The HOMO levels were calculated by adding the LUMO levels and optical band gaps because oxidation peaks were not clearly detected within the potential window of acetonitrile (−2.0 V to +2.0 V vs SCE) (Figures S11 and S12).

**Figure 3:**
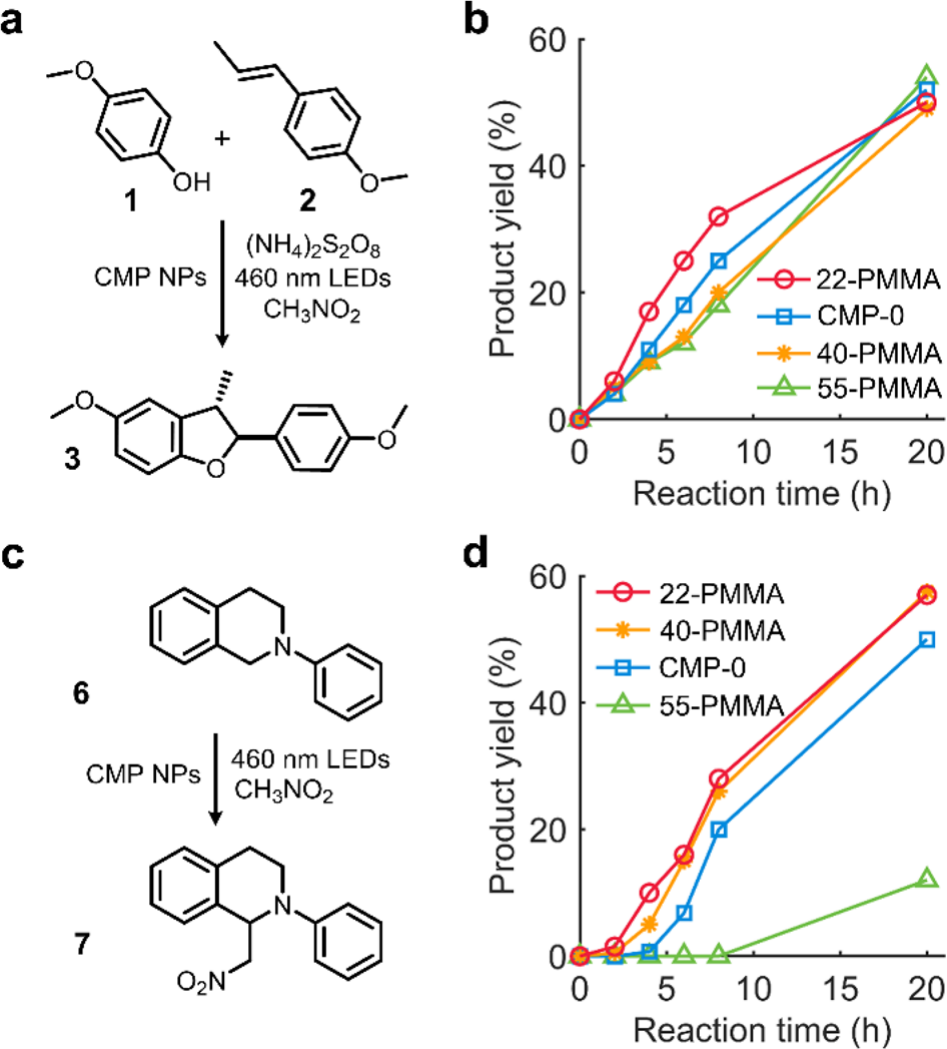
Photoredox catalysis of CMP-0 and PMMA-grafted CMP NPs in nitromethane. (a) Oxidative [3+2] cycloaddition of 4-methoxyphenol (**1**) to *trans*-anethole (**2**). (b) Kinetic profiles of the oxidative [3+2] cycloaddition reaction. Reaction rates (M s^–1^) were calculated using overall yields until 8 h: CMP-22-PMMA (5.56 × 10^7^), CMP-0 (4.34 × 10^7^), CMP-40-PMMA (3.47 × 10^7^), and CMP-55-PMMA (3.13 × 10^7^). (c) Photocatalyzed aza-Henry reaction of *N*-phenyltetrahydroisoquinoline (**6**). (d) Kinetic profiles of the aza-Henry reaction. Reaction rates (M s^–1^) were calculated using overall yields until 8 h: CMP-22-PMMA (4.86 × 10^7^), CMP-40-PMMA (4.51 × 10^7^), CMP-0 (3.47 × 10^7^), and CMP-55-PMMA (0.00 × 10^7^).

Given the optical band gaps in the blue light range (430–450 nm) and the deep HOMO levels (>+1.75 V vs SCE) of CMP NPs, photocatalytic oxidation reactions under visible light were selected for investigating the effects of the benzylamine content and polymer grafting density on photocatalytic activity of the CMP NPs. The reactions for CMP-*x*-PMMA NPs were developed in nitromethane to effectively demonstrate the impact of the grafted PMMA as a dispersant because nitromethane is a poor solvent for a CMP-0 NPs dispersion, but a good solvent for PMMA (Figures S13 and S14). First, we developed photocatalytic oxidative [3+2] cycloaddition^37,38^ of 4-methoxyphenol (**1**) to *trans*-anethole (**2**) under blue light (λ_max_ = 460 nm) (Figure 3a). With a photocatalytic system composed of 1 mg of CMP-0 in 1 mL of nitromethane, 0.05 mmol (1.0 equiv) of **1**, 1.5 equiv of **2**, and 2.0 equiv of (NH_4_)_2_S_2_O_8_ as a terminal oxidant, a high conversion (94.2%) and a moderate yield (50%) were obtained after 20 h under blue light (Table S6, entry 1). Control experiments demonstrated that the photocatalyst and light are all requisite components for the transformation (entries 2, 3). Increasing the amount of photocatalyst (2 mg/mL CMP-0) boosted the kinetics (entry 4, Figure S15), but the yield-to-conversion ratio (≈0.5) after 20 h was the same as the cases with reduced and increased light intensity (Table S6, entries 4–7). Notably, the CMP-*x* NPs showed similar kinetics to those of CMP-0 in nitromethane, indicating that the benzylamine doping did not affect the photocatalytic activity of CMP NPs in this reaction (Figure S16).

Under the optimized conditions, the photocatalytic efficiency of CMP-*x*-PMMA NPs and CMP-0 was examined via kinetic monitoring (Figure 3b). Interestingly, CMP-22-PMMA exhibited faster product formation than CMP-0 presumably due to its better dispersibility, but CMP-40-PMMA and CMP-55-PMMA showed slower rates than CMP-0, although the higher surface grafting density of CMP-55-PMMA allows it to form a better dispersion in nitromethane than CMP-22-PMMA (Figure S17). In fact, the kinetics with CMP-0 was faster than we expected, which was because CMP-0 could remain dispersed in nitromethane upon continuous stirring. Under intermittent stirring conditions, all CMP-*x*-PMMA NPs provided 15–50% faster kinetics than CMP-0 due to their enhanced dispersibility (Figure S18). Besides, higher surface grafting density (Table S5) and PMMA weight fractions (Figure S8) in CMP-40-PMMA and CMP-55-PMMA might contribute to hindering photocatalytic redox reactions at the surface of CMP NPs and reducing the photocatalyst loading. The similar enhancement in reaction kinetics by PMMA grafting was also observed in photocatalytic oxidative [2+2] cycloaddition of **2** to styrene (**4**) in nitromethane (see Figure S19 and Table S7 for the details of the reaction development) unless the grafting decreased the photocatalyst loading too much as in CMP-55-PMMA.

The use of (NH_4_)_2_S_2_O_8_ as the terminal oxidant in the above reactions was required for photocatalyst regeneration by removing the electrons from the LUMO. However, because of its high reduction potential (*E*_red_(S_2_O_8_^2–^|2SO_4_^2–^) = +2.01 V vs SHE and *E*_red_(S_2_O_8_^2–^| SO_4_^•–^+SO_4_^2–^) = +0.0 V vs SCE in HClO_4_ solutions),^39^ the LUMO level shift from −0.94 V to −0.81 V vs SCE by the benzylamine doping and polymer grafting did not make any noticeable difference in photocatalytic performance. To highlight the impact of the LUMO level shift, we sought to discover a photocatalytic redox-neutral reaction, where photocatalytic reduction of a substrate is affected by the LUMO potential of CMP NPs. According to the proposed mechanism of photocatalyzed aza-Henry reaction under anaerobic conditions (Figure S20),^40^ nitromethane acts as a terminal oxidant and the resulting CH_3_NO_2_^•–^ deprotonates the radical cation of *N*-phenyl-1,2,3,4-tetrahydroisoquinoline (**6**), initiating a radical pathway via an α-amino radical. Therefore, the reduction of nitromethane should occur at appreciable rates to activate the radical pathway. Considering the slightly more positive reduction onset potential of nitromethane solvent (*E*_red,onset_ = −0.67 V vs SCE) (Figure S21) than the LUMO level of CMP-55-PMMA (−0.81 V vs SCE), it was suggested that the nitromethane reduction with photoexcited CMP-*x*-PMMA NPs would be thermodynamically feasible. Nevertheless, we hypothesized that slower nitromethane reduction with CMP-55-PMMA than other CMP-*x*-PMMA NPs with more negative LUMO potential could limit the product formation in the photocatalyzed aza-Henry reaction of **6** (Figure 3c).

From a photocatalytic system composed of 1 mg of CMP-0 in 1 mL of nitromethane and 0.05 mmol (1.0 equiv) of **6**, a high conversion (100%) and a moderate yield (47%) were obtained after 20 h under blue light (11.9 mW cm^–2^) (Table S8, entry 1). Consistent with the proposed mechanism in the literature (Figure S20),^40^ control experiments disclosed not only the necessity of light but also background reactions without photocatalyst under intense blue light (entries 2, 3). Decreased light intensity (5.4 mW cm^–2^) enhanced the yield (55.5%) while suppressing the background reaction yield from 23% to 12% (entries 4, 5). Kinetic monitoring of the reaction (Figure 3d) exhibited that the PMMA grafting could enhance the photocatalytic efficiency likely due to the improvement in the dispersibility of CMP NPs (CMP-22-PMMA and CMP-40-PMMA), consistent with the previous results. The CMP-55-PMMA, on the other hand, showed only a 12% yield after 20 h of reaction, which was equal to the yield from the background reaction (without the photocatalyst) (entry 5). Moreover, the final conversion with CMP-55-PMMA was high (100%) (Figure S22) compared to that (29%) of the background reaction. This result supports the hypothesis that the formation of **7** could be hindered because of the slow nitromethane reduction with CMP-55-PMMA. From this study, it is concluded that excessive benzylamine doping can shift the LUMO to undesirable levels, reaffirming the need for mild modification to the CMP network when improving the dispersibility.

**Figure 4:**
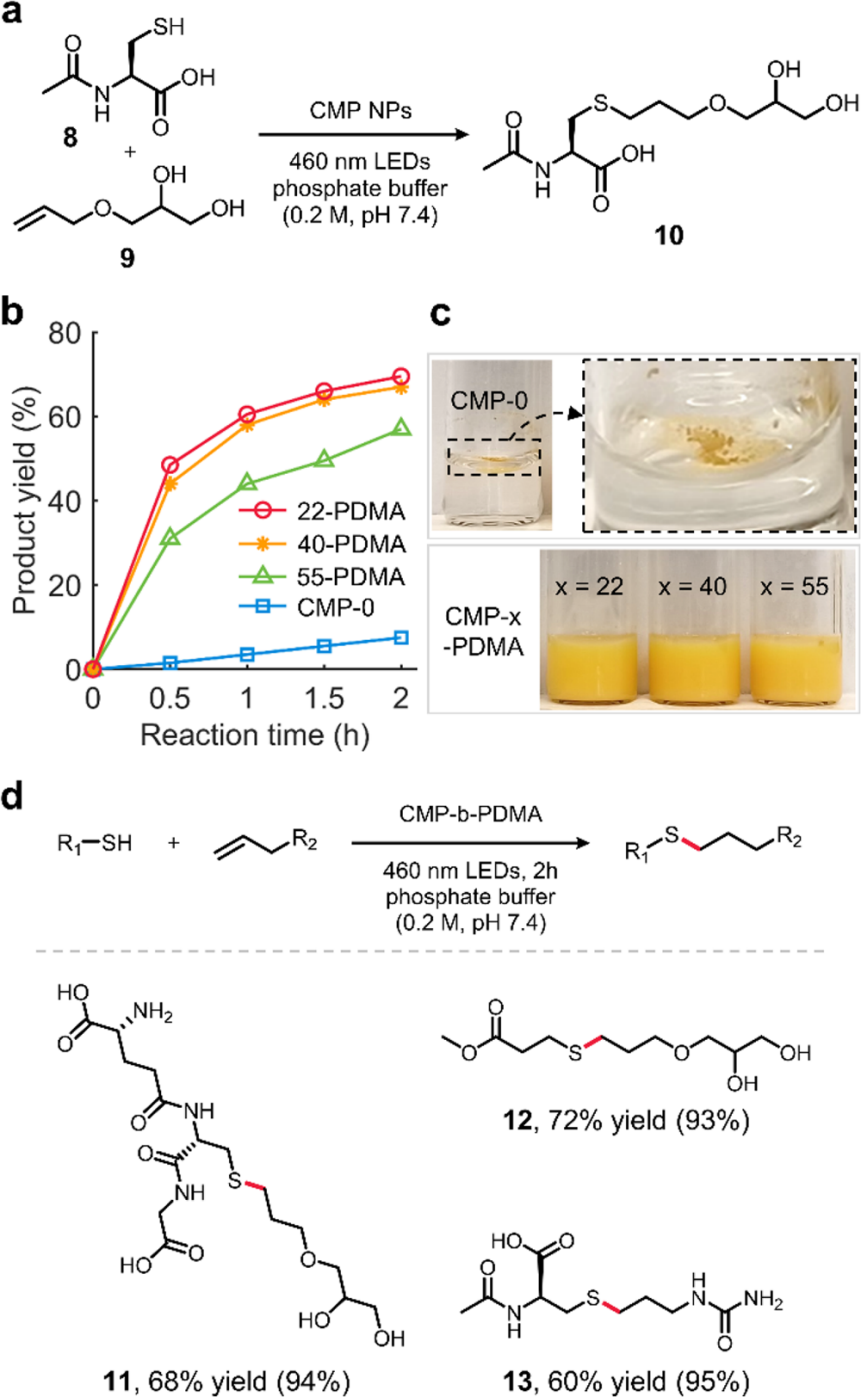
Photoredox catalysis of CMP-0 and PDMA-grafted CMP NPs in phosphate buffer solutions. (a) Photocatalytic thiol–ene reaction of *N*-acetyl-l-cysteine (**8**) and 3-allyloxy-1,2-propanediol (**9**). (b) Kinetic profiles of the thiol–ene reaction. Reaction rates (M s^–1^) were calculated using overall yields until 2 h: CMP-22-PDMA (9.65 × 10^6^), CMP-40-PDMA (9.31 × 10^6^), CMP-55-PDMA (7.92 × 10^6^), and CMP-0 (1.04 × 10^6^). (c) Image of CMP NPs in phosphate buffer solutions after 10 min of sonication. (d) Substrate scope of the thiol–ene reaction. Values in parentheses are selectivity.

The intrinsic hydrophobicity of CMP-0 has made it poorly dispersible in water, preventing its use for developing aqueous photoredox catalytic transformations. To overcome this limitation, we employed the polymer grafting strategy to decorate the surface of CMP NPs with water-soluble PDMA and examined the improvement in the dispersibility and photocatalytic efficiency of the resultant hairy CMP NPs. A photocatalytic radical thiol–ene reaction of *N*-acetyl-*L*-cysteine (**8**) and 3-allyloxy-1,2-propanediol (**9**) (Figure 4a) was selected as a model reaction in water for several reasons. First, the photocatalytic thiol–ene reaction can be used to modify cysteine-containing biopolymers through selective C–S bond formation at physiological pH.^41^ Second, the water-dispersible CMP NPs will not require organic cosolvents,^42^ which might affect the activity of biopolymers, for the bioconjugation in aqueous buffer solutions. Lastly, easy recovery of the heterogeneous photocatalysts should dramatically simplify purification steps and even enable recycling of the photocatalysts.

Due to the limited dispersibility of CMP-0, CMP-22, and CMP-40 in water (Figure S23), CMP-55 with the most benzylammonium groups (p*K*_a_ = 9.34)^43^ was used for developing the photocatalytic thiol–ene reaction. With a photocatalytic system composed of 1 mg of CMP-55 in 1 mL of phosphate buffer (pH 7.4, 0.2 M, and final pH of 6.3), 0.1 mmol (1.0 equiv) of **8**, and 2.0 equiv of **9**, a high conversion (82.5%) and a good yield (79%) were obtained after 2 h under blue light (Table S9, entry 1). Control experiments showed that both light and photocatalyst are required and an inert atmosphere is preferred for the reaction (entries 2–6). Kinetic profiles of the thiol–ene reaction (Figure 4b) demonstrated that the PDMA grafting substantially improved the photocatalytic efficiency of CMP NPs, which is attributed to their enhanced dispersibility in aqueous solutions using covalently bonded solvent-miscible polymer chains (Figure 4c). In fact, the key features of this polymer grafting strategy were not only the ability to enhance dispersibility of CMP NPs but also how to achieve it. To emphasize the importance of grafting polymer chains from CMP NPs in improving substrate accessibility to the photoactive sites, we compared the polymer grafting strategy with the use of surfactants such as sodium *n*-dodecyl sulfate (SDS). Given that SDS was used to prepare CMP NPs via oil-in-water miniemulsions, the use of SDS might be an easier way to disperse CMP NPs in aqueous media. Surprisingly, however, CMP-0 with SDS provided negligible enhancement in the thiol–ene reaction rate (1.53 × 10^6^ M s^–1^) compared to the case with PDMA grafting (9.65 × 10^6^ M s^–1^) (Figure S24). It is likely because the small-molecule surfactant significantly affected the substrate accessibility to the photoactive sites by occupying the interior pores of CMP NPs and surrounding the substrates via noncovalent interactions (Figure S24). The polymer grafting strategy, as opposed to the use of small-molecule surfactants, ensures high substrate accessibility with covalently bonded, long polymer chains because they do not block the micropores of CMP NPs and cannot be dissociated to form aggregates with substrates.

Notably, the dispersibility enhancement effect was still observed when much less benzylamine (2.5 and 5 mol %) was introduced into CMP NPs, although it could have been further improved with longer polymer chains (Figure S25). Short-chain grafting of PDMA, on the other hand, significantly affected dispersibility and thiol–ene reaction kinetics of CMP NPs, as demonstrated with CMP-22-PDMA with long chains (*M*_n_ = 6000 g/mol) and short chains (*M*_n_ = 1024 g/mol) (Figure S26). Compared to CMP-22, however, the short-chain-grafted CMP-22-PDMA still exhibited enhanced kinetics (Figure S27). Additionally, CMP-55 presented much faster kinetics than CMP-22 and CMP-40 (Figure S28), as expected from its better dispersibility (Figure S23), and showed even comparable kinetics to that of CMP-40-PDMA (Figure S29). This result implies that the dispersibility of CMP NPs in aqueous medium can be improved by just benzylamine doping, but the sufficient dispersibility can only be achieved with high benzylamine content that could lead to undesirable changes in electronic properties of the CMP network, as illustrated in the aza-Henry reaction with CMP-55-PMMA (Figure 3d). In contrast to CMP-*x* NPs, the thiol–ene reaction kinetics with PDMA-grafted CMP NPs followed opposite trends where CMP-*x*-PDMA with less benzylamine doping and lower surface grafting density yielded faster kinetics (Figure 4b). This tendency suggests that the benefits of enhanced dispersibility by additional PDMA chains in CMP-*x*-PDMA NPs (*x* > 22) (Figure S30) were outcompeted by negative impacts of the reduced photocatalyst loading per unit mass and hindered access of substrates to the active sites due to the densely grafted polymers (Figure S29).

Encouraged by the drastic improvement in the photocatalytic reaction kinetics and selectivity (Table S10) with the PDMA-grafted CMP NPs, we subsequently investigated the functional group tolerance of the optimized photocatalytic thiol–ene reaction protocol with the most efficient photocatalyst, CMP-22-PDMA (Figure 4d). The reactions of **9** and thiols with primary amine or ester groups smoothly proceeded in good yields and high selectivity (**11** and **12**). Allylurea and **8** could also be readily transformed into the desired product (**13**). Considering that the functional groups prevalent in biomolecules, such as alcohols, amines, amides, and carboxylic acids, remained intact after the reactions, the photocatalytic thiol–ene addition with water-dispersible hairy CMP NPs may prove useful for the selective bioconjugation. Finally, recyclability of the CMP-22-PDMA was examined with the photocatalytic thiol–ene reaction of **8** and **9** (Figure S31). The selectivity of the reaction remained higher than 90% and showed no tendency to decrease during five repeating cycles. The product yields after 2 h of the reaction, on the other hand, decreased from 69% to 54% over the five repeating cycles. However, given that the slightly reduced yields were mainly attributed to an inevitable weight loss (<5%) of the photocatalyst (1 mg) during 22 total times of centrifugation, we envision that the recyclability of CMP NPs would be improved further by grafting functional polymers to induce easier recovery of the photocatalysts upon external stimuli.^44^

## Conclusion

In summary, we have demonstrated a polymer grafting strategy to enhance solvent-specific dispersibility of conjugated microporous polymer nanoparticles for facilitating heterogeneous photoredox catalysis. The method produces hairy CMP NPs through doping of a CMP network with benzylamine and subsequent surface-initiated RAFT polymerization with solvent-compatible monomers, thus ensuring high dispersibility in any solvents. Scrutinizing various organic photocatalytic reactions with CMP NPs, we revealed that a minute change to the CMP network and controlled polymer grafting could improve their dispersibility and photocatalytic efficiency. By contrast, excessive benzylamine doping could change the redox potential to undesirable levels. These findings highlight the advantage of the polymer grafting strategy that can associate multiple solvent-miscible repeating units with a single modification site, which allows CMP NPs to preserve their activity. Furthermore, given its many different choices for CTAs and monomers, the polymer grafting strategy could bestow additional functions beyond solvent-specific dispersibility, contributing to development of CMP NPs for various applications.

## Methods

### Synthesis of CMP-0 and *N*-Boc-Protected CMP NPs

The cross-linked poly(*p*-phenylene ethynylene thiophene) CMP NPs (CMP-0) were prepared using previously described Sonogashira cross-coupling reactions in oil-in-water miniemulsions.^20^ First, 1,3,5-triethynylbenzene (monomer **A**, 50 mg), 2,5-dibromothiophene (monomer **B**, 125 mg), tetrakis(triphenylphosphine)palladium(0) (19.64 mg), and copper(I) iodide (3.24 mg) were dissolved in 2 mL of toluene in 40 mL vials containing stir bars. To the organic phase above were added an aqueous solution (17 mL) of sodium *n*-dodecyl sulfate (200 mg) and triethylamine (2 mL), and the mixture was vigorously stirred using a Branson SFX 550 digital sonifier operating at 70% amplitude for 2 min. The resultant miniemulsions were then purged with argon for 30 min and heated at 80 °C with continuous stirring overnight under an argon atmosphere. After cooling, each reaction mixture (∼20 mL) was mixed with 20 mL of ethanol and then transferred to centrifuge tubes (50 mL). The synthesized CMP NPs were separated from the unreacted monomers and surfactants by three rounds of centrifugation (centrifuge 5702 R, Eppendorf, 3000*g*, 10 min) with ethanol refill (20 mL). Finally, the precipitated CMP NPs were dried under high vacuum overnight.

The benzylamine-doped ones (CMP-22, -40, or -55) were prepared using the same protocol above except for monomer compositions. To the toluene solution (2 mL) including monomer **A** (50 mg), tetrakis(triphenylphosphine)palladium(0) (19.64 mg), and copper(I) iodide (3.24 mg), decreasing amounts of monomer **B** and increasing amounts of *N*-Boc-4-bromobenzylamine (monomer **C**) were added for preparing each CMP-*x* NP as follows: CMP-22 (**B**: 110.1 mg; **C**: 37.2 mg), CMP-40 (**B**: 94.37 mg; **C**: 74.42 mg), and CMP-55 (**B**: 78.65 mg; **C**: 111.63 mg).

### Deprotection of *N*-Boc-Protected CMP NPs and Solvolysis of Residual Sodium *n*-Dodecyl Sulfate

To cleave the Boc group and further remove the residual surfactant, the dried CMP NPs above were dispersed in 4 N HCl in 1,4-dioxane^45^ at 5 mg/mL concentrations using an ultrasonic bath sonicator (USC300TH, VWR) for 10 min at 25 °C. After stirring the mixture overnight at room temperature, the CMP NPs were separated using centrifugation (3000*g*, 10 min) with ethanol refill (20 mL for 100 mg of CMP NPs) three times. The precipitated CMP-*x* NPs (*x* = 0, 22, 40, and 55) were dried under high vacuum overnight. Despite the lack of *N*-Boc groups, CMP-0 was also subject to the same reaction conditions to solvolyze residual sodium *n*-dodecyl sulfate^46^ and facilitate its removal.

### Functionalization of CMP NPs with Chain Transfer Agent

The CTA-functionalized CMP NPs were prepared by forming amide bonds via EDC/HOBt coupling.^47^ To a 40 mL vial with a stir bar, 70 mg of the dried CMP-55 NPs including maximum 24.5 mg of benzylamine (228.7 μmol, 0.2 equiv, estimated without excluding oligomers from CMP NPs) and 10.5 mL of extra dry *N*,*N*-dimethylformamide (DMF) were added. The reaction mixture was sonicated for 10 min using the bath sonicator and then cooled at 0 °C. To the reaction mixture were added 420 mg (1.0 equiv) of 2-[[(dodecylsulfanyl)carbonothioyl]sulfanyl]-2-methylpropanoic acid (CTA) and 185.6 mg (1.2 equiv, anhydrous basis) of 1-hydroxybenzotriazole (HOBt). After 10 min, *N*,*N*-diisopropylethylamine (0.498 mL, 2.5 equiv) was added to the mixture. Finally, 15 min later, 263 mg (1.2 equiv) of 1-ethyl-3-(3-(dimethylamino)propyl)carbodiimide hydrochloride (EDC·HCl) in 2.1 mL of DMF was added to the reaction mixture above at 0 °C dropwise. After stirring the mixture for 12 h at room temperature under a nitrogen atmosphere, the CMP-55-CTA NPs were separated using centrifugation (3000*g*, 10 min) with ethanol refill (15 mL) three times. The precipitated CMP-55-CTA NPs were dried under high vacuum overnight. The same procedure applied to other CMP-*x* NPs (*x* = 22 and 40) without changing the amount of reagents.

### PMMA Grafting from CTA-Immobilized CMP NPs

The surface-initiated RAFT polymerization of methyl methacrylate (MMA) was carried out with thermal initiation. To a 40 mL vial equipped with a stir bar were added 20 mg of CMP-*x*-CTA NPs (*x* = 22, 40, or 55) and 10 mL of DMF, and the mixture was sonicated for 10 min using the bath sonicator. Monomer solution (10 mL) was prepared by dissolving 221.8 mg (608 μmol, 1 equiv) of the free CTA, 6.08 g (60.7 mmol, 100 equiv) of MMA, and 20 mg (122 μmol, 0.2 equiv) of azobis(isobutyronitrile) (AIBN) in 3.2 mL of DMF and then added to the CMP NP dispersion. The system was purged with nitrogen for 30 min, sealed, and connected to a nitrogen-filled balloon. The polymerization was conducted under a nitrogen atmosphere at 70 °C for 16 h and quenched by cooling the vial in an ice bath (0 °C). An aliquot of the solution was taken out for ^1^H NMR measurement to estimate the monomer conversion. The rest of the reaction mixture was diluted by 20 mL of DMF and centrifuged to collect PMMA-grafted CMP NPs. To minimize the loss of the hairy CMP NPs, the separation was carried out by four rounds of centrifugation (HERAEUS FRESCO 21 centrifuge, Thermo Scientific) at 21100*g* for 10 min. The first supernatant was used for GPC analysis to determine molecular weight and its distribution of the free polymers. For the first three cycles of the separation, DMF was used to redisperse the hairy CMP NPs, but for the final round, ethanol was used. The final pellet was dried under high vacuum overnight. To estimate the weight fraction of the grafted polymer, 10 mg of the CMP-*x*-PMMA (*x* = 22, 40, or 55) was subject to TGA.

### PDMA Grafting from CTA-Immobilized CMP NPs

The surface-initiated RAFT polymerization of *N*,*N*-dimethylacrylamide (DMA) was carried out with thermal initiation. To a 40 mL vial equipped with a stir bar were added 20 mg of CMP-*x*-CTA NPs (*x* = 22, 40, or 55) and 10 mL of 50 v/v % DMF in deionized water, and the mixture was sonicated for 10 min using the bath sonicator. Monomer solution (10 mL) was prepared by mixing 345 mg (946 μmol, 1 equiv) of the free CTA, 9.75 mL (94.6 mmol, 100 equiv) of MMA, and 31.2 mg (190 μmol, 0.2 equiv) of AIBN and then added to the CMP NP dispersion. The system was purged with nitrogen for 30 min, sealed, and connected to a nitrogen-filled balloon. The polymerization was conducted under a nitrogen atmosphere at 70 °C for 16 h and quenched by cooling the vial in an ice bath (0 °C). An aliquot of the solution was taken out for ^1^H NMR measurement to estimate the monomer conversion. The rest of the reaction mixture was diluted by 20 mL of ethanol and centrifuged to collect PDMA-grafted CMP NPs. To minimize the loss of the hairy CMP NPs, the separation was carried out by four rounds of centrifugation at 21100*g* for 10 min with ethanol refill. The first supernatant was used for GPC analysis to determine molecular weight and its distribution of the free polymers. The final pellet was dried under high vacuum overnight. To estimate the weight fraction of the grafted polymer, 10 mg of the CMP-*x*-PDMA (*x* = 22, 40, or 55) was subject to TGA.

### Surface Grafting Density Calculation

The surface grafting density (σ) of PMMA- or PDMA-grafted CMP NPs was estimated using the established method in the literature.^32^ According to eq 1, the average number of grafted polymer chains per nanoparticle was calculated by *n*_polymer_*N*_A_, where *n*_polymer_ is the average mole number of polymer chains per single particle and *N*_A_ is Avogadro’s number. According to eq 2, *n*_polymer_ could be calculated using a weight fraction of the grafted polymer (*w*_p_) from the TGA data (Figure S7), *M*_n_ of the grafted polymers from GPC data (Table S5), density (ρ) of the CMP NPs (Figure S10), and average radii (*r*) of CMP NPs from TEM images (Figure S7). Finally, the *n*_polymer_*N*_A_ values were divided by the average surface area (*S* = 4π*r*^2^) of CMP NPs to obtain the surface grafting density (σ). The surface density (σ) of CTA in CMP-*x*-CTA NPs was calculated by substituting *n*_polymer_ with *n*_CTA_, where *n*_CTA_ was obtained using the FT-IR calibration curve for the CTA quantification (Figure S5) instead of TGA.
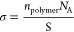
1
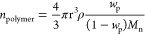
2

## Supporting Information Available

General information for materials and characterization techniques; photoredox catalytic reactions with CMP NPs; microporous, optical, and electrochemical properties of CMP NPs; TEM images of hairy CMP NPs; data for surface grafting density calculations; dispersibility and colloidal stability of CMP-0 NPs in various organic solvents; particle size distribution of hairy CMP NPs; development of oxidative [2+2] cycloaddition of *trans*-anethole to styrene; optimization tables for the photoredox catalytic reactions; kinetic profiles of photocatalytic thiol–ene reaction with various CMP NPs; recyclability tests with CMP-22-PDMA (PDF)

## References

[ref1] Yoon T. P.; Ischay M. A.; Du J. Visible Light Photocatalysis as a Greener Approach to Photochemical Synthesis. Nat. Chem. 2010, 2, 527–532. 10.1038/nchem.687. 20571569

[ref2] Narayanam J. M. R.; Stephenson C. R. J. Visible Light Photoredox Catalysis: Applications in Organic Synthesis. Chem. Soc. Rev. 2011, 40, 102–113. 10.1039/B913880N. 20532341

[ref3] Prier C. K.; Rankic D. A.; MacMillan D. W. C. Visible Light Photoredox Catalysis with Transition Metal Complexes: Applications in Organic Synthesis. Chem. Rev. 2013, 113, 5322–5363. 10.1021/cr300503r. 23509883 PMC4028850

[ref4] Shaw M. H.; Twilton J.; MacMillan D. W. C. Photoredox Catalysis in Organic Chemistry. J. Org. Chem. 2016, 81, 6898–6926. 10.1021/acs.joc.6b01449. 27477076 PMC4994065

[ref5] Romero N. A.; Nicewicz D. A. Organic Photoredox Catalysis. Chem. Rev. 2016, 116, 10075–10166. 10.1021/acs.chemrev.6b00057. 27285582

[ref6] Twilton J.; Le C.; Zhang P.; Shaw M. H.; Evans R. W.; MacMillan D. W. C. The Merger of Transition Metal and Photocatalysis. Nat. Rev. Chem. 2017, 1, 0052 10.1038/s41570-017-0052.

[ref7] McAtee R. C.; McClain E. J.; Stephenson C. R. J. Illuminating Photoredox Catalysis. Trends Chem. 2019, 1, 111–125. 10.1016/j.trechm.2019.01.008. PMC9608853 36313819

[ref8] Crisenza G. E. M.; Melchiorre P. Chemistry Glows Green with Photoredox Catalysis. Nat. Commun. 2020, 11, 803 10.1038/s41467-019-13887-8. 32029742 PMC7005190

[ref9] Wong Y. L.; Tobin J. M.; Xu Z.; Vilela F. Conjugated Porous Polymers for Photocatalytic Applications. J. Mater. Chem. A 2016, 4, 18677–18686. 10.1039/C6TA07697A.

[ref10] Savateev A.; Ghosh I.; König B.; Antonietti M. Photoredox Catalytic Organic Transformations Using Heterogeneous Carbon Nitrides. Angew. Chem., Int. Ed. 2018, 57, 15936–15947. 10.1002/anie.201802472. 30066478

[ref11] Li R.; Byun J.; Huang W.; Ayed C.; Wang L.; Zhang K. A. I. Poly(Benzothiadiazoles) and Their Derivatives as Heterogeneous Photocatalysts for Visible-Light-Driven Chemical Transformations. ACS Catal. 2018, 8, 4735–4750. 10.1021/acscatal.8b00407.

[ref12] Xiao J.; Liu X.; Pan L.; Shi C.; Zhang X.; Zou J. J. Heterogeneous Photocatalytic Organic Transformation Reactions Using Conjugated Polymers-Based Materials. ACS Catal. 2020, 10, 12256–12283. 10.1021/acscatal.0c03480.

[ref13] Liu R. Y.; Guo S.; Luo S.-X. L.; Swager T. M. Solution-Processable Microporous Polymer Platform for Heterogenization of Diverse Photoredox Catalysts. Nat. Commun. 2022, 13, 2775 10.1038/s41467-022-29811-6. 35624102 PMC9142596

[ref14] Lee J.-S. M.; Cooper A. I. Advances in Conjugated Microporous Polymers. Chem. Rev. 2020, 120, 2171–2214. 10.1021/acs.chemrev.9b00399. 31990527 PMC7145355

[ref15] Sprick R. S.; Jiang J.-X.; Bonillo B.; Ren S.; Ratvijitvech T.; Guiglion P.; Zwijnenburg M. A.; Adams D. J.; Cooper A. I. Tunable Organic Photocatalysts for Visible-Light-Driven Hydrogen Evolution. J. Am. Chem. Soc. 2015, 137, 3265–3270. 10.1021/ja511552k. 25643993

[ref16] Vyas V. S.; Haase F.; Stegbauer L.; Savasci G.; Podjaski F.; Ochsenfeld C.; Lotsch B. V. A Tunable Azine Covalent Organic Framework Platform for Visible Light-Induced Hydrogen Generation. Nat. Commun. 2015, 6, 8508 10.1038/ncomms9508. 26419805 PMC4598847

[ref17] Wang L.; Wan Y.; Ding Y.; Wu S.; Zhang Y.; Zhang X.; Zhang G.; Xiong Y.; Wu X.; Yang J.; Xu H. Conjugated Microporous Polymer Nanosheets for Overall Water Splitting Using Visible Light. Adv. Mater. 2017, 29, 1–8. 10.1002/adma.201702428 28833545

[ref18] Zhang P.; Weng Z.; Guo J.; Wang C. Solution-Dispersible, Colloidal, Conjugated Porous Polymer Networks with Entrapped Palladium Nanocrystals for Heterogeneous Catalysis of the Suzuki-Miyaura Coupling Reaction. Chem. Mater. 2011, 23, 5243–5249. 10.1021/cm202283z.

[ref19] Ma B. C.; Ghasimi S.; Landfester K.; Vilela F.; Zhang K. A. I. Conjugated Microporous Polymer Nanoparticles with Enhanced Dispersibility and Water Compatibility for Photocatalytic Applications. J. Mater. Chem. A 2015, 3, 16064–16071. 10.1039/C5TA03820K.

[ref20] Ma B. C.; Ghasimi S.; Landfester K.; Zhang K. A. I. Enhanced Visible Light Promoted Antibacterial Efficiency of Conjugated Microporous Polymer Nanoparticles via Molecular Doping. J. Mater. Chem. B 2016, 4, 5112–5118. 10.1039/C6TB00943C. 32263508

[ref21] Bloh J. Z. Intensification of Heterogeneous Photocatalytic Reactions Without Efficiency Losses: The Importance of Surface Catalysis. Catal. Lett. 2021, 151, 3105–3113. 10.1007/s10562-021-03573-0.

[ref22] Heuer J.; Ferguson C. T. J. Photocatalytic Polymer Nanomaterials for the Production of High Value Compounds. Nanoscale 2022, 14, 1646–1652. 10.1039/D1NR06985C. 35037676

[ref23] Wang L.; Fernández-Terán R.; Zhang L.; Fernandes D. L. A.; Tian L.; Chen H.; Tian H. Organic Polymer Dots as Photocatalysts for Visible Light-Driven Hydrogen Generation. Angew. Chem., Int. Ed. 2016, 55, 12306–12310. 10.1002/anie.201607018. 27604393

[ref24] Hu Z.; Wang Z.; Zhang X.; Tang H.; Liu X.; Huang F.; Cao Y. Conjugated Polymers with Oligoethylene Glycol Side Chains for Improved Photocatalytic Hydrogen Evolution. iScience 2019, 13, 33–42. 10.1016/j.isci.2019.02.007. 30818223 PMC6393733

[ref25] Kosco J.; Gonzalez-Carrero S.; Howells C. T.; Zhang W.; Moser M.; Sheelamanthula R.; Zhao L.; Willner B.; Hidalgo T. C.; Faber H.; Purushothaman B.; Sachs M.; Cha H.; Sougrat R.; Anthopoulos T. D.; Inal S.; Durrant J. R.; McCulloch I. Oligoethylene Glycol Side Chains Increase Charge Generation in Organic Semiconductor Nanoparticles for Enhanced Photocatalytic Hydrogen Evolution. Adv. Mater. 2022, 34, 1–9. 10.1002/adma.202105007. 34714562

[ref26] Dawson R.; Laybourn A.; Clowes R.; Khimyak Y. Z.; Adams D. J.; Cooper A. I. Functionalized Conjugated Microporous Polymers. Macromolecules 2009, 42, 8809–8816. 10.1021/ma901801s.

[ref27] Cheng G.; Hasell T.; Trewin A.; Adams D. J.; Cooper A. I. Soluble Conjugated Microporous Polymers. Angew. Chem., Int. Ed. 2012, 51, 12727–12731. 10.1002/anie.201205521. 23143745

[ref28] Ghasimi S.; Landfester K.; Zhang K. A. I. Water Compatible Conjugated Microporous Polyazulene Networks as Visible-Light Photocatalysts in Aqueous Medium. ChemCatChem. 2016, 8, 694–698. 10.1002/cctc.201501102.

[ref29] Urakami H.; Zhang K.; Vilela F. Modification of Conjugated Microporous Poly-Benzothiadiazole for Photosensitized Singlet Oxygen Generation in Water. Chem. Commun. 2013, 49, 2353–2355. 10.1039/c3cc38956a. 23407715

[ref30] Mai W.; Sun B.; Chen L.; Xu F.; Liu H.; Liang Y.; Fu R.; Wu D.; Matyjaszewski K. Water-Dispersible, Responsive, and Carbonizable Hairy Microporous Polymeric Nanospheres. J. Am. Chem. Soc. 2015, 137, 13256–13259. 10.1021/jacs.5b08978. 26426860

[ref31] Wright R. A. E.; Wang K.; Qu J.; Zhao B. Oil-Soluble Polymer Brush Grafted Nanoparticles as Effective Lubricant Additives for Friction and Wear Reduction. Angew. Chem., Int. Ed. 2016, 55, 8656–8660. 10.1002/anie.201603663. 27265613

[ref32] Chancellor A. J.; Seymour B. T.; Zhao B. Characterizing Polymer-Grafted Nanoparticles: From Basic Defining Parameters to Behavior in Solvents and Self-Assembled Structures. Anal. Chem. 2019, 91, 6391–6402. 10.1021/acs.analchem.9b00707. 31013073

[ref33] Zoppe J. O.; Ataman N. C.; Mocny P.; Wang J.; Moraes J.; Klok H.-A. Surface-Initiated Controlled Radical Polymerization: State-of-the-Art, Opportunities, and Challenges in Surface and Interface Engineering with Polymer Brushes. Chem. Rev. 2017, 117, 1105–1318. 10.1021/acs.chemrev.6b00314. 28135076

[ref34] Wang Z. J.; Ghasimi S.; Landfester K.; Zhang K. A. I. Highly Porous Conjugated Polymers for Selective Oxidation of Organic Sulfides under Visible Light. Chem. Commun. 2014, 50, 8177–8180. 10.1039/C4CC02861A. 24927479

[ref35] Pasetto P.; Blas H.; Audouin F.; Boissière C.; Sanchez C.; Save M.; Charleux B. Mechanistic Insight into Surface-Initiated Polymerization of Methyl Methacrylate and Styrene via ATRP from Ordered Mesoporous Silica Particles. Macromolecules 2009, 42, 5983–5995. 10.1021/ma9003506.

[ref36] Liu H.; Zhu Y.-L.; Zhang J.; Lu Z.-Y.; Sun Z.-Y. Influence of Grafting Surface Curvature on Chain Polydispersity and Molecular Weight in Concave Surface-Initiated Polymerization. ACS Macro Lett. 2012, 1, 1249–1253. 10.1021/mz3003374. 35607149

[ref37] Blum T. R.; Zhu Y.; Nordeen S. A.; Yoon T. P. Photocatalytic Synthesis of Dihydrobenzofurans by Oxidative [3 + 2] Cycloaddition of Phenols. Angew. Chem., Int. Ed. 2014, 53, 11056–11059. 10.1002/anie.201406393. PMC4220618 25155300

[ref38] Huang W.; Huber N.; Jiang S.; Landfester K.; Zhang K. A. I. Covalent Triazine Framework Nanoparticles via Size-Controllable Confinement Synthesis for Enhanced Visible-Light Photoredox Catalysis. Angew. Chem., Int. Ed. 2020, 59, 18368–18373. 10.1002/anie.202007358. PMC7590189 32697384

[ref39] Shafiee S. A.; Aarons J.; Hamzah H. H. Review—Electroreduction of Peroxodisulfate: A Review of a Complicated Reaction. J. Electrochem. Soc. 2018, 165, H785–H798. 10.1149/2.1161811jes.

[ref40] Bartling H.; Eisenhofer A.; König B.; Gschwind R. M. The Photocatalyzed Aza-Henry Reaction of N-Aryltetrahydroisoquinolines: Comprehensive Mechanism, H^•^- versus H^+^-Abstraction, and Background Reactions. J. Am. Chem. Soc. 2016, 138, 11860–11871. 10.1021/jacs.6b06658. 27541322

[ref41] Choi H.; Kim M.; Jang J.; Hong S. Visible-Light-Induced Cysteine-Specific Bioconjugation: Biocompatible Thiol-Ene Click Chemistry. Angew. Chemie Int. Ed. 2020, 59, 22514–22522. 10.1002/anie.202010217. 32864829

[ref42] Tyson E. L.; Niemeyer Z. L.; Yoon T. P. Redox Mediators in Visible Light Photocatalysis: Photocatalytic Radical Thiol-Ene Additions. J. Org. Chem. 2014, 79, 1427–1436. 10.1021/jo500031g. 24428433 PMC3985841

[ref43] Hall H. K. Correlation of the Base Strengths of Amines. J. Am. Chem. Soc. 1957, 79, 5441–5444. 10.1021/ja01577a030.

[ref44] Ferguson C. T. J.; Huber N.; Landfester K.; Zhang K. A. I. Dual-Responsive Photocatalytic Polymer Nanogels. Angew. Chem., Int. Ed. 2019, 58, 10567–10571. 10.1002/anie.201903309. 31066484

[ref45] Han G.; Tamaki M.; Hruby V. J. Fast, Effcient and Selective Deprotection of the Tert-Butoxycarbonyl (Boc) Group Using HCl/Dioxane (4 M). J. Pept. Res. 2001, 58, 338–341. 10.1034/j.1399-3011.2001.00935.x. 11606219

[ref46] Burstein S.; Lieberman S. Kinetics and Mechanism of Solvolysis of Steroid Hydrogen Sulfates. J. Am. Chem. Soc. 1958, 80, 5235–5239. 10.1021/ja01552a054.

[ref47] Dunetz J. R.; Magano J.; Weisenburger G. A. Large-Scale Applications of Amide Coupling Reagents for the Synthesis of Pharmaceuticals. Org. Process Res. Dev. 2016, 20, 140–177. 10.1021/op500305s.

