# Supplementary material for: Hairy Conjugated Microporous Polymer Nanoparticles Facilitate Heterogeneous Photoredox Catalysis with Solvent-Specific Dispersibility
Source: ACS Nano. 2022 Oct 12;16(10):17041–8. doi: 10.1021/acsnano.2c07156 (PMC9620398; doi:10.1021/acsnano.2c07156)
Supplement: Supplementary file 1 — nn2c07156_si_001.pdf [file nn2c07156_si_001.pdf]

Supporting Information

Hairy conjugated microporous polymer nanoparticles facilitate heterogeneous photoredox catalysis with solvent-specific dispersibility

Seunghyeon Kim[a], Katharina Landfester[a], and Calum T. J. Ferguson[a,b*]

[a]Max Planck Institute for Polymer Research, Ackermannweg 10, 55128 Mainz, Germany

[b]School of Chemistry, University of Birmingham, Edgbaston, Birmingham, B15 2TT, United Kingdom

*Corresponding Author:

Dr. Calum T. J. Ferguson

E-mail: ferguson@mpip-mainz.mpg.de

Table of Contents

Ⅰ. General Information for Materials and Characterization Techniques .... S−2

Ⅱ. Photoredox Catalytic Reactions with CMP NPs .... S−4

Ⅲ. Supplementary Figures .... S−11

Ⅳ. Supplementary Tables .... S−37

Ⅴ. References .... S−45

Appendix: ^{1}H and ^{13}C NMR spectra .... S−47

Ⅰ. General Information for Materials and Characterization Techniques

Unless otherwise noted, all chemicals and solvents were purchased from chemical suppliers (Sigma-Aldrich, Acros, TCI Deutschland, Oakwood Chemical, or VWR Chemicals) and used without further purification. Hydrogen chloride (4N in 1,4-dioxane) and *N*,*N*-dimethylformamide (DMF, extra dry) were purchased from Acros in AcroSeal™ bottles. *N*-Boc-4-bromobenzylamine was purchased from ChemScene LLC. *N*-Phenyltetrahydroisoquinoline was prepared according to referenced literature procedure. Methyl methacrylate (MMA) and *N*,*N*-dimethylacrylamide (DMA) were purified by eluting through neutral, Brockmann I aluminum oxide (50–200 µm, 60Å). Products were purified by column chromatography using MACHEREY-NAGEL silica gel (0.04–0.063 mm, 60Å) or cytiva Sephadex™ LH-20 resin.

Brunauer-Emmett-Teller (BET) measurements: BET surface areas of CMP NPs were measured with nitrogen sorption at 77 K using a Micromeritics Tristar Ⅱ Plus with samples degassed for 12 hours at 120 °C under vacuum prior to analysis. Pore size distributions were estimated using the Non-Local Density Functional Theory (NLDFT) model for carbon slit pore geometry built in Tristar Ⅱ Plus.

Nuclear magnetic resonance (NMR): ^{1}H and ^{13}C NMR spectra for purified compounds were collected using a 400 MHz Bruker Avance NEO NMR spectrometer. ^{1}H NMR spectra for crude products were collected using a 700 MHz Bruker Avance Ⅲ NMR spectrometer. Solid-state ^{1}H MAS and ^{13}C CP-MAS NMR measurements were performed using a Bruker Avance Ⅲ solid-state NMR spectrometer operating at 700 MHz ^{1}H Larmor frequency with 25 kHz MAS, 100 kHz rf, and 3 ms CP contact. Broadband ^{1}H decoupling was used during the ^{13}C NMR measurements. All NMR spectra were processed with MestReNova software. ^{1}H NMR and ^{13}C NMR chemical shifts were calibrated using residual solvent as an internal reference ($CHCl_3$: δ 7.26 ppm in ^{1}H NMR and δ 77.16 ppm in ^{13}C NMR; H_2O: δ 4.70 ppm in ^{1}H NMR) or $SiMe_4$ (δ 0.0 ppm in ^{13}C NMR). Multiplicities are described as follows: s = singlet, d = doublet, t = triplet, q = quartet, dd = doublet of doublets, td = triplet of doublets, dt = doublet of triplets, tt = triplet of triplets, and m = multiplet.

Mass spectrometry (MS): ESI mass spectra were obtained using Shimadzu GC-2010 plus gas chromatography and QP2010 ultra mass spectrometer. APCI mass spectra were obtained using an Advion expression® Compact Mass Spectrometer. High-resolution ESI mass spectra were obtained using Waters SYNAPT G2-Si spectrometer.

Fourier-transform infrared (FT-IR) spectroscopy: All IR spectra were obtained on a Bruker VERTEX 70 FT-IR spectrometer and processed with OPUS software to obtain baseline-corrected absorbance spectra.

The KBr pellets were prepared by mixing 2 mg of samples with 360 mg of finely powdered potassium bromide immediately before each measurement.

Transmission electron microscopy (TEM): The morphologies of CMP NPs were recorded using JEOL JEM-1400.

Gel permeation chromatography (GPC): Molecular weight distributions (M_n, M_w, and $Đ$) of free polymers were analyzed using an Agilent 1260 Infinity SECcuirty GPC System equipped with PSS SECcurity UV 270 nm and refractive index detectors. Measurements for PMMA were carried out in HPLC-grade DMF at 60.0 °C using three analytical columns (PSS GRAM; 10 µm; 20 × 50 mm; 1000, 1000, and 30 Å) with a PMMA calibration curve. Measurements for PDMA were undertaken in 0.1 M sodium nitrate solutions at 30.0 °C using one analytical column (PSS Suprema Lin XL; 10 µm; 8 × 300 mm; 100–3,000,000 Da). All runs were performed at 1.0 mL min^{-1} flow rate.

Thermogravimetric analysis (TGA): TGA measurements were conducted under air using a METTLER-TOLEDO TGA 3+ with temperature increasing from 25 °C to 800 °C at a rate of 10 °C min^{-1}.

Diffuse reflectance Ultraviolet-Visible (UV-Vis) absorption spectroscopy: diffuse reflectance UV-Vis absorption spectra of CMP NPs were recorded on a Perkin Elmer Lambda 900 with an integrating sphere.

Cyclic voltammetry (CV): CV measurements were conducted on a Metrohm Autolab PGSTAT204 potentiostat/galvanostat in a three-electrode-cell system under inert atmosphere: glassy carbon electrode as the working electrode, saturated calomel electrode as the reference electrode, and platinum wire as the counter electrode in 0.1 M NBu_4PF_6 in acetonitrile with a scan rate of 100 mV s^{-1} in the range of −2.0 eV to 2.0 eV. Before each measurement, a thin film of CMP NPs was deposited onto the glassy carbon electrode. Specifically, 100 µL of CMP NPs dispersion (5 mg/mL) was prepared either in ethanol (for CMP-0 and CMP-x NPs) or in acetone (for CMP-x-PMMA NPs), and mixed with 5 µL of Nafion® perfluorinated resin solution (5 wt%). The prepared dispersion (10 µL) was dropped onto the glassy carbon surface and dried at room temperature. This procedure was repeated three times to deposit a layer of CMP NPs. The thin film-modified glassy carbon electrode was put into the system after deoxygenating the solvent with argon purge for 5 min.

Dynamic light scattering (DLS) measurements: Hydrodynamic diameter was measured by Malvern Zetasizer Nano series. CMP-x-PMMA (0.1 mg/mL) and CMP-x-PDMA (0.1 mg/mL) samples were prepared in nitromethane and phosphate buffer (0.2 M, pH 7.4), respectively. The samples were sonicated for 10 minutes before each run. Average data were obtained from at least three runs of measurements.

Ⅱ. Photoredox Catalytic Reactions with CMP NPs

Photocatalytic [3+2] Cycloaddition of 4-Methoxyphenol (1) to *trans*-Anethole (2) with CMP NPs in Nitromethane

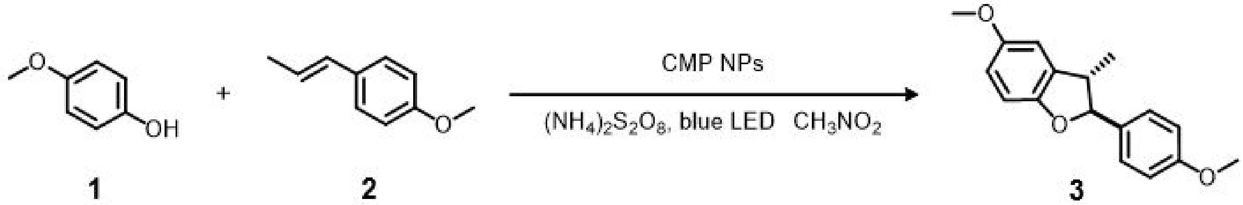

A 4 mL vial containing a stir bar was charged with 1 mg of CMP NPs (CMP-0, CMP-22-PMMA, CMP-40-PMMA, or CMP-55-PMMA) and 1 mL of nitromethane, and then sonicated using the bath sonicator for 10 min. To the suspension, 6.2 mg (0.05 mmol, 1.0 equiv.) of **1**, 11.1 mg (0.075 mmol, 1.5 equiv.) of **2**, and 22.8 mg (0.1 mmol, 2.0 equiv.) of $(NH_4)_2S_2O_8$ were added, and the vial was sealed with a septum screw cap. The reaction mixture was purged with nitrogen for 5 min and connected to a nitrogen-filled balloon. The vial was then positioned on the liquid cooled photoreactor (18 °C), where it was irradiated by 11.9 mW/cm^2 of blue light (λ_{max} = 460 nm). For control and opitmization experiments, the reaction mixture was sampled after 20 h. For kinetic monitoring experiments, aliquots (0.1 mL) of the reaction mixture were sampled after 2 h, 4 h, 6 h, 8 h, and 20 h. After centrifuging the samples, the supernatants were analzyed by ^{1}H NMR ($CDCl_3$, 700 MHz) with 1,3-dimethoxybenzene as an internal standard. The conversion was calculated from the integration ratios of 4H peak of **1** at 6.77 ppm to 1H peak of 1,3-dimethoxybenzene at 6.44 ppm. The product yield was calculated from the integration ratios of 1H peaks of the dihydrobenzofuran product (**3**) at 5.08 ppm and 3.42 ppm to 1H peak of 1,3-dimethoxybenzene at 6.44 ppm. For compound characterization, the reaction scale was doubled. After completion, the mixture was centrifuged, and transferred to a separatory funnel containing 20 mL of dichloromethane and 20 mL of water. The organic layers were separated while extracting three times with dichloromethane (20 mL). The combined organic layers were washed with brine (20 mL), dried over anhydrous $MgSO_4$, and concentrated by rotary evaporation. The crude product was purified using silica column chromatography (n-hexane : ethyl acetate = 5 : 95) to obtain 5-methoxy-2-(4-methoxyphenyl)-3-methyl-2,3-dihydrobenzofuran (**3**) as colorless oil. **^{1}H NMR** (400 MHz, $CDCl_3$): δ [ppm] 7.40 – 7.32 (m, 2H), 6.94 – 6.88 (m, 2H), 6.78 – 6.68 (m, 3H), 5.08 (d, J = 9.0 Hz, 1H), 3.82 (s, 3H), 3.78 (s, 3H), 3.47 – 3.35 (m, 1H), 1.38 (d, J = 6.9 Hz, 3H). **^{13}C NMR** (101 MHz, $CDCl_3$): δ [ppm] 159.79, 154.56, 153.42, 133.27, 132.85, 127.81, 114.15, 113.02, 110.24, 109.49, 92.75, 56.21, 55.47, 45.82, 17.73. **ESI-MS** *m/z*: 270 ($[M]^+$).The characterization data were consistent with literature values.[6,7]

Photocataltyic [2+2] Cylcoaddition of *trans*-Anethole (2) to Styrene (4) with CMP NPs in Nitromethane

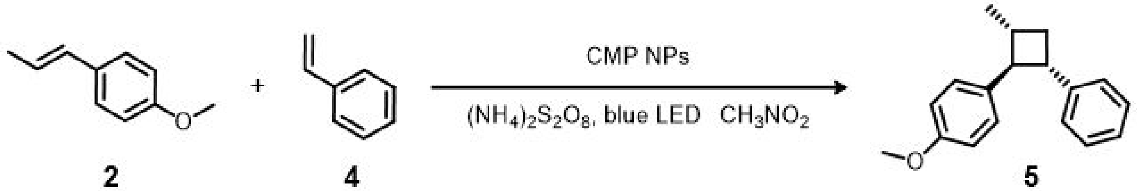

A 4 mL vial containing a stir bar was charged with 1 mg of CMP NPs (CMP-0, CMP-22-PMMA, CMP-40-PMMA, or CMP-55-PMMA) and 1 mL of nitromethane, and then sonicated using the bath sonicator for 10 min. To the suspension, 14.8 mg (0.1 mmol, 1.0 equiv.) of **2**, 45.6 mg (0.2 mmol, 2.0 equiv.) of $(NH_4)_2S_2O_8$, and 114.5 μL (1.0 mmol, 10 equiv.) of **4** were added, and the vial was sealed with a septum screw cap. The reaction mixture was purged with nitrogen for 5 min and connected to a nitrogen-filled balloon. The vial was then positioned on the liquid cooled photoreactor (18 °C), where it was irradiated by 11.9 mW/cm^2 of blue light (λ_{max} = 460 nm). For control and opitmization experiments, the reaction mixture was sampled after 20 h. For kinetic monitoring experiments, aliquots (0.1 mL) of the reaction mixture were sampled after 2 h, 4 h, 6 h, 8 h, and 20 h. After centrifuging the samples, the supernatants were analzyed by ^{1}H NMR ($CDCl_3$, 700 MHz) with 1,3-dimethoxybenzene as an internal standard. The conversion was calculated from the integration ratios of 1H peaks of **2** at 6.29 ppm and 6.07 ppm to 1H peak of 1,3-dimethoxybenzene at 6.44 ppm. The product yield was calculated from the integration ratios of 3H peak at 1.20 ppm, 1H peaks at 1.71 ppm, 2.34 ppm, 2.52 ppm, 2.96 ppm, and 3.40 ppm of the cycloaddition product (**5**) to 1H peak of 1,3-dimethoxybenzene at 6.44 ppm. For compound characterization, the reaction scale was doubled. After completion, the mixture was centrifuged, and the supernatant was transferred to a separatory funnel containing 20 mL of diethylether and 20 mL of water. The organic layers were separated while extracting three times with diethylether (10 mL). The combined organic layers were dried over anhydrous $MgSO_4$ and concentrated by rotary evaporation. The crude product was purified using silica column chromatography (n-hexane : dichloromethane = 6 : 1) to obtain 1-methoxy-4-((1*S*,2*R*,4*S*)-2-methyl-4-phenylcyclobutyl)benzene (**5**) as colorless oil. **^{1}H NMR** (400 MHz, $CDCl_3$): δ [ppm] 7.31 – 7.26 (m, 2H), 7.23 – 7.14 (m, 5H), 6.91 – 6.81 (m, 2H), 3.80 (s, 3H), 3.40 (q, *J* = 12.0 Hz, 1H), 2.96 (t, *J* = 9.6 Hz, 1H), 2.56 – 2.48 (m, 1H), 2.34 (m, 1H), 1.71 (q, *J* = 10.1 Hz, 1H), 1.20 (d, *J* = 6.5 Hz, 3H). **^{13}C NMR** (101 MHz, $CDCl_3$): δ [ppm] 158.21, 144.80, 135.99, 128.37, 127.92, 126.76, 126.05, 113.90, 55.67, 55.40, 44.24, 35.64, 34.07, 20.61. **APCI-MS** *m/z*: 253 ([M+H]$^+$). The characterization data were consistent with literature values.[8,9]

Photocatalytic Aza-Henry Reaction of *N*-Phenyltetrahydroisoquinoline (6) with CMP NPs in Nitromethane

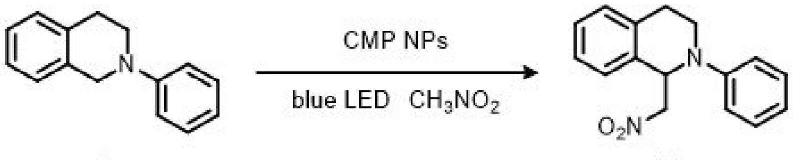

A 4 mL vial containing a stir bar was charged with 1 mg of CMP NPs (CMP-0, CMP-22-PMMA, CMP-40-PMMA, or CMP-55-PMMA) and 1 mL of nitromethane, and then sonicated using the bath sonicator for 10 min. To the suspension, 10.5 mg (0.05 mmol, 1.0 equiv.) of **6**[10] was added, and the vial was sealed with a septum screw cap. The reaction mixture was purged with nitrogen for 5 min and connected to a nitrogen-filled balloon. The vial was then positioned on the liquid cooled photoreactor (18 °C), where it was irradiated by 5.4 mW/cm^2 of blue light (λ_{max} = 460 nm). For control and opitmization experiments, the reaction mixture was sampled after 20 h. For kinetic monitoring experiments, aliquots (0.1 mL) of the reaction mixture were sampled after 2 h, 4 h, 6 h, 8 h, and 20 h. After centrifuging the samples, the supernatants were analzyed by ^{1}H NMR ($CDCl_3$, 700 MHz) with 1,3-dimethoxybenzene as an internal standard. The conversion was calculated from the integration ratios of 2H peaks of **6** at 4.34 ppm, 3.50 ppm, and 2.93 ppm to 1H peak of 1,3-dimethoxybenzene at 6.44 ppm. The product yield was calculated from the integration ratios of 1H peaks of the product (**7**) at 5.55 ppm, 4.88 ppm, 4.57 ppm, and 2.80 ppm to 1H peak of 1,3-dimethoxybenzene at 6.44 ppm. For compound characterization, the reaction scale was doubled. After completion, the mixture was centrifuged, and the supernatant was transferred to a separatory funnel containing 10 mL of ethyl acetate and 10 mL of water. The organic layers were separated while extracting three times with ethyl acetate (10 mL). The combined organic layers were dried over anhydrous $MgSO_4$ and concentrated by rotary evaporation. The crude product was purified using silica column chromatography (n-hexane : ethyl acetate = 95 : 5) to obtain 1-(nitromethyl)-2-phenyl-1,2,3,4-tetrahydroisoquinoline (**7**) as yellow oil. **^{1}H NMR** (400 MHz, $CDCl_3$): δ [ppm] 7.30 – 7.26 (m, 2H), 7.26 – 7.20 (m, 2H), 7.20 – 7.12 (m, 2H), 6.98 (d, *J* = 7.9 Hz, 2H), 6.85 (tt, *J* = 7.3, 1.0 Hz, 1H), 5.55 (t, *J* = 7.2 Hz, 1H), 4.88 (dd, *J* = 11.8, 7.8 Hz, 1H), 4.57 (dd, *J* = 11.8, 6.7 Hz, 1H), 3.71 – 3.58 (m, 2H), 3.15 – 3.03 (m, 1H), 2.80 (dt, *J* = 16.2, 5.0 Hz, 1H). **^{13}C NMR** (101 MHz, $CDCl_3$): δ [ppm] 148.57, 135.42, 133.08, 129.66, 129.34, 128.27, 127.15, 126.86, 119.59, 115.26, 78.94, 58.35, 42.24, 26.62. **ESI-MS** *m/z*: 269 ($[M+H]^+$). The characterization data were consistent with literature values.[10,11]

Photocatalytic Thiol-Ene Reaction of *N*-Acetyl-*L*-Cysteine (8) and 3-Allyloxy-1,2-Propanediol (9) with CMP NPs in Aqueous Buffer Solutions

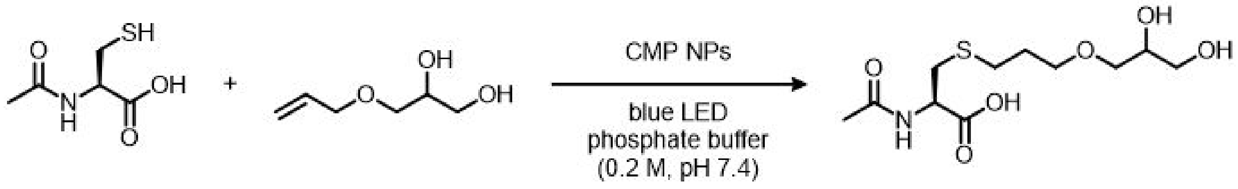

A 4 mL vial containing a stir bar was charged with 1 mg of CMP NPs (CMP-0, CMP-55, CMP-22-PDMA, CMP-40-PDMA, or CMP-55-PDMA) and 1 mL of pH 7.4 phosphate buffer (0.2 M), and then sonicated using the bath sonicator for 10 min. To the suspension, 16.3 mg (0.1 mmol, 1.0 equiv.) of **8** and 24.7 mg (0.2 mmol, 2.0 equiv.) of **9** were added, and the vial was sealed with a septum screw cap. The reaction mixture was purged with nitrogen for 5 min and connected to a nitrogen-filled balloon. The vial was then positioned on the liquid cooled photoreactor (18 °C), where it was irradiated by 11.9 mW/cm^2 of blue light (λ_{max} = 460 nm). For control and opitmization experiments, the reaction mixture was sampled after 2 h. For kinetic monitoring experiments, aliquots (0.1 mL) of the reaction mixture were sampled after 0.5 h, 1 h, 1.5 h, and 2 h. After centrifuging the samples, the supernatants were analzyed by ^{1}H NMR (D_2O, 700 MHz) with dimethyl sulfone as an internal standard. The conversion was calculated from the integration ratio of 2H peak of **8** at 2.92 – 2.91 ppm to 6H peak of dimethyl sulfone at 3.15 ppm. The product yield was calculated from the integration ratios of 2H peaks of the thiol-ene reaction product (**10**) at 2.66 ppm and 1.89 ppm to 6H peak of dimethyl sulfone at 3.15 ppm. For compound characterization, the reaction scale was doubled. After completion, the mixture was centrifuged, and the supernatant was directly loaded onto a column of Sephadex LH-20 for exchanging the solvent to ethyl acetate : methanol = 6 : 4. The fractions including the product were concentrated by rotary evaporation. The crude product was purified using silica column chromatography (ethyl acetate : methanol = 6 : 4) to obtain *N*-acetyl-*S*-(3-(2,3-dihydroxypropoxy)propyl)-*L*-cysteine (**10**) as colorless oil. **^{1}H NMR** (400 MHz, D_2O): δ [ppm] 4.37 (dd, *J* = 8.2, 4.3 Hz, 1H), 3.92 – 3.86 (m, 1H), 3.67 – 3.47 (m, 6H), 3.06 (dd, *J* = 13.8, 4.3 Hz, 1H), 2.88 (dd, *J* = 13.8, 8.2 Hz, 1H), 2.66 (t, *J* = 7.2 Hz, 2H), 2.06 (s, 3H), 1.92 – 1.84 (m, 2H). **^{13}C NMR** (101 MHz, D_2O): δ [ppm] 176.99, 173.65, 71.28, 70.35, 69.63, 62.65, 54.49, 33.77, 28.50, 28.17, 21.90. **HRMS** (ESI) calculated for $C_{11}H_{21}NO_6SNa$ $[M+Na]^+$: 318.0987; Found: 318.1122.

Photocatalytic Thiol-Ene Reaction to Synthesize *N*[5]-((2*R*)-1-((Carboxymethyl)amino)-3-((3-(2,3-dihydroxypropoxy)propyl)thio)-1-oxopropan-2-yl)-*L*-Glutamine (11)

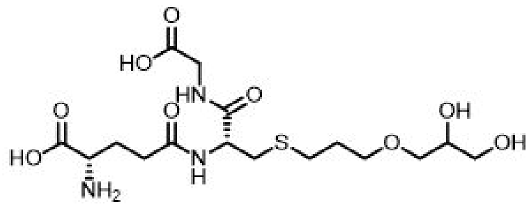

11

A 4 mL vial containing a stir bar was charged with 2 mg of CMP-22-PDMA and 2 mL of pH 7.4 phosphate buffer (0.2 M), and then sonicated using the bath sonicator for 10 min. To the suspension, 61.4 mg (0.2 mmol, 1.0 equiv.) of *L*-glutathione and 49.4 mg (0.4 mmol, 2.0 equiv.) of **9** were added, and the vial was sealed with a septum screw cap. The reaction mixture was purged with nitrogen for 5 min and connected to a nitrogen-filled balloon. The vial was then positioned on the liquid cooled photoreactor (18 °C), where it was irradiated by 11.9 mW/cm^2 of blue light (λ_{max} = 460 nm) for 2 h. After completion, the reaction mixture was centrifuged, and 0.1 mL of the supernatant was used for ^1H NMR anlaysis (D_2O, 700 MHz) with dimethyl sulfone as an internal standard. The conversion was calculated from the integration ratio of 2H peak of *L*-glutathione at 2.97 – 2.93 ppm to 6H peak of dimethyl sulfone at 3.15 ppm. The product yield was calculated from the integration ratios of 2H peaks of the thiol-ene reaction product (**11**) at 2.66 ppm and 1.87 ppm to 6H peak of dimethyl sulfone at 3.15 ppm. For compound characterization, the residual supernatant was directly loaded onto a column of Sephadex LH-20 for exchanging the solvent to methanol. The fractions including the product were concentrated by rotary evaporation. The crude product was purified using silica column chromatography (100% methanol) to obtain **11** as colorless oil. **^{1}H NMR** (400 MHz, D_2O): δ [ppm] 4.59 (dd, *J* = 8.9, 4.9 Hz, 1H), 3.93 – 3.86 (m, 1H), 3.81 – 3.76 (m, 3H), 3.68 – 3.47 (m, 6H), 3.10 (dd, *J* = 14.1, 5.0 Hz, 1H), 2.89 (dd, *J* = 14.1, 9.0 Hz, 1H), 2.67 (td, *J* = 7.2, 1.5 Hz, 2H), 2.55 (td, *J* = 7.5, 2.3 Hz, 2H), 2.22 – 2.12 (m, 2H), 1.93 – 1.84 (m, 2H). **^{13}C NMR** (101 MHz, D_2O): δ [ppm] 176.18, 174.92, 173.94, 171.98, 71.30, 70.35, 69.58, 62.66, 54.10, 53.11, 43.34, 32.93, 31.41, 28.46, 28.13, 26.19. **HRMS** (ESI) calculated for $C_{16}H_{29}N_3O_9SNa$ $[M+Na]^+$: 462.1522; Found: 462.1740.

Photocatalytic Thiol-Ene Reaction to Synthesize Methyl 3-((3-(2,3-Dihydroxypropoxy)propyl)thio) propanoate (12)

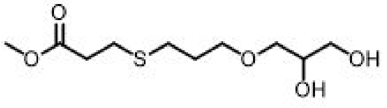

12

A 4 mL vial containing a stir bar was charged with 2 mg of CMP-22-PDMA and 2 mL of pH 7.4 phosphate buffer (0.2 M), and then sonicated using the bath sonicator for 10 min. To the suspension, 22.2 μL (0.2

mmol, 1.0 equiv.) of methyl 3-mercaptopropionate and 49.4 mg (0.4 mmol, 2.0 equiv.) of **9** were added, and the vial was sealed with a septum screw cap. The reaction mixture was purged with nitrogen for 5 min and connected to a nitrogen-filled balloon. The vial was then positioned on the liquid cooled photoreactor (18 °C), where it was irradiated by 11.9 mW/cm^2 of blue light (λ_{max} = 460 nm) for 2 h. After completion, the reaction mixture was centrifuged, and 0.1 mL of the supernatant was used for ^{1}H NMR anlaysis (D_2O, 700 MHz) with dimethyl sulfone as an internal standard. The conversion was calculated from the integration ratio of 2H peak of methyl 3-mercaptopropionate at 2.79 – 2.76 ppm to 6H peak of dimethyl sulfone at 3.15 ppm. The product yield was calculated from the integration ratios of 2H peaks of the thiol-ene reaction product (**12**) at 2.67 ppm and 1.87 ppm to 6H peak of dimethyl sulfone at 3.15 ppm. For compound characterization, the residual supernatant was directly loaded onto a column of Sephadex LH-20 for exchanging the solvent to methanol. The fractions including the product were concentrated by rotary evaporation. The crude product was purified using silica column chromatography (100% ethyl acetate) to obtain **12** as colorless oil. **^{1}H NMR** (400 MHz, D_2O): δ [ppm] 3.92 – 3.86 (m, 1H), 3.74 (s, 3H), 3.68 – 3.47 (m, 6H), 2.85 (td, *J* = 6.8, 1.1 Hz, 2H), 2.74 (td, *J* = 6.8, 1.0 Hz, 2H), 2.67 (t, *J* = 7.3 Hz, 2H), 1.94 – 1.83 (m, 2H). **^{13}C NMR** (101 MHz, D_2O): δ [ppm] 175.36, 71.30, 70.35, 69.64, 62.66, 52.29, 34.07, 28.46, 27.68, 26.19. **HRMS** (ESI) calculated for $C_{10}H_{20}O_5SNa$ $[M+Na]^+$: 275.0929; Found: 275.1018.

Photocatalytic Thiol-Ene Reaction to Synthesize *N*-Acetyl-*S*-(3-Ureidopropyl)-*L*-Cysteine (13)

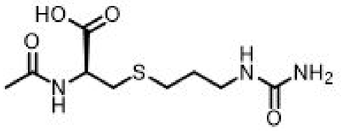

13

A 4 mL vial containing a stir bar was charged with 2 mg of CMP-22-PDMA and 2 mL of pH 7.4 phosphate buffer (0.2 M), and then sonicated using the bath sonicator for 10 min. To the suspension, 32.6 mg (0.2 mmol, 1.0 equiv.) of **8** and 40 mg (0.4 mmol, 2.0 equiv.) of allylurea were added, and the vial was sealed with a septum screw cap. The reaction mixture was purged with nitrogen for 5 min and connected to a nitrogen-filled balloon. The vial was then positioned on the liquid cooled photoreactor (18 °C), where it was irradiated by 11.9 mW/cm^2 of blue light (λ_{max} = 460 nm) for 2 h. After completion, the reaction mixture was centrifuged, and 0.1 mL of the supernatant was used for ^{1}H NMR anlaysis (D_2O, 700 MHz) with dimethyl sulfone as an internal standard. The conversion was calculated from the integration ratio of 2H peak of **8** at 2.92 – 2.91 ppm to 6H peak of dimethyl sulfone at 3.15 ppm. The product yield was calculated from the integration ratios of 2H peaks of the thiol-ene reaction product (**13**) at 2.62 ppm and 1.78 ppm to 6H peak of dimethyl sulfone at 3.15 ppm. For compound characterization, the residual supernatant was directly loaded onto a column of Sephadex LH-20 for exchanging the solvent to ethyl acetate : methanol = 6 : 4. The fractions including the product were concentrated by rotary evaporation.

The crude product was purified using silica column chromatography (ethyl acetate : methanol = 6 : 4) to obtain **13** as colorless oil. **^{1}H NMR** (400 MHz, D_2O): δ [ppm] 4.38 (dd, J = 8.2, 4.3 Hz, 1H), 3.19 (t, J = 6.7 Hz, 2H), 3.05 (dd, J = 13.9, 4.4 Hz, 1H), 2.88 (dd, J = 13.9, 8.2 Hz, 1H), 2.62 (t, J = 7.3 Hz, 2H), 2.06 (s, 3H), 1.78 (dt, J = 7.7, 6.6 Hz, 2H). **^{13}C NMR** (101 MHz, D_2O): δ [ppm] 176.94, 173.68, 161.53, 54.43, 33.67, 28.85, 28.76, 21.88. **HRMS** (ESI) calculated for $C_9H_{17}O_4SNa$ $[M+Na]^+$: 286.0837; Found: 286.0911.

Recyclability Tests with Photocatalytic Thiol-Ene Reaction of 8 and 9 with CMP-22-PDMA

A 4 mL vial containing a stir bar was charged with 1 mg of CMP-22-PDMA (fresh or used) and 1 mL of pH 7.4 phosphate buffer (0.2 M), and then sonicated using the bath sonicator for 10 min. To the suspension, 16.3 mg (0.1 mmol, 1.0 equiv.) of **8** and 24.7 mg (0.2 mmol, 2.0 equiv.) of **9** were added, and the vial was sealed with a septum screw cap. The reaction mixture was purged with nitrogen for 5 min and connected to a nitrogen-filled balloon. The vial was then positioned on the liquid cooled photoreactor (18 °C), where it was irradiated by 11.9 mW/cm^2 of blue light (λ_{max} = 460 nm) for 2 h. After completion, the reaction mixture was centrifuged, and 0.1 mL of the supernatant was used for ^{1}H NMR anlaysis (D_2O, 700 MHz) with dimethyl sulfone as an internal standard. The conversion and product yield were calculated as described above. The precipitated CMP-22-PDMA was washed by three rounds of centrifugation (21,100 × g, 10 min) with deionized water refill (1 mL) and reused for the next cycle of reaction. After the 5th reaction cycle, the precipitated CMP-22-PDMA was washed by total five rounds of centifugation (21,100 × g, 10 min) with three times of deionized water refill (1 mL) and then two times of ethanol refill (1 mL). The final pellet was dried under high vacuum overnight before FT-IR measurements.

Ⅲ. Supplementary Figures

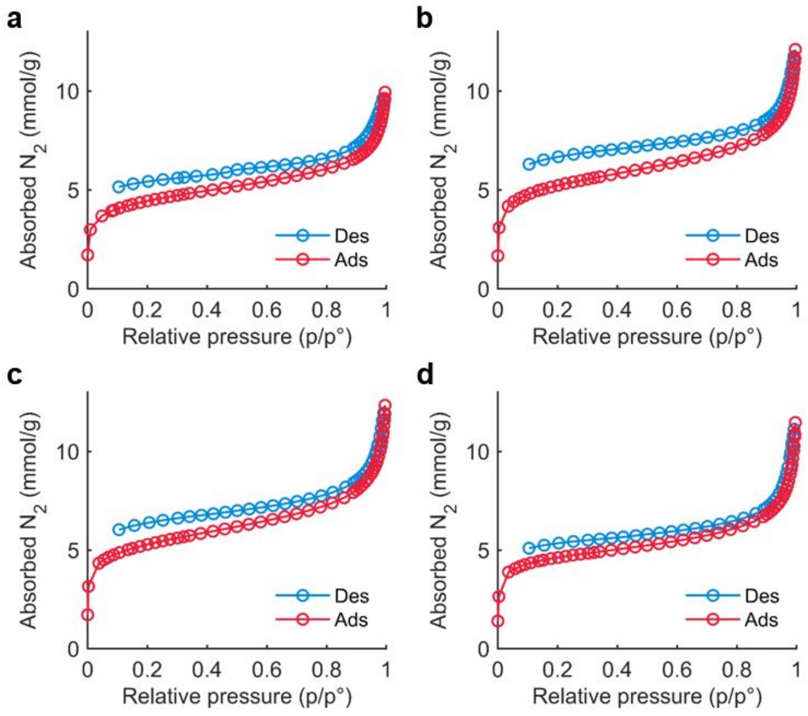

Figure S1. N_2 sorption–desorption isotherms of (a) CMP-0, (b) CMP-22, (c) CMP-40, and (d) CMP-55 NPs.

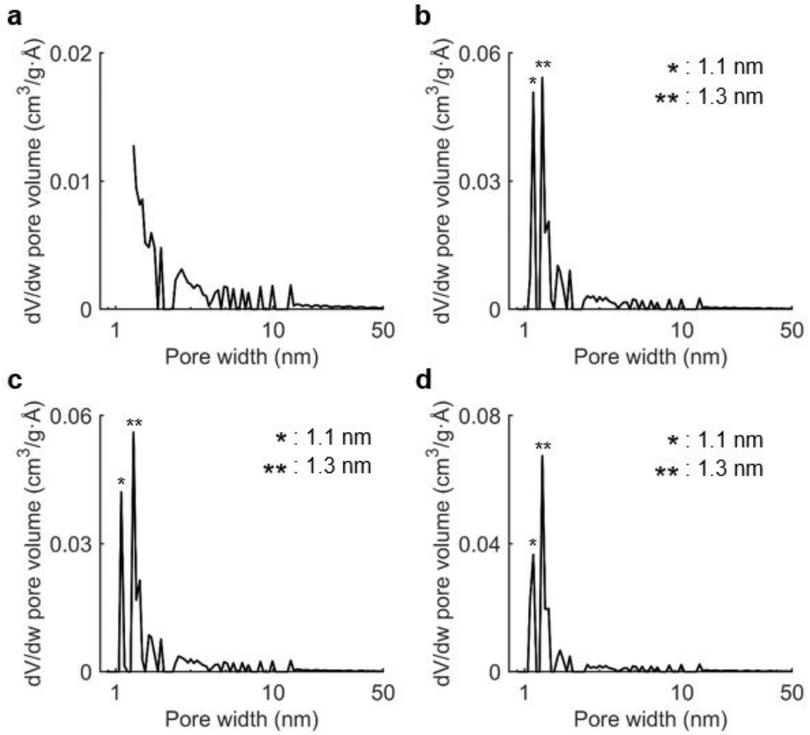

Figure S2. Pore size distribution curves of (a) CMP-0, (b) CMP-22, (c) CMP-40, and (d) CMP-55 NPs. The population of the pores are centered at 1.1 nm and 1.3 nm. As the benzylamine content increases in the CMP-x NPs ($x = 22 < 40 < 55$), the population ratio of the 1.3 nm pores to the 1.1 nm pores increases.

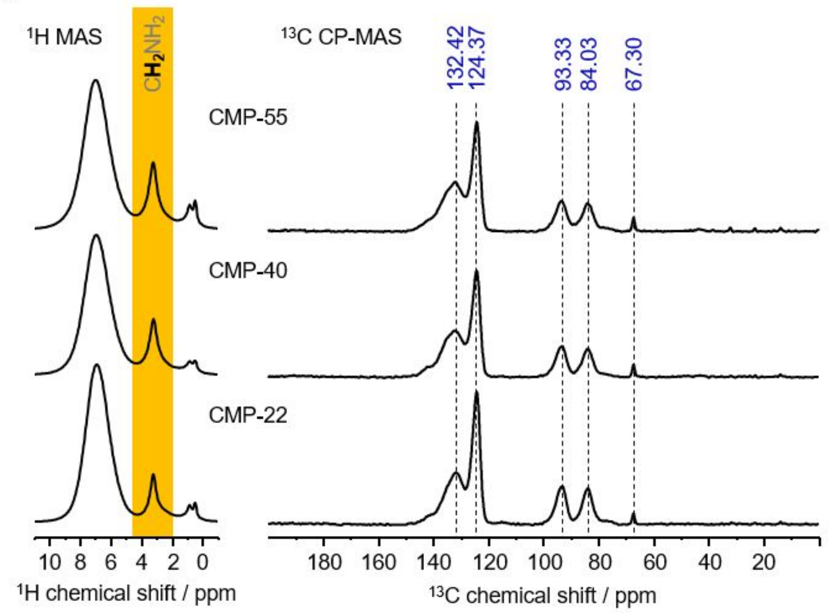

b

CMP	molar ratio of aromatic protons : CH_2-N protons
CMP-22	0.890 : 0.110
CMP-40	0.835 : 0.165
CMP-55	0.815 : 0.185

c

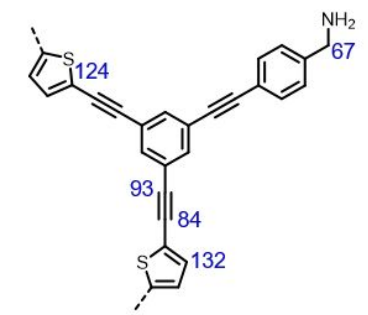

Figure S3. Solid-state ^{1}H MAS and ^{13}C CP-MAS NMR analysis of CMP NPs. (a) Solid-state NMR spectra of CMP-22, CMP-40, and CMP-55 NPs. The integration of benzylamine CH_2-N protons (2–4 ppm) in the ^{1}H NMR spectra showed that more benzylamine groups were incorporated into the CMP NPs made of more monomer **C** (CMP-55 > CMP-40 > CMP-22). (b) Molar ratio of aromatic protons (5–9 ppm) to benzylamine CH_2-N protons calculated from the ^{1}H NMR spectra. Assuming that dangling benzylamine aromatic protons may be ignored in the solid-state ^{1}H NMR signals, the molar ratios of monomers (B : C) integrated into the CMP NPs were calculated as follows. When molar fractions of the monomers **A**, **B**, and **C** in the CMP NPs are x, y, and z, respectively, $x+y+z = 1$. From the ^{1}H NMR integration ratios, $3x+2y : 2z$ = the number of aromatic protons : the number of CH_2–N protons = A : 1−A. Assuming the similar reactivity of **B** and **C** towards **A** and excluding oligomers, $3x = 2y+z$. By solving the three equations above, molar ratios of **B** to **C** integrated into the CMP network could be calculated by $y/z = 0.5A/(1-A)-1/4$. The corrected molar feed ratios were calculated by subtracting oligomer fractions as follows. When molar fractions of monomers **B** and **C** in the feed are a and b, $a+b = 1$. The oligomers are formed when one molecule of **A** reacts with three molecules of **C** and any chains are terminated by **A** reacted with two molecules of **C**. Therefore, the probability of forming oligomers is b^2, and the corrected molar feed ratios of **B** : **C** are $a/(1-b^2)$: $(b-b^2)/(1-b^2)$. (c) Peak assignment of solid-state ^{13}C CP-MAS NMR of CMP NPs.

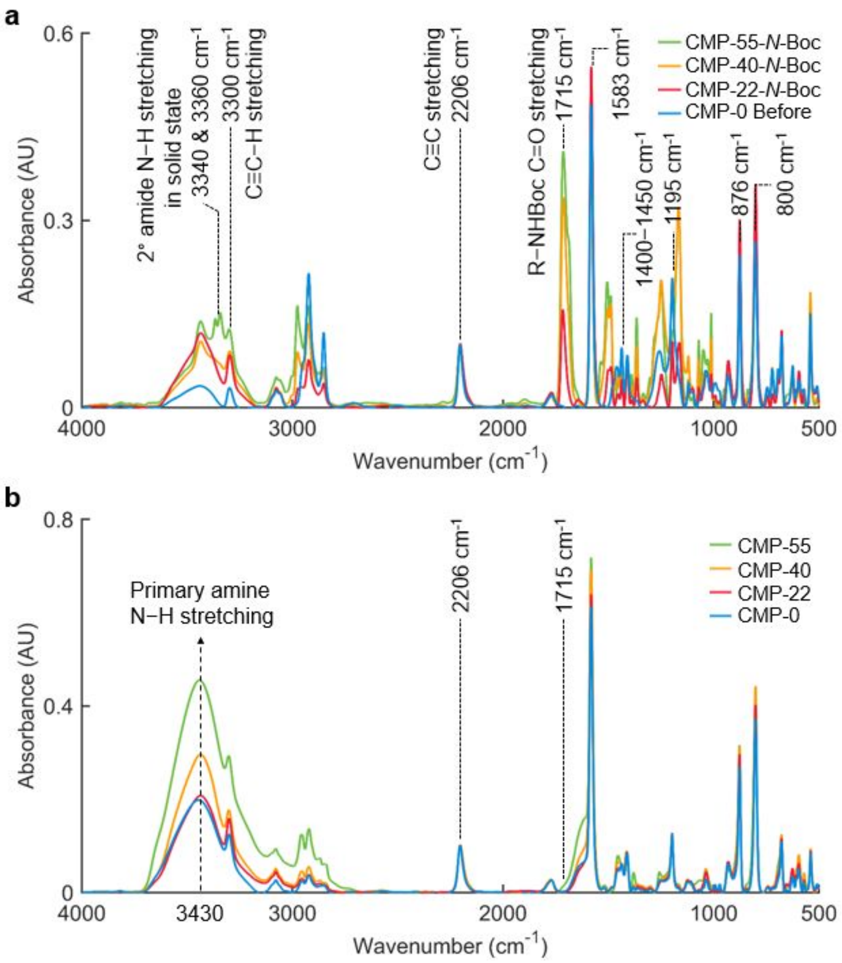

Figure S4. FT-IR spectra of CMP NPs (a) before and (b) after *N*-Boc deprotection. For quantitative comparison of the spectra, the absorbance values at 2206 cm^{-1} were normalized to 0.1. Comparison of the spectra above demonstrated that C=O stretching (1715 cm^{-1}) and N–H stretching (3340 cm^{-1} and 3360 cm^{-1}) signals of R–NHBoc were completely disappeared after the *N*-Boc deprotection while primary amine N–H stretching (3430 cm^{-1}) signals were generated. It should be noted that the strong O–H stretching (3550–3200 cm^{-1}) signals in CMP-0 and CMP-x NPs could result from residual water or ethanol in the samples.

a

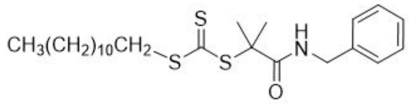

CTA-benzylamine conjugate

b

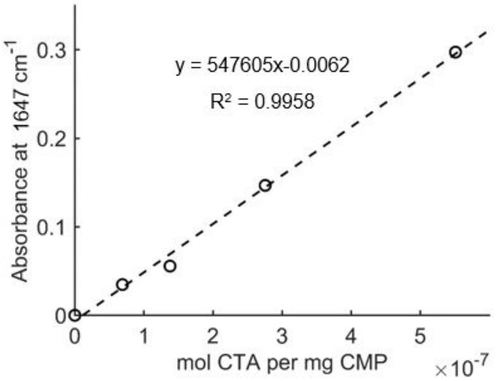

c

CMP NPs	Absorbance at 1675 cm^{-1}	mol CTA per mg CMP	Surface density (# of CTA/nm^2)
CMP-22-CTA	0.0420	8.80×10^{-8} mol/mg CMP	1.92
CMP-40-CTA	0.1064	2.06×10^{-7} mol/mg CMP	4.39
CMP-55-CTA	0.1349	2.58×10^{-7} mol/mg CMP	5.75

Figure S5. Quantification of the immobilized CTA in CMP-x-CTA NPs. (a) Structure of CTA-benzylamine conjugate. This model compound was synthesized through the same procedure for functionalization of CMP NPs with CTA, and used to generate a calibration curve for quantifying the immobilized CTA on the CMP NPs. We assumed that the C=O stretching absorbance from its amide of the model compound would be similar to that of the amide formed by the CTA-COOH + NH_2-CMP coupling reaction although the absorbance peak wavenumber could be shifted. (b) Calibration curve for quantifying CTA molecules per mg CMP-x-CTA NPs. The stock mixture of CTA-benzylamine in KBr was prepared by mixing 2 mg of CTA-benzylamine and 360 mg of KBr. FT-IR pellets were prepared by mixing 2 mg of diluted CTA-benzylamine/KBr mixture and 360 mg of KBr. (c) The estimated number of immobilized CTA molecules in CMP-x-CTA NPs from the calibration curve. Given that CMP-22, CMP-40, and CMP-55 NPs could contain up to 1.307×10^{-6} mol, 2.333×10^{-6} mol, and 3.266×10^{-6} mol benzylamine groups per mg CMP, respectively, less than 10% of all primary amines in the CMP-x NPs were accessible for the CTA coupling presumably due to inefficient mass transfer into the cores. Details for the surface density calculation is available in the Methods section.

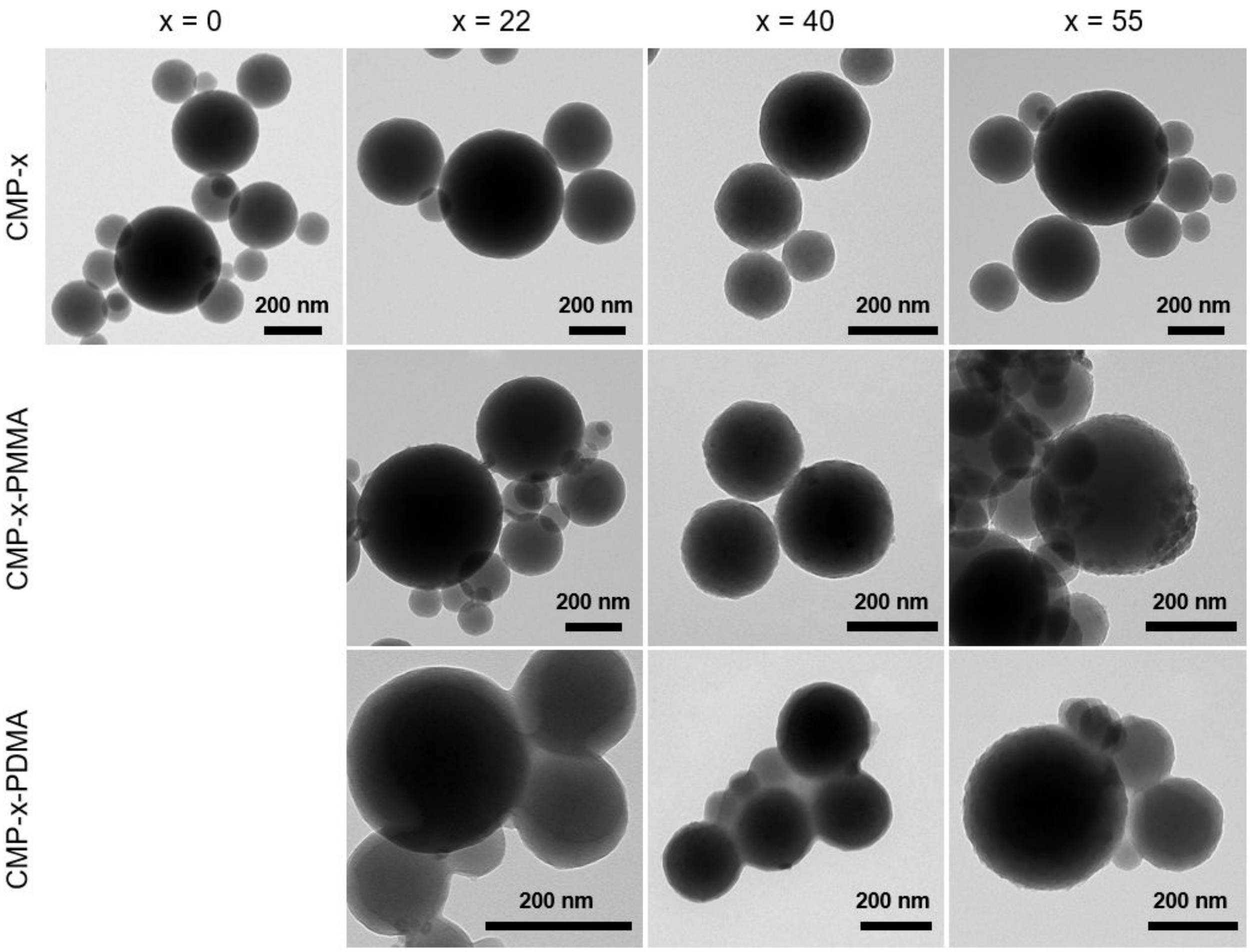

Figure S6. TEM images of CMP NPs before and after polymer grafting. The bumps on the surface are more discernible in PMMA-grafted ones due to the longer chains (M_n > 26000 g mol−1) than that of PDMA-grafted CMP NPs (M_n < 6500 g mol−1).

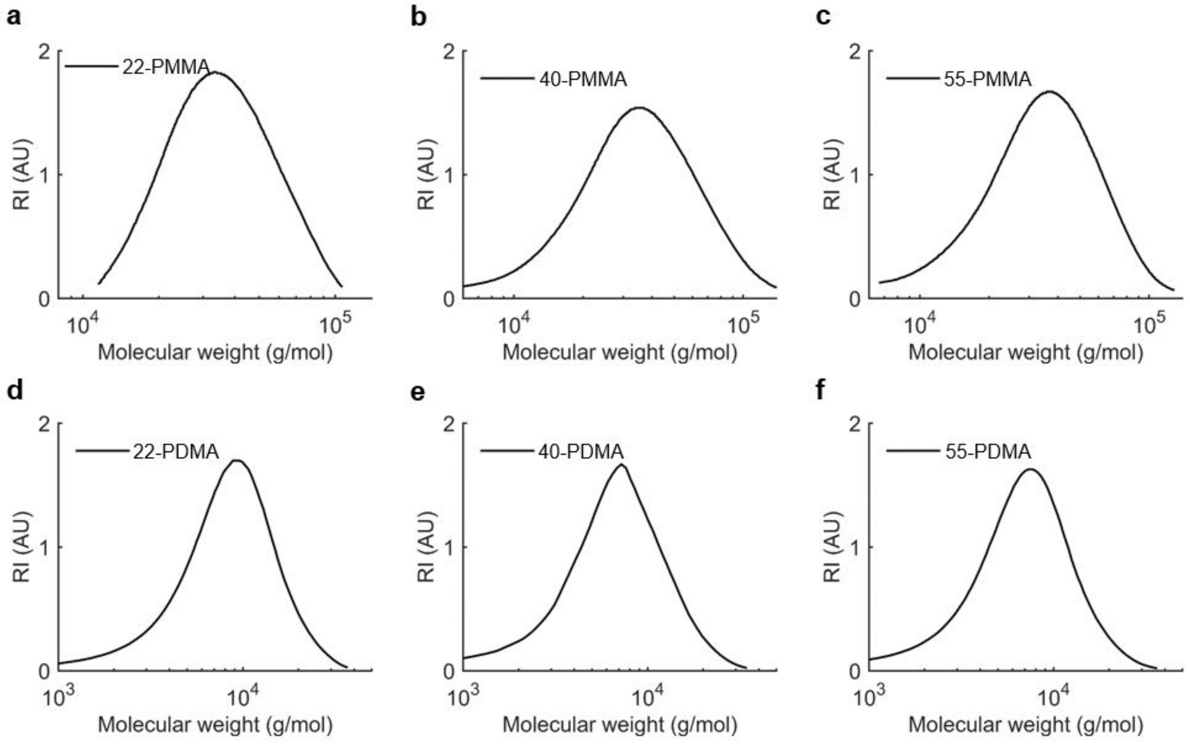

Figure S7. Molecular weight distribution curves of free polymers generated during the synthesis of hairy CMP NPs: (a) CMP-22-PMMA, (b) CMP-40-PMMA, (c) CMP-55-PMMA, (d) CMP-22-PDMA, (e) CMP-40-PDMA, and (f) CMP-55-PDMA.

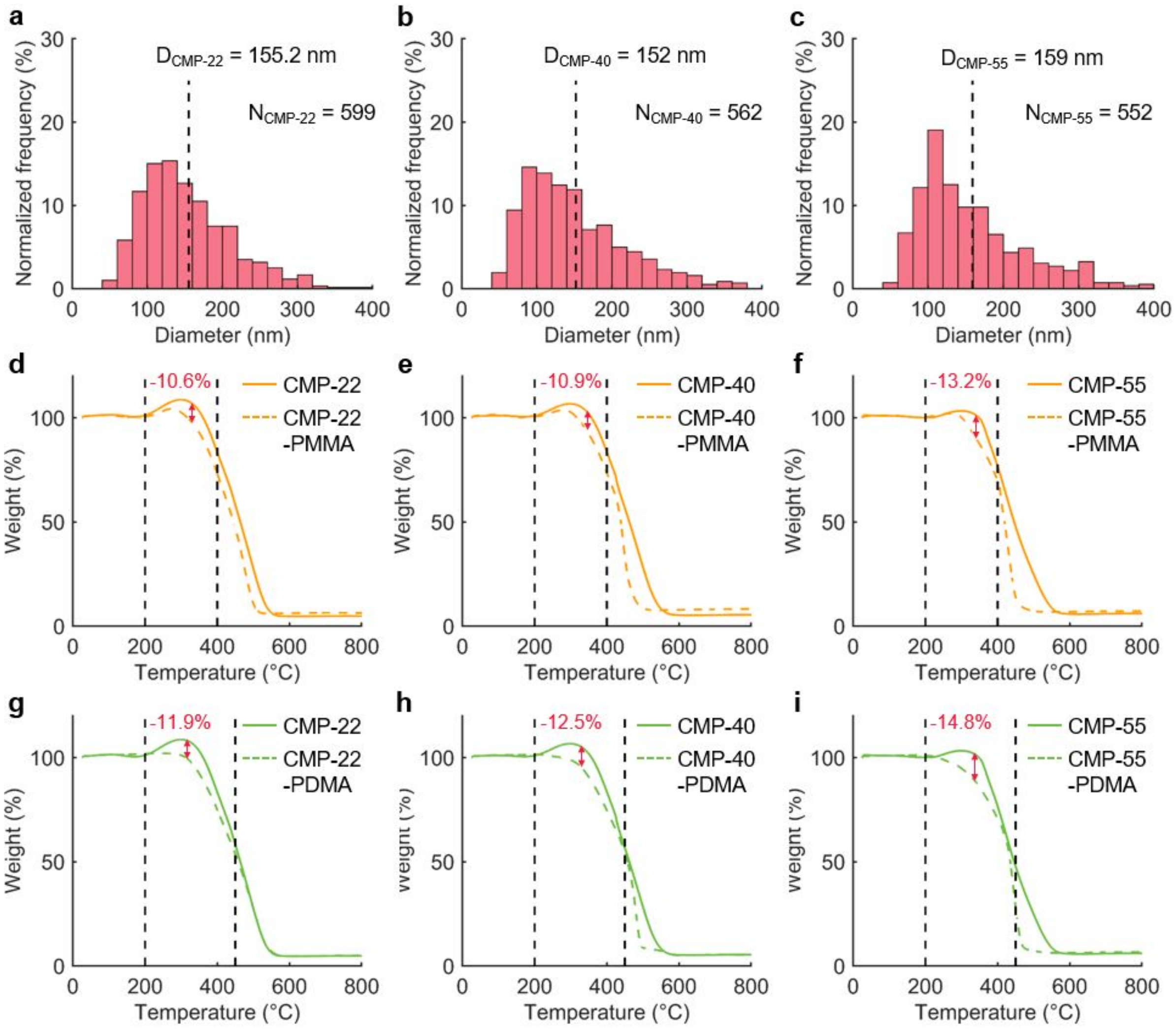

Figure S8. Average size of CMP-x NPs and polymer weight fractions in hairy CMP NPs. Size distribution curves of (a) CMP-22, (b) CMP-40, and (c) CMP-55. Using ImageJ software, size distribution and average size of NPs were determined from TEM images with 599 CMP-22, 562 CMP-40, and 552 CMP-55 NPs. TGA curves of (d) CMP-22 and CMP-22-PMMA, (e) CMP-40 and CMP-40-PMMA, (f) CMP-55 and CMP-55-PMMA, (g) CMP-22 and CMP-22-PDMA, (h) CMP-40 and CMP-40-PDMA, and (i) CMP-55 and CMP-55-PDMA. By comparing the TGA curves of CMP NPs before and after polymer grafting in the range where thermal degradation of PMMA (200–400 °C)[12,13] and PDMA (200–450 °C)[14] occur, the weight fractions of grafted polymers in the hairy CMP NPs were determined at the maximum mass difference points between CMP-x and hairy CMP-x NPs: 10.6 wt% PMMA in CMP-22-PMMA; 10.9 wt% PMMA in CMP-40-PMMA; 13.2 wt% PMMA in CMP-55-PMMA; 11.9 wt% PDMA in CMP-22-PDMA; 12.5 wt% PDMA in CMP-40-PDMA; 14.8 wt% PDMA in CMP-55-PDMA. The residual material at 800 °C could be

ash containing minerals and respective oxides resulted from small amounts of residual inorganic salts such as sodium sulfate and sodium chloride in the CMP NPs during their preparation.

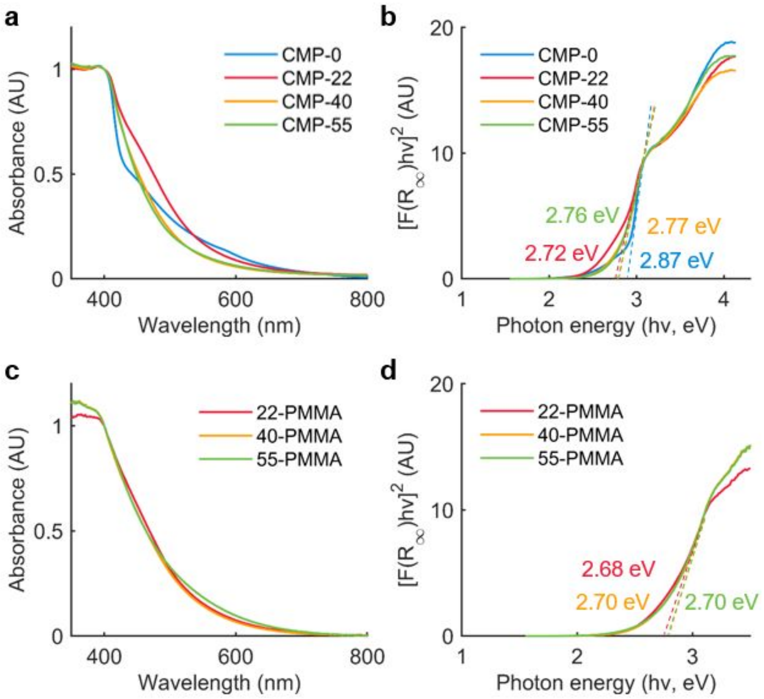

Figure S9. Determination of optical band gaps of CMP-x NPs and PMMA-grafted CMP NPs using Tauc relation for a direct band gap material.[15] (a) Absorbance spectra of CMP-x NPs were obtained using Kubelka-Munk function, F(R_∞), from diffuse reflectance spectra. The maximum absorption wavelength (λ_{max} = 391 nm) of the CMP-x NPs remained unchanged despite the increasing molar fractions of benzylamine. (b) Tauc plot of the CMP-x NPs for a direct band gap material. (c) Absorbance spectra of CMP-x-PMMA NPs. After immobilizing CTA and grafting polymers, the absorbance spectra ($\lambda > 400$ nm) look more similar despite the different surface grafting density. (d) Tauc plot of the CMP-x-PMMA NPs for a direct band gap material.

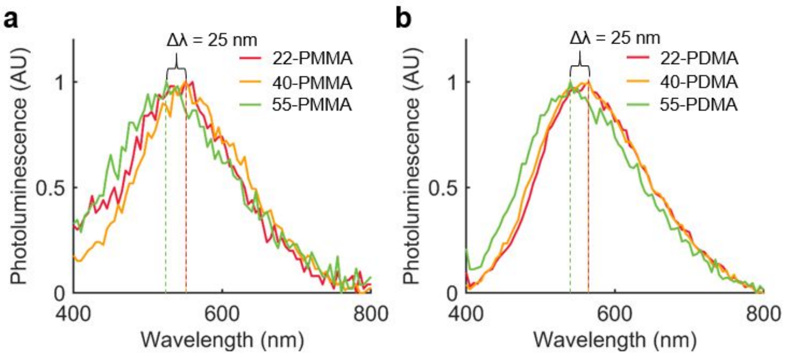

Figure S10. Fluorescence emission spectra of polymer-grafted CMP NPs. Measured using infinite M1000 plate reader. (a) CMP-x-PMMA NPs in nitromethane. (b) CMP-x-PDMA NPs in phosphate buffer solutions (0.2 M, pH 7.4). Polymer-grafted CMP-55 NPs exhibited approximately 25 nm of blue shift in the spectra.

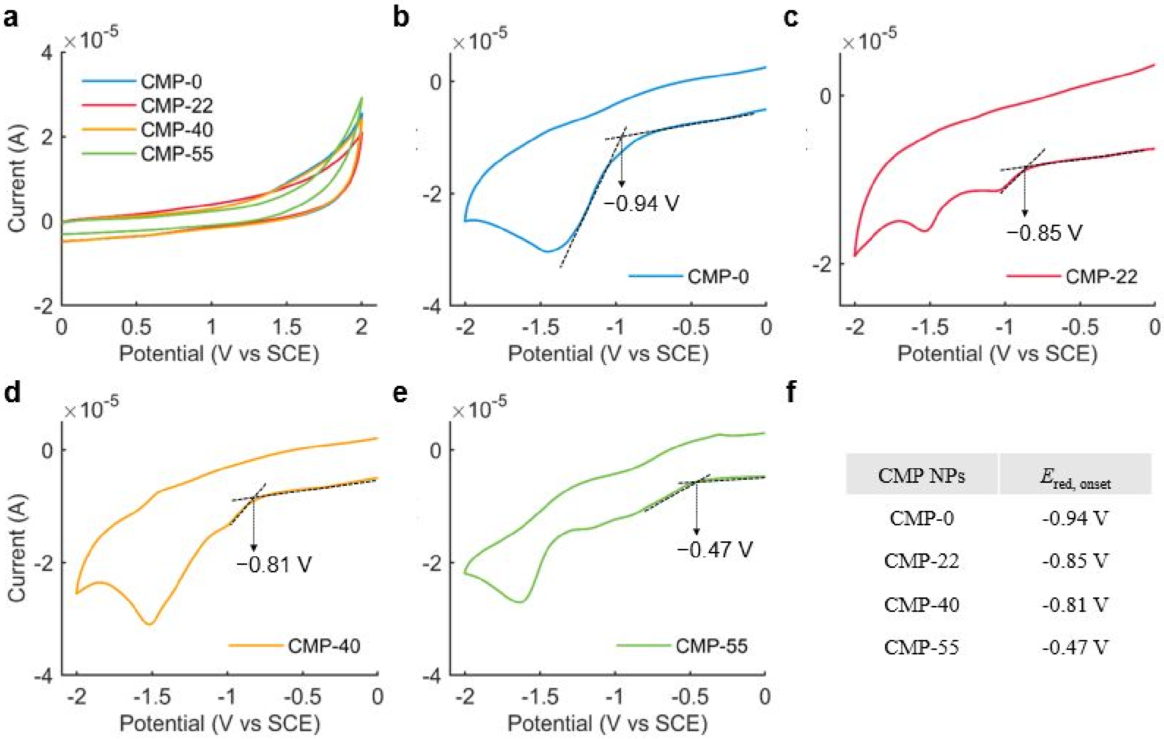

Figure S11. Cyclic voltammograms of CMP-x NPs in the (a) oxidation cycles and (b–e) reduction cycles. (a) No oxidation peaks were observed within the potential window (−2.0 V to +2.0 V vs SCE) of 0.1 M NBu_4PF_6 in acetonitrile. (b) CMP-0. (c) CMP-22. (d) CMP-40. (e) CMP-55. (f) Reduction onset potential of CMP-x NPs was used to estimate the LUMO levels.

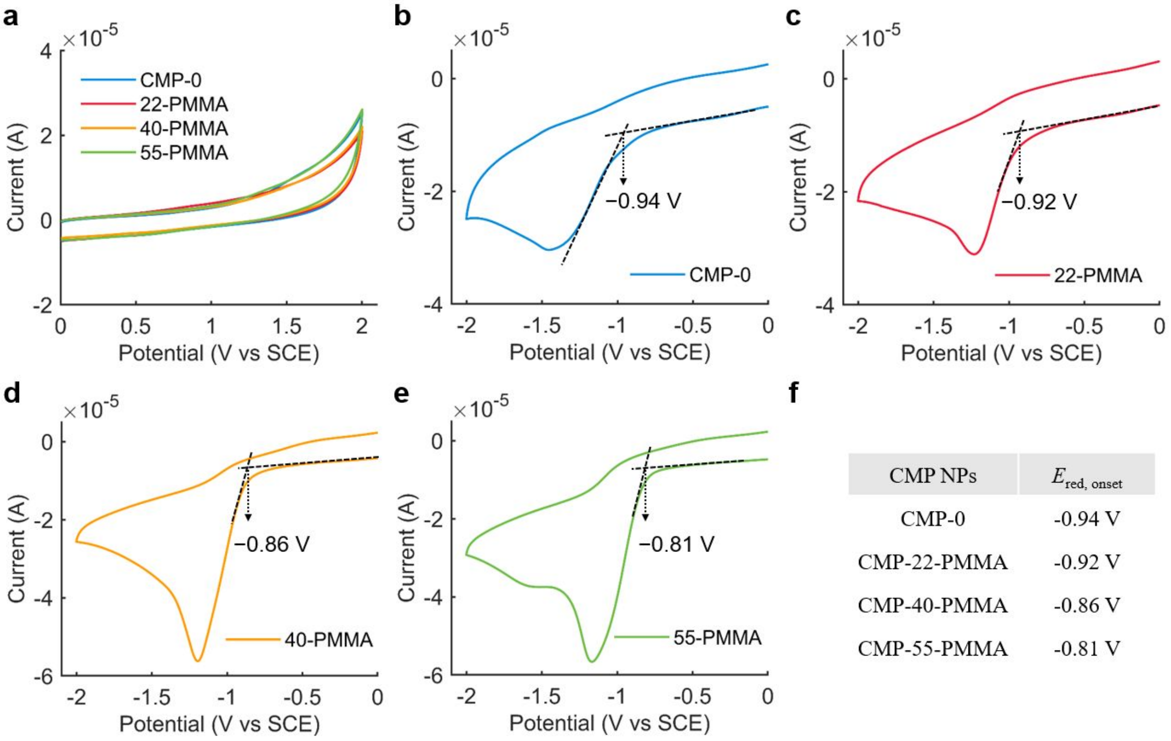

CMP NPs	$E_{red, onset}$
CMP-0	-0.94 V
CMP-22-PMMA	-0.92 V
CMP-40-PMMA	-0.86 V
CMP-55-PMMA	-0.81 V

Figure S12. Cyclic voltammograms of CMP-0 and PMMA-grafted CMP NPs in the (a) oxidation cycles and (b-e) reduction cycles. (a) No oxidation peaks were observed within the potential window (−2.0 V to +2.0 V vs SCE) of 0.1 M NBu_4PF_6 in acetonitrile. (b) CMP-0. (c) CMP-22-PMMA. (d) CMP-40-PMMA. (e) CMP-55-PMMA. (f) Reduction onset potential of CMP-0 and CMP-x-PMMA NPs was used to estimate LUMO levels in the main text (**Figure 2d**). Half-peak reduction potential ($E^{(p/2)}$, V vs SCE) of CMP NPs was also determined as follows: CMP-0 (−1.14), CMP-22-PMMA (−1.07), CMP-40-PMMA (−1.02), and CMP-55-PMMA (−0.96).

a

entry	solvents	density (g cm^{-3})
①	Chloroform	1.49
②	Dichloromethane	1.33
③	1,2-Dichloroethane	1.25
④	Nitromethane	1.14
⑤	Benzofluoride	1.19

b **c**

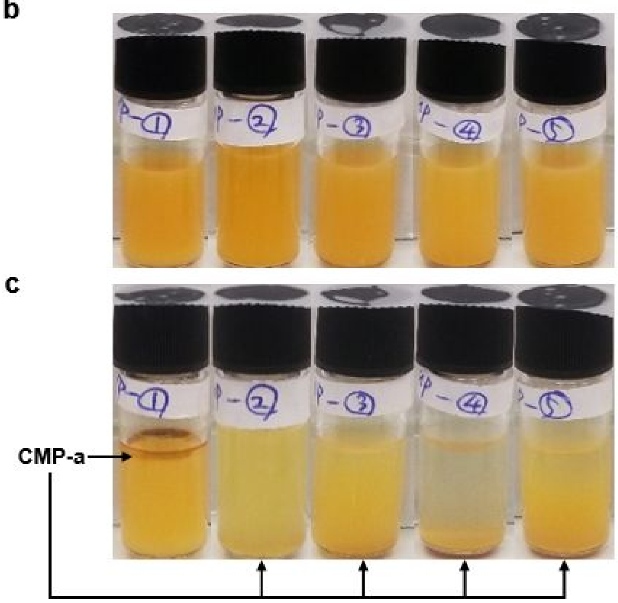

Figure S13. Dispersibility and colloidal stability of CMP-0 NPs in various organic solvents. (a) Organic solvents tested in the experiment. (b) A photo of CMP-0 dispersion in the solvents immediately after 10 min of sonication. CMP-0 can be dispersed in all of the solvents upon sonication. (c) A photo of the CMP-0 dispersion allowed to sit for 12 h without stirring after the sonication. In the solvent ④, nitromethane, most of CMP-0 NPs were deposited on the bottom of the vial, indicating the poorest colloidal stability of CMP-0 in nitromethane among the solvents tested. Additionally, the density of CMP-0 NPs was estimated to be 1.4 g/cm^3 because the CMP-0 aggregates floated in chloroform (d = 1.49 g/cm^3), but sank in other solvents with density below 1.4 g/cm^3.

a

entry	solvents	density (g cm^{-3})
①	Chloroform	1.49
②	Dichloromethane	1.33
③	1,2-Dichloroethane	1.25
④	Nitromethane	1.14
⑤	Benzofluoride	1.19

b

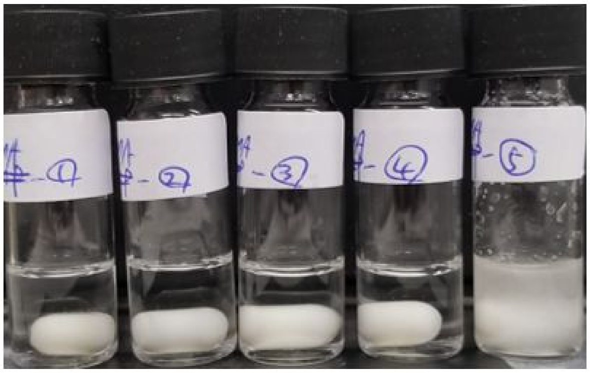

Figure S14. Solubility of PMMA in various organic solvents. (a) Organic solvents tested in the experiment. (b) A photo of 200 mg of PMMA (M_w = 94,600 g/mol, M_n = 52,300 g/mol) in 1 mL of the organic solvents after 45 min of stirring. PMMA is soluble in all the solvents except for the solvent ⑤, benzofluoride.

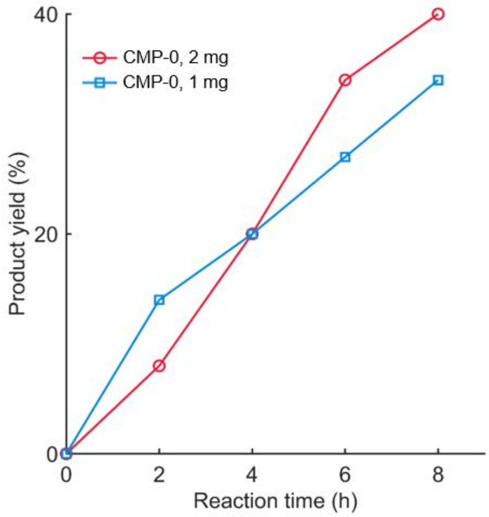

Figure S15. Effect of the amount of CMP-0 in the photocatalytic oxidative [3+2] cycloaddition kinetics. In the beginning (0–2 h), weak stirring caused some CMP-0 at 2 mg/mL precipitating. After fixing this problem for both 1 mg and 2 mg CMP-0 conditions, the rate was faster with 2 mg of CMP-0 than with 1 mg of the photocatalyst. However, the final yield after 20 h was the same as the case with 1 mg CMP-0, and the yield-to-conversion ratio was 0.5. Given that the yield-to-conversion ratio was also around 0.5 even at 52.2% of conversion under reduced light intensity (**Table S6**, entry 5), there could be side reactions in the [3+2] cycloaddition reaction induced by the photoredox catalysis with CMP NPs. Except for the temperature (24 °C), reaction conditions were the same as the standard conditions (**Table S6**). Reaction rates ($M\ s^{-1}$) were calculated using overall yields until 8 h: CMP-0, 2 mg (6.94×10^{7}) and CMP-0, 1 mg (5.90×10^{7}).

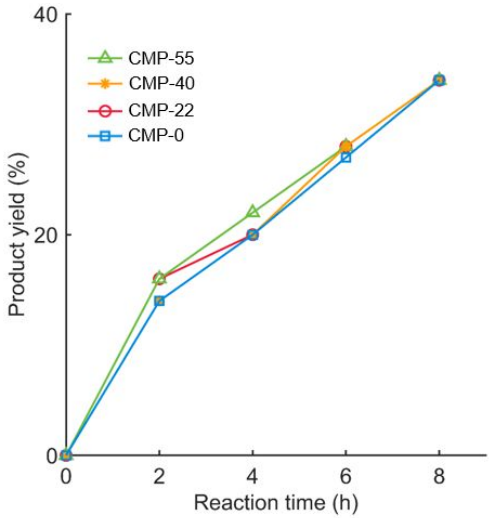

Figure S16. Kinetic profiles of the oxidative [3+2] cycloaddition reaction with CMP-0 and CMP-x NPs. Despite the benzylamine doping, the CMP-x NPs showed similar dispersibility and photocatalytic activity in nitromethane to those of CMP-0. Except for the temperature (24 °C), reaction conditions were the same as the standard conditions (**Table S6**). Reaction rates ($M\ s^{-1}$) were calculated using overall yields until 8 h: CMP-0 (5.90×10^{7}), CMP-22 (5.90×10^{7}), CMP-40 (5.90×10^{7}), and CMP-55 (5.90×10^{7}).

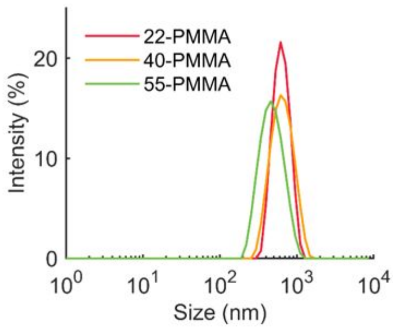

Figure S17. Particle size distribution of CMP-x-PMMA NPs obtained by dynamic light scattering (DLS) measurements. The CMP NPs were dispersed in nitromethane at 0.1 mg/mL by sonication for 10 miniutes before measurements. Mean hydrodynamic diameters were 642.9 nm, 672.5 nm, and 493.6 nm for CMP-22-PMMA, CMP-40-PMMA, and CMP-55-PMMA, respectively.

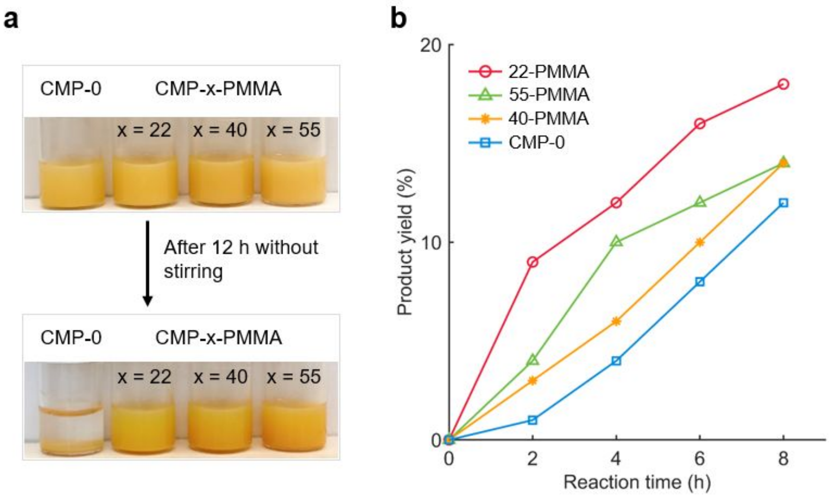

Figure S18. Dispersibility and photocatalytic oxidative [3+2] cycloaddition kinetics of CMP-0 and CMP-x-PMMA NPs under limited stirring conditions. (a) Images of CMP-0 and CMP-x-PMMA NPs dispersion in nitromethane immediately after ten minutes of sonication and after 12 hours without stirring. Obviously, the PMMA grafting stabilizes CMP NPs and prevents them from aggregation and precipitation. (b) Kinetic profiles of the oxidative [3+2] cycloaddition reaction with stirring every two hours before sampling. CMP-22-PMMA achieved 50% faster kinetics than CMP-0 under the same conditions. Reaction rates (M s^{-1}) were calculated using overall yields until 8 h: CMP-22 (3.13×10^{7}), CMP-55 (2.43×10^{7}), CMP-40 (2.43×10^{7}), and CMP-0 (2.08×10^{7}).

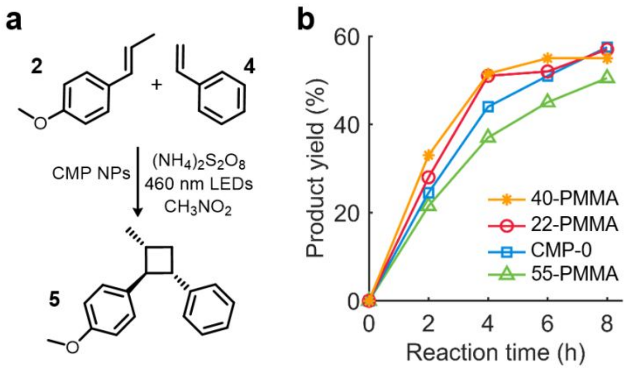

Figure S19. Oxidative [2+2] cycloaddition of **2** to styrene (**4**) with CMP-0 and PMMA-grafted CMP NPs in nitromethane. Reaction scheme (a) and kinetic profiles (b) of the reaction. Reaction rates (M s^{-1}) were calculated using overall yields until 4 h: CMP-40-PMMA (3.58×10^{6}), CMP-22-PMMA (3.54×10^{6}), CMP-0 (3.05×10^{6}), and CMP-55-PMMA (2.57×10^{6}). Given the less positive oxidation potential (E_{ox} = +1.17 V vs SCE)[16] of **2** than the HOMO level of CMP NPs, we supposed that direct photocatalytic oxidation of **2** and subsequent intermolecular cycloaddition would be feasible with CMP NPs. This observation enabled us to focus on developing photocatalytic [2+2] cycloaddition of **2** to styrene (**4**) in nitromethane.[9] In this reaction, 10.0 equiv. of **4** was employed to prevent the [2+2] homocycloaddition of **2** by trapping the photooxidized radical cation ($\mathbf{2}^{\bullet+}$) while cyclodimerization of **4** was expected to be insignificant due to its high oxidation potential (E_{ox} = +2.05 V vs SCE).[17] Together with 1 mg CMP-0 in 1 mL nitromethane, 0.1 mmol (1.0 equiv.) of **2**, and 1.0 equiv. of $(NH_4)_2S_2O_8$ as a terminal oxidant, a high conversion (100 %) and a moderate yield (52.5%) were obtained after 20 h at 18 °C under blue light (11.9 mW cm^{-2}) (**Table S7**, entry 1). Control experiments supported indispensable roles of the photocatalyst, light, and $(NH_4)_2S_2O_8$ in the reaction (entries 2–4). Using oxygen as a terminal oxidant instead of $(NH_4)_2S_2O_8$ was detrimental to the product yield (7.5%). More $(NH_4)_2S_2O_8$ (2.0 equiv.) improved the yield (60%) whereas more CMP-0 (2 mg mL^{-1}) did not increase the yield further (entries 5–6). The kinetic profiles above demonstrated that PMMA-grafting could lead to better dispersions of CMP NPs, and thus, improve the photocatalytic efficiency (CMP-22-PMMA and CMP-40-PMMA) unless the grafting decreased the photocatalyst loading too excessively as in CMP-55-PMMA. Interestingly, unlike in the photocatalytic oxidative [3+2] cycloaddition (**Figures 3a–b**), CMP-40-PMMA showed comparable performance to that of CMP-22-PMMA. Considering the marginal difference in PMMA weight fractions between CMP-22-PMMA (10.6 wt%) and CMP-40-PMMA (10.9 wt%), the photocatalytic efficiency in this reaction seems to depend mainly on the photocatalyst loading and its dispersibility in the reaction mixture.

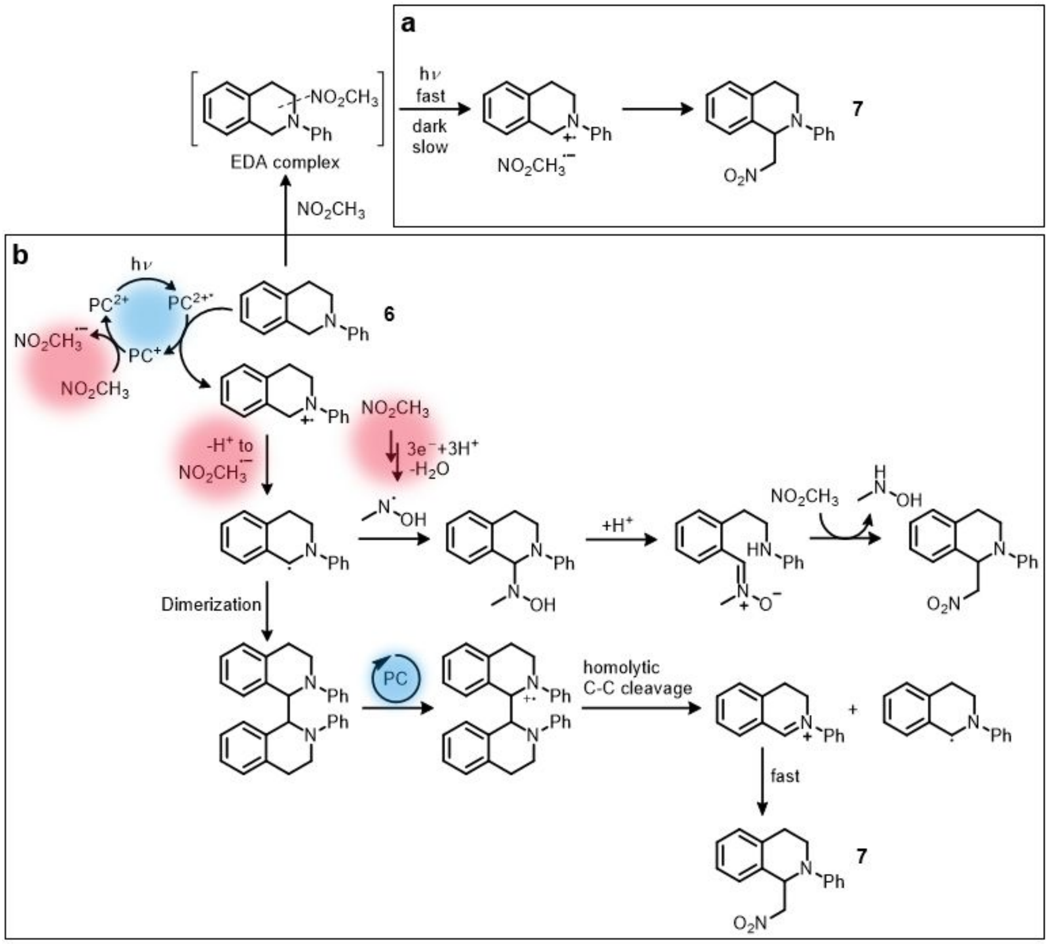

Figure S20. Proposed mechanism in the literature for photocatalyzed aza-Henry reaction in the absence of oxygen.[18] (a) Background reactions through the electron donor-acceptor (EDA) complex between **6** and nitromethane (CH_3NO_2) with or without intense blue light. (b) Photocatalytic aza-Henry reaction via radical pathways. In the reported mechanistic study, $Ru(bpy)_3Cl_2$ was used as the photocatalyst (PC^{2+}) under blue light. The critical roles of CH_3NO_2 as a terminal oxidant and precursor for a base and a radical intermediate are highlighted in red.

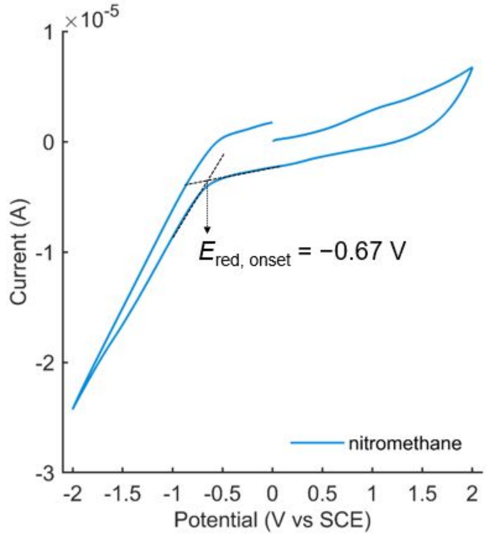

Figure S21. Cyclic voltammogram of nitromethane solvent. Electrochemical reduction of nitromethane occurs under more negative potential than −0.67 V vs SCE, and the higher rates are achieved with more negative potential.

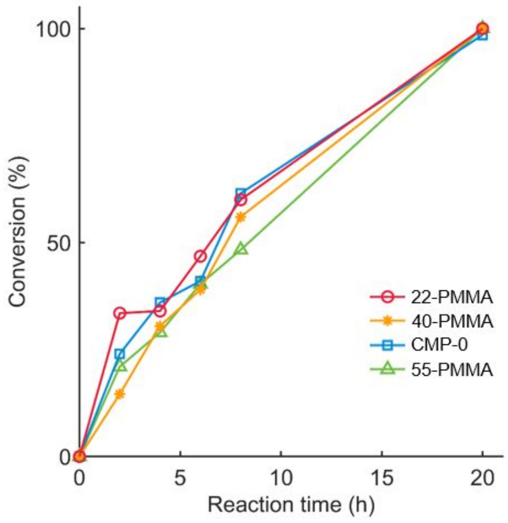

Figure S22. Kinetic profiles of the photocatlyzed aza-Henry reaction in conversion (%).

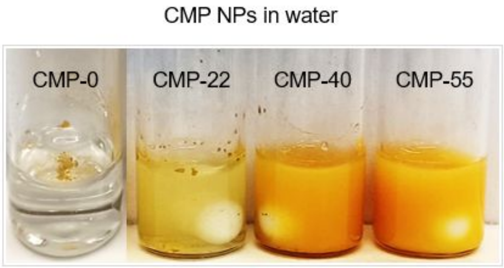

Figure S23. Dispersibility of CMP-0, CMP-22, CMP-40, and CMP-55 in water. The images were taken after sonicating 1 mg/mL CMP-0 or CMP-x NPs in water for 10 minutes. The dispersibility of CMP NPs in water was improved with more benzylammonium groups.

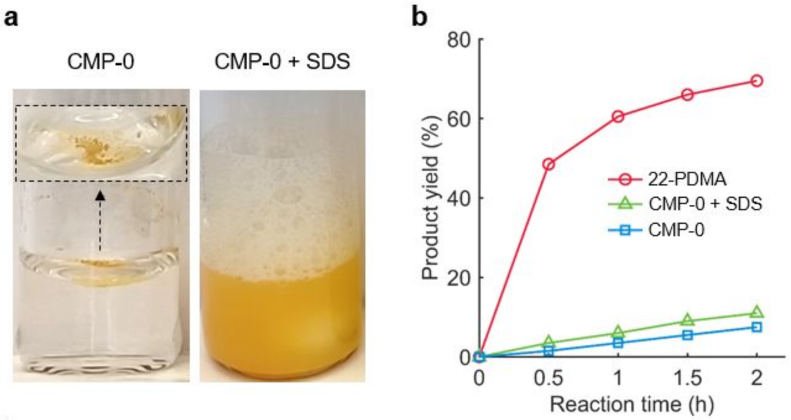

c

CMP NPs	BET surface area (m^2 g^{-1})	pore volume (cm^3 g^{-1})
CMP-0 before sovolysis of SDS	32	0.07
CMP-0	326	0.25

Figure S24. Effect of surfactants, sodium n-dodecyl sulfate (SDS), on dispersibility and thiol-ene reaction kinetics of CMP-0 in phosphate buffer solutions (0.2 M, pH 7.4). (a) Image of CMP-0 (1 mg/mL) in the buffer solutions with or without 10 mM SDS after 10 min of sonication. In the presence of SDS, CMP-0 can be dispersed in the aqueous solutions. (b) Kinetic profiles of the photocatalytic thiol-ene reaction with CMP-0, CMP-0 with SDS, and CMP-22-PDMA. Reaction rates (M s^{-1}) were calculated using overall yields until 2 h: CMP-22-PDMA (9.65×10^{6}), CMP-0 with SDS (1.53×10^{6}), and CMP-0 (1.04×10^{6}). Despite the enhanced dispersibility of CMP-0 in the presence of SDS, the increase in the reaction rate was barely noticeable compared to the case with PDMA grafting. (c) BET analysis data for CMP-0 before and after solvolysis of SDS. BET surface area calculated over the pressure range 0.05–0.28 P/P°. Pore volume calculated at P/P°=0.95. The ten-fold increase in the BET surface area upon the sovolysis of SDS implies that the small molecule surfactant can occupy the interior pores of CMP NPs, reducing the substrate accessibility to the active sites.

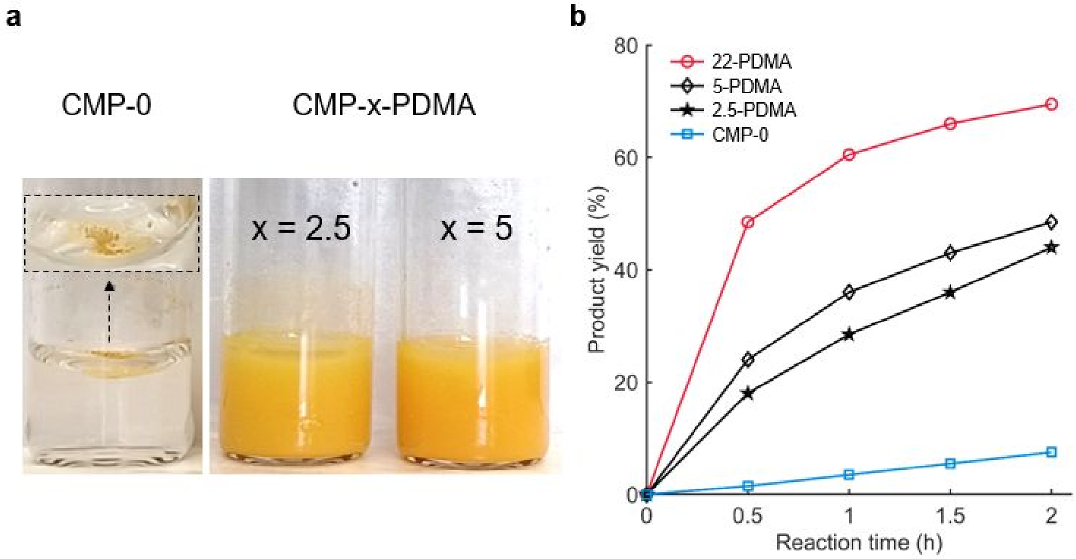

Figure S25. Investigating photocatalytic efficiency of hairy CMP NPs with lower benzylamine content than that of CMP-22-PDMA. The CMP-2.5-PDMA and CMP-5-PDMA were prepared using the same protocol described above except for the molar feed ratios of monomers **B**:**C** = 97.5:2.5 and 95:5. (a) Image of CMP NPs in phosphate buffer solutions after 10 min of sonication. The PDMA-grafting could enhance the dispersibility of CMP NPs even with very low benzylamine content (<5 mol%). (b) Kinetic profiles of the photocatalytic thiol-ene reaction including CMP-2.5-PDMA and CMP-5-PDMA as photocatalysts. Reaction rates ($M\ s^{-1}$) were calculated using overall yields until 2 h: CMP-22-PDMA (9.65×10^{6}), CMP-5-PDMA (6.74×10^{6}), CMP-2.5-PDMA (6.11×10^{6}), and CMP-0 (1.04×10^{6}). In this regime of low benzylamine content (< 22 mol%), the hairy CMP NPs with more benzylamine content or higher surface grafting density showed higher photocatalytic efficiency.

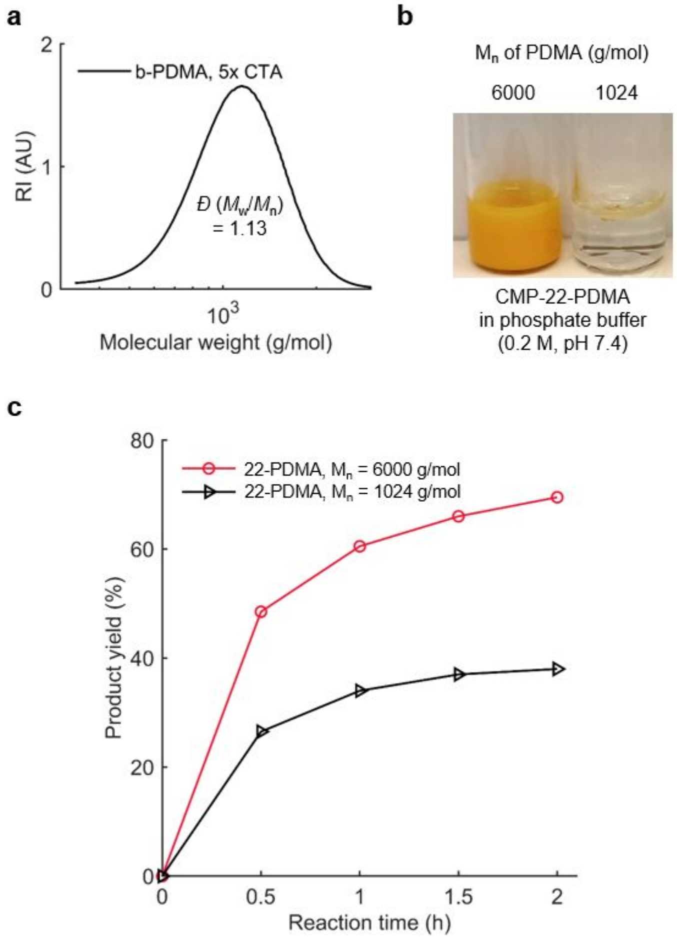

Figure S26. Chain length effect in CMP-22-PDMA NPs. (a) Molecular weight distribution curves of free polymers generated during the synthesis of CMP-22-PDMA using the standard PDMA grafting protocol except for five times the amount of CTA. The M_n of PDMA chain is 1024 g/mol. (b) Image of CMP-22-PDMA NPs in phosphate buffer solutions (1 mg/mL) after 10 min of sonication. Obviously, the CMP-22-PDMA with shorter chain showed limited dispersibility. (c) Kinetic profiles of the thiol-ene reaction with both CMP-22-PDMA NPs. Reaction rates (M s^{-1}) were calculated using overall yields until 2 h: CMP-22-PDMA, M_n = 6000 g/mol (9.65×10^{6}) and CMP-22-PDMA, M_n = 1024 g/mol (5.28×10^{6}). The addition of the substrates (**8** and **9**) to the system could slightly enhance the dispersibility of CMP-22-PDMA with short chain, but the kinetics was much slower than the CMP-22-PDMA with long chain.

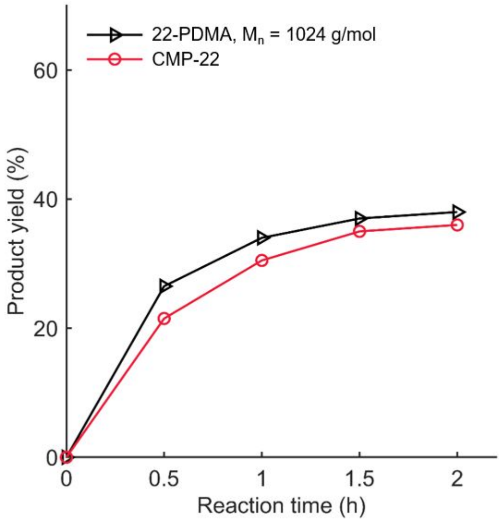

Figure S27. Kinetic profiles of the photocatalytic thiol-ene reaction with short chain-grafted CMP-22-PDMA and CMP-22. Reaction rates (M s^{-1}) were calculated using overall yields until 2 h: CMP-22-PDMA, M_n = 1024 g/mol (5.28×10^{6}) and CMP-22 (5.00×10^{6}).

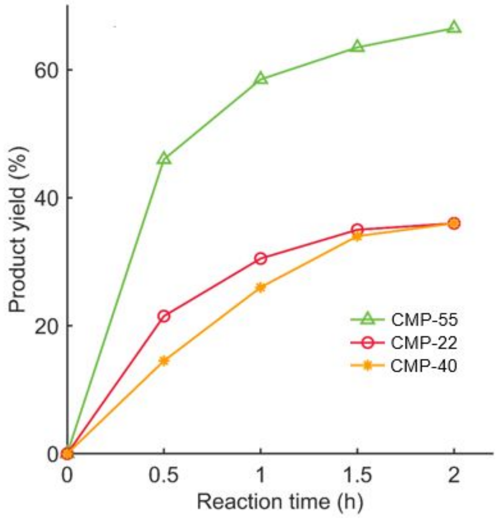

Figure S28. Kinetic profiles of the photocatalytic thiol-ene reaction with CMP-x NPs. Reaction rates (M s^{-1}) were calculated using overall yields until 2 h: CMP-55 (9.24×10^{6}), CMP-22 (5.00×10^{6}), and CMP-40 (5.00×10^{6}).

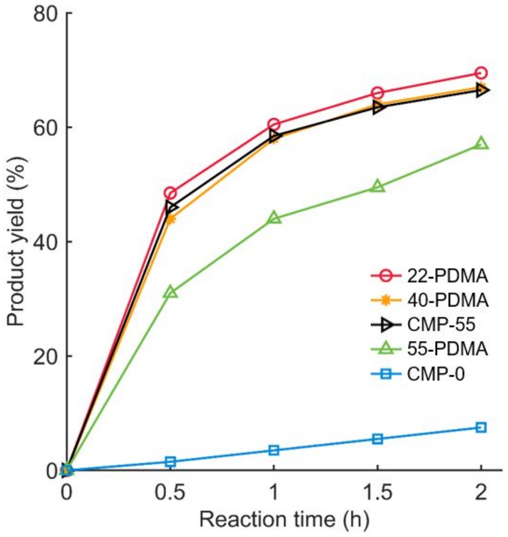

Figure S29. Kinetic profiles of the photocatalytic thiol-ene reaction including CMP-55 as a photocatalyst. Reaction rates (M s^{-1}) were calculated using overall yields until 2 h: CMP-22-PDMA (9.65×10^{6}), CMP-40-PDMA (9.31×10^{6}), CMP-55 (9.24×10^{6}), CMP-55-PDMA (7.92×10^{6}), and CMP-0 (1.04×10^{6}). The CMP-55 photocatalyst showed comparable photocatalytic thiol-ene reaction kinetics to those of CMP-22-PDMA and CMP-40-PDMA. Considering that the hairy CMP NPs were used with less photocatalyst loadings due to the grafted polymers, 11.9 wt% and 12.5 wt%, respectively, the enhanced dispersibility by PDMA further improved the substrate accessibility to active sites of the photocatalysts compared to CMP-55. However, excessive polymer grafting (14.8 wt% PDMA in CMP-55-PDMA) decreased the photocatalytic efficiency likely due to much less photocatalyst loading and hindered substrate access to the active sites of CMP NPs.

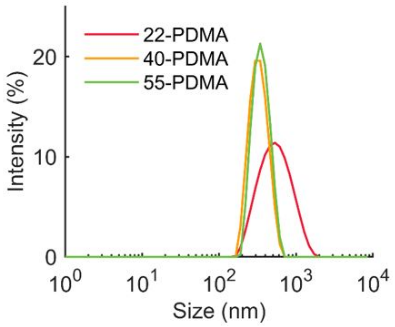

Figure S30. Particle size distribution of CMP-x-PDMA NPs obtained by DLS measurements. The CMP NPs were dispersed in phosphate buffer solutions (0.2 M, pH 7.4) at 0.1 mg/mL by sonication for 10 minutes before measurements. Mean hydrodynamic diameters were 587.5 nm, 336.4 nm, and 359.9 nm for CMP-22-PDMA, CMP-40-PDMA, and CMP-55-PDMA, respectively.

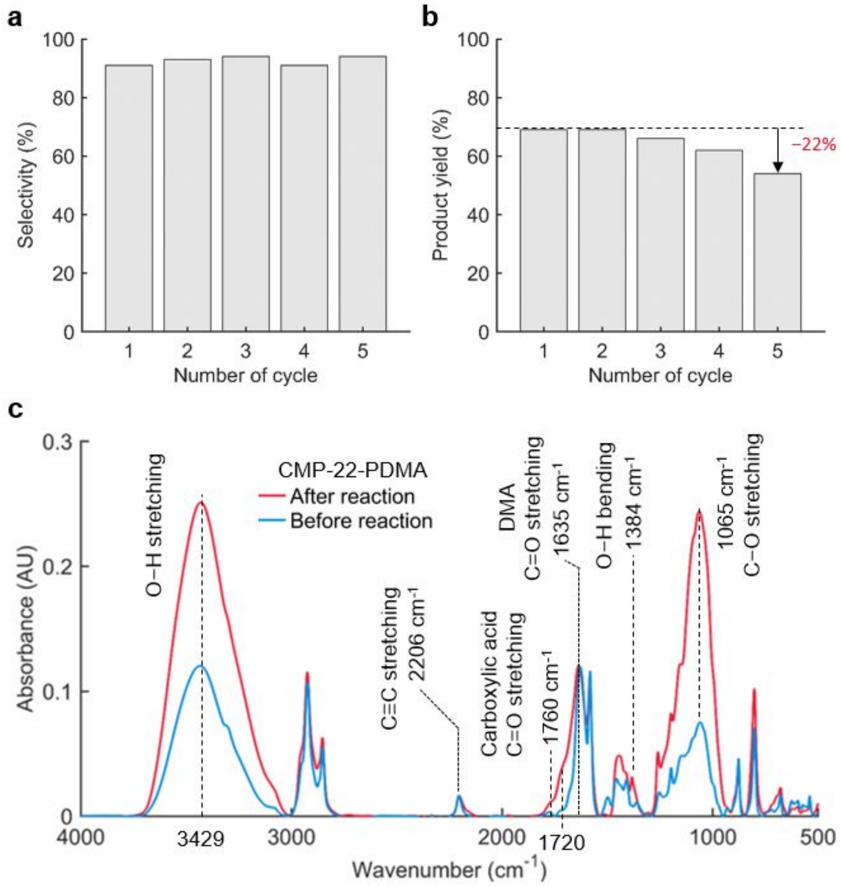

Figure S31. Recyclability tests of CMP-22-PDMA in the photocatalytic thiol-ene reaction. (a) Selectivity of repeated reactions. (b) Product yields of repeated reactions. (c) FT-IR spectra of CMP-22-PDMA before and after five cycles of the thiol-ene reactions. The results show that the product yield decreased 22% in the 5th cycle of the reaction compared to the first cycle. Given that the 3.3 wt% less photocatalyst loading (CMP-22-PDMA vs CMP-55-PDMA) yielded the 18% less products after two hours of the reaction as described in **Figure S29**, slight degradation or loss (< 5%) of the photocatalyst during the recyclability tests may account for the decrease in the photocatalytic efficiency. The FT-IR spectra of CMP-22-PDMA before and after the recyclability tests demonstrate that there was significant increase in O–H stretching (3249 cm^{-1}), O–H bending (1384 cm^{-1}), C–O stretching (1065 cm^{-1}), and carboxylic acid C=O stretching (1760 cm^{-1} and 1720 cm^{-1}) signals after the reactions while FT-IR signals for the CMP backbone structure and the grafted PDMA remained unchanged. This observation suggests that the change in the FT-IR spectra is mainly because of the entrapment of reactants and products (**8**, **9**, and **10**) in the CMP NPs, not because of the degradation of CMP-22-PDMA during the reaction. In fact, the weight of the recovered photocatalyst

after the 5th cycle was slightly higher (1.03 mg) than initial one (1.00 mg) despite apparent adsorption loss of the photocatalyst to tube walls, which also supports the interpretation.

Ⅳ. Supplementary Tables

Table S1. Total mole numbers (in mmol) of functional groups in each monomer used in the synthesis of CMP NPs via Sonogashira cross-coupling.

CMP NPs	total mole of terminal alkyne in **A**	total mole of aryl bromide in **B**	total mole of aryl bromide in **C**	total mole of aryl bromide in **B** + **C**	molar feed ratio **A** : **B** : **C**
CMP-0	0.999	1.04	0.00	1.04	2.5 : 4.0 : 0
CMP-22	0.999	0.91	0.13	1.04	2.5 : 3.5 : 1
CMP-40	0.999	0.78	0.26	1.04	2.5 : 3.0 : 2
CMP-55	0.999	0.65	0.39	1.04	2.5 : 2.5 : 3

Table S2. BET analysis data for CMP NPs.

CMP NPs	BET surface area[a] (m^2 g^{-1})	pore volume[b] (cm^3 g^{-1})
CMP-0	326	0.25
CMP-22	388	0.30
CMP-40	392	0.31
CMP-55	337	0.26

[a] BET surface area calculated over the pressure range 0.05–0.28 P/P°.

[b] Pore volume calculated at P/P°=0.95.

Table S3. *t*-plot analysis data for CMP NPs.

CMP NPs	micropore surface area[a] (m^2 g^{-1})	mesopore surface area[a] (m^2 g^{-1})	micropore volume[a] (cm^3 g^{-1})
CMP-0	138 (42%)[b]	188 (58%)[c]	0.071
CMP-22	179 (46%)[b]	209 (54%)[c]	0.090
CMP-40	181 (46%)[b]	211 (54%)[c]	0.091
CMP-55	192 (57%)[b]	145 (43%)[c]	0.097

[a] values calculated over the pressure range 0.05–0.70 P/P°.

[b] values in parentheses indicate the micropore contribution to total BET surface area.

[c] values in parentheses indicate the mesopore contribution to total BET surface area. The substantial mesopore contribution could be due to incomplete Sonogashira cross-coupling reactions in miniemlusions as described elsewhere.[19]

Table S4. Molar feed ratios of monomers and molar ratios of monomers incorporated into CMP NPs.

CMP NPs	molar feed ratios **A** : **B** : **C**	corrected molar feed ratios of **B** : **C**[a]	molar ratios of **B** : **C** from solid-state ^{1}H NMR[b]	benzylamine doping (%)[c]
CMP-0	2.5 : 4.0 : 0	-	-	-
CMP-22	2.5 : 3.5 : 1	3.5 : 0.8	3.5 : 0.9	20.5
CMP-40	2.5 : 3.0 : 2	3.0 : 1.2	3.0 : 1.3	30.2
CMP-55	2.5 : 2.5 : 3	2.5 : 1.3	2.5 : 1.3	34.2

[a] values calculated by assuming the similar reactivity of **B** and **C** towards **A** and excluding oligomers. See **Figure S3** for details.

[b] values calculated from integration ratios of aromatic protons and benzylamine CH_2–N protons. See **Figure S3** for details. The ratios of **B** : **C** integrated into the CMP network are very close to the corrected molar feed ratios, implying that most of oligomers were removed from the CMP NPs during purification steps.

[c] values calculated from the ratios of **B** : **C** in the CMP NPs.

Table S5. GPC results and surface grafting density of grafted polymers.

CMP NPs	M_n (g mol^{-1})	$Đ$ (M_w/M_n)	surface grafting density (chains nm^{-2})[a]
CMP-22-PMMA	31300	1.24	0.083
CMP-40-PMMA	26400	1.52	0.099
CMP-55-PMMA	28100	1.38	0.121
CMP-22-PDMA	6000	1.56	0.493
CMP-40-PDMA	4800	1.52	0.633
CMP-55-PDMA	4800	1.63	0.808

[a] Details for the calculation is available in the Methods section. The surface grafting density of polymers are smaller than the surface density of CTA in CMP-x-CTA NPs (**Figure S5**).

Table S6. Control and optimization experiments of the photocatalytic oxidative [3+2] cycloaddition with CMP-0 as a photocatalyst[a]

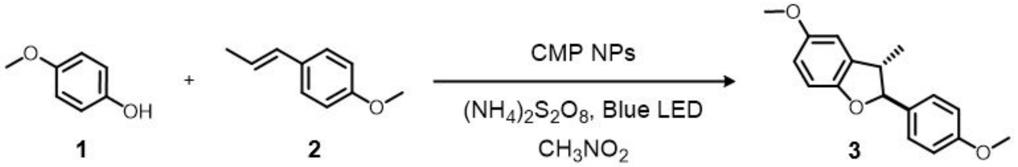

entry	changes to standard conditions	conversion (%)[b]	yield (%)[b]
1	none	94.2	50
2	no light	13	1
3	no CMP-0	22	2
4	2 mg/mL CMP-0	100	47
5	decreased light intensity to 5.4 mW/cm^2	52.2	26
6	increased light intensity to 17 mW/cm^2	95	49
7	increased light intensity to 17 mW/cm^2 and 4.0 equiv. of $(NH_4)_2S_2O_8$	96.5	49

[a] Standard conditions: 1 mg CMP-0 in 1 mL nitromethane, 0.05 mmol 4-methoxyphenol (**1**, 1.0 equiv.), 0.075 mmol *trans*-anethole (**2**, 1.5 equiv.), 0.1 mmol $(NH_4)_2S_2O_8$ (2.0 equiv.), blue LED (λ_{max} = 460 nm) at 11.9 mW/cm^2, 20 h, under N_2 environment, Temperature = 18 °C.

[b] Conversions and yields determined by ^1H NMR spectroscopy using 1,3-dimethoxybenzene as an internal standard.

Table S7. Control and optimization experiments of the photocatalytic oxidative [2+2] cycloaddition with CMP-0 as a photocatalyst[a]

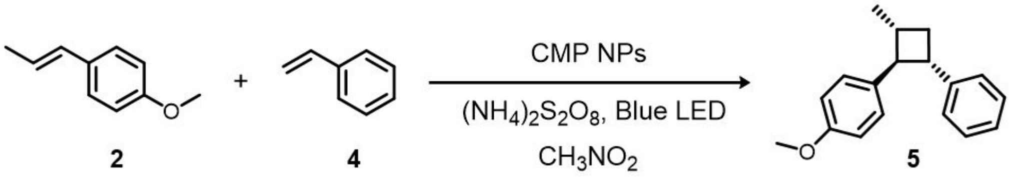

entry	changes to standard conditions	conversion (%)[b]	yield (%)[b]
1	none	100	52.5
2	no light	33.5	3.5
3	no CMP-0	56	5
4	open to air and no $(NH_4)_2S_2O_8$	90	7.5
5	2.0 equiv. $(NH_4)_2S_2O_8$	100	60
6	2.0 equiv. $(NH_4)_2S_2O_8$ and CMP-0 2 mg/mL	100	60

[a] Standard conditions: 1 mg CMP-0 in 1 mL nitromethane, 0.1 mmol *trans*-anethole (**2**, 1.0 equiv.), 1 mmol styrene (**4**, 10.0 equiv.), 0.1 mmol $(NH_4)_2S_2O_8$ (1.0 equiv.), blue LED (λ_{max} = 460 nm) at 11.9 mW/cm^2, 20 h, under N_2 environment, Temperature = 18 °C.

[b] Conversions and yields determined by ^{1}H NMR spectroscopy using 1,3-dimethoxybenzene as an internal standard.

Table S8. Control and optimization experiments of the photocatalyzed aza-Henry reaction with CMP-0 as a photocatalyst[a]

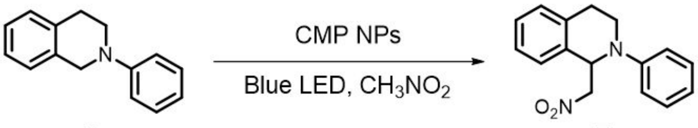

entry	changes to standard conditions	conversion (%)[b]	yield (%)[b]
1	none	100	47
2	no light	21.3	0
3	no CMP-0	64.3	23
4	decreased light intensity to 5.4 mW/cm^2	100	55.5
5	decreased light intensity to 5.4 mW/cm^2 and no CMP-0	29.3	12
6	decreased light intensity to 5.4 mW/cm^2 and 2 mg/mL CMP-0	100	55

[a] Standard conditions: 1 mg CMP-0 in 1 mL nitromethane, 0.05 mmol *N*-phenyltetrahydroisoquinoline (**6**, 1.0 equiv.), blue LED (λ_{max} = 460 nm) at 11.9 mW/cm^2, 20 h, under N_2 environment, Temperature = 18 °C.
[b] Conversions and yields determined by ^{1}H NMR spectroscopy using 1,3-dimethoxybenzene as an internal standard.

Table S9. Control and optimization experiments of the photocatalytic thiol-ene reaction with CMP-55 as a photocatalyst[a]

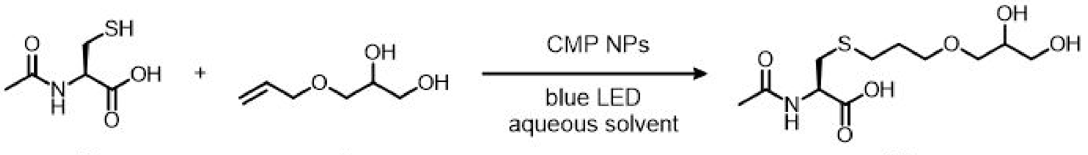

entry	CMP-55	atmosphere	aqueous solvent	reaction time	light intensity	conversion (%)[b]	yield (%)[b]	pH
1	O	N_2	phosphate buffer (pH 7.4, 0.2 M)	2 h	11.9 mW/cm^2	82.5	79	6.32
2	O	N_2	phosphate buffer (pH 7.4, 0.2 M)	2 h	0 mW/cm^2	6.5	0	6.36
3	X	N_2	phosphate buffer (pH 7.4, 0.2 M)	2 h	11.9 mW/cm^2	6	0.5	6.38
4	O	air	phosphate buffer (pH 7.4, 0.2 M)	2 h	11.9 mW/cm^2	50	9.5	6.31
5	O	air	phosphate buffer (pH 7.4, 0.2 M)	2 h	0 mW/cm^2	8.5	0	6.38
6	X	air	phosphate buffer (pH 7.4, 0.2 M)	2 h	11.9 mW/cm^2	4	0	6.36
7	O	N_2	phosphate buffer (pH 7, 0.1 M)	13 h	11.9 mW/cm^2	91	66	3.32
8	O	N_2	phosphate buffer (pH 7, 0.1 M)	13 h	0 mW/cm^2	7.5	0	3.37
9	X	N_2	phosphate buffer (pH 7, 0.1 M)	13 h	11.9 mW/cm^2	30.5	23	3.39
10	O	N_2	DI water	2 h	11.9 mW/cm^2	43	37.5	2.20
11	O	N_2	DI water	2 h	0 mW/cm^2	12	7	2.20
12	X	N_2	DI water	2 h	11.9 mW/cm^2	61.5	57	2.22

[a] Standard conditions: 1 mg CMP-55 in 1 mL aqueous solutions, 0.1 mmol *N*-acetyl-*L*-cysteine (**8**, 1.0 equiv.), 0.2 mmol 3-allyloxy-1,2-propanediol (**9**, 2.0 equiv.), blue LED (λ_{max} = 460 nm) at 11.9 mW/cm^2, 2 h, under N_2 environment, Temperature = 18 °C. Interestingly, a solvent switch to less concentrated phosphate buffer (pH 7, 0.1 M, and final pH of 3.3) not only suppressed the product formation, but also caused significant background reactions (23% yield after 13 h) despite the absence of CMP-55 (entries 7–9). The reaction in deionized water at final pH of 2.2 provided a higher background reaction yield (57%) in

two hours (entries 10–12), confirming the background reaction at low pH and highlighting the importance of controlling pH of the reaction medium.

[b] Conversions and yields determined by ^{1}H NMR spectroscopy using dimethyl sulfone as an internal standard.

Table S10. Selectivity of the photocatalytic thiol-ene reaction with various CMP NPs.

CMP NPs	Selectivity (%)[a]
CMP-0	44.1
CMP-22-PDMA	96.5
CMP-40-PDMA	93.7
CMP-55-PDMA	93.4
CMP-55	91.1

[a] Selectivity was calculated from the conversion and product yield of each reaction after 2 h in the kinetic monitoring experiments (**Figure S29**).

V. References

(1) Ma, B. C.; Ghasimi, S.; Landfester, K.; Zhang, K. A. I. Enhanced Visible Light Promoted Antibacterial Efficiency of Conjugated Microporous Polymer Nanoparticles via Molecular Doping. *J. Mater. Chem. B* **2016**, *4*, 5112–5118.

(2) Han, G.; Tamaki, M.; Hruby, V. J. Fast , Effcient and Selective Deprotection of the Tert-Butoxycarbonyl (Boc) Group Using HCl/Dioxane (4 M). *J. Pept. Res.* **2001**, *58*, 338–341.

(3) Burstein, S.; Lieberman, S. Kinetics and Mechanism of Solvolysis of Steroid Hydrogen Sulfates. *J. Am. Chem. Soc.* **1958**, *80*, 5235–5239.

(4) Dunetz, J. R.; Magano, J.; Weisenburger, G. A. Large-Scale Applications of Amide Coupling Reagents for the Synthesis of Pharmaceuticals. *Org. Process Res. Dev.* **2016**, *20*, 140–177.

(5) Chancellor, A. J.; Seymour, B. T.; Zhao, B. Characterizing Polymer-Grafted Nanoparticles: From Basic Defining Parameters to Behavior in Solvents and Self-Assembled Structures. *Anal. Chem.* **2019**, *91*, 6391–6402.

(6) Blum, T. R.; Zhu, Y.; Nordeen, S. A.; Yoon, T. P. Photocatalytic Synthesis of Dihydrobenzofurans by Oxidative [3+2] Cycloaddition of Phenols. *Angew. Chemie Int. Ed.* **2014**, *53*, 11056–11059.

(7) Huang, W.; Huber, N.; Jiang, S.; Landfester, K.; Zhang, K. A. I. Covalent Triazine Framework Nanoparticles via Size-Controllable Confinement Synthesis for Enhanced Visible-Light Photoredox Catalysis. *Angew. Chemie Int. Ed.* **2020**, *59*, 18368–18373.

(8) Ischay, M. A.; Ament, M. S.; Yoon, T. P. Crossed Intermolecular [2+2] Cycloaddition of Styrenes by Visible Light Photocatalysis. *Chem. Sci.* **2012**, *3*, 2807–2811.

(9) Li, R.; Ma, B. C.; Huang, W.; Wang, L.; Wang, D.; Lu, H.; Landfester, K.; Zhang, K. A. I. Photocatalytic Regioselective and Stereoselective [2+2] Cycloaddition of Styrene Derivatives Using a Heterogeneous Organic Photocatalyst. *ACS Catal.* **2017**, *7*, 3097–3101.

(10) Hari, D. P.; König, B. Eosin Y Catalyzed Visible Light Oxidative C-C and C-P Bond Formation. *Org. Lett.* **2011**, *13*, 3852–3855.

(11) Jiang, J.-X.; Li, Y.; Wu, X.; Xiao, J.; Adams, D. J.; Cooper, A. I. Conjugated Microporous Polymers with Rose Bengal Dye for Highly Efficient Heterogeneous Organo-Photocatalysis. *Macromolecules* **2013**, *46*, 8779–8783.

(12) Cao, X. T.; Bach, L. G.; Islam, M. R.; Lim, K. T. A Simple Synthesis, Characterization, and Properties of Poly(Methyl Methacrylate) Grafted CdTe Nanocrystals. *Mol. Cryst. Liq. Cryst.* **2015**, *618*, 111–119.

(13) Nikolaidis, A. K.; Achilias, D. S. Thermal Degradation Kinetics and Viscoelastic Behavior of Poly(Methyl Methacrylate)/Organomodified Montmorillonite Nanocomposites Prepared via In Situ Bulk Radical Polymerization. *Polymers (Basel).* **2018**, *10*, 491.

(14) Bauri, K.; Roy, S. G.; Arora, S.; Dey, R. K.; Goswami, A.; Madras, G.; De, P. Thermal Degradation Kinetics of Thermoresponsive Poly(N- Isopropylacrylamide-Co-N,N-Dimethylacrylamide) Copolymers Prepared via RAFT Polymerization. *J. Therm. Anal. Calorim.* **2013**, *111*, 753–761.

(15) Makuła, P.; Pacia, M.; Macyk, W. How To Correctly Determine the Band Gap Energy of Modified Semiconductor Photocatalysts Based on UV-Vis Spectra. *J. Phys. Chem. Lett.* **2018**, *9*, 6814–6817.

(16) Yueh, W.; Bauld, N. L. Mechanistic Aspects of Aminium Salt-Catalyzed Diels-Alder Reactions: The Substrate Ionization Step. *J. Phys. Org. Chem.* **1996**, *9*, 529–538.

(17) Schepp, N. P.; Johnston, L. J. Reactivity of Radical Cations. Effect of Radical Cation and Alkene Structure on the Absolute Rate Constants of Radical Cation Mediated Cycloaddition Reactions. *J. Am. Chem. Soc.* **1996**, *118*, 2872–2881.

(18) Bartling, H.; Eisenhofer, A.; König, B.; Gschwind, R. M. The Photocatalyzed Aza-Henry Reaction of N-Aryltetrahydroisoquinolines: Comprehensive Mechanism, $H^{\bullet}$- versus H^{+}-Abstraction, and Background Reactions. *J. Am. Chem. Soc.* **2016**, *138*, 11860–11871.

(19) Zhang, P.; Weng, Z.; Guo, J.; Wang, C. Solution-Dispersible, Colloidal, Conjugated Porous Polymer Networks with Entrapped Palladium Nanocrystals for Heterogeneous Catalysis of the Suzuki-Miyaura Coupling Reaction. *Chem. Mater.* **2011**, *23*, 5243–5249.

Appendix: ^{1}H and ^{13}C NMR spectra

^{1}H NMR spectrum (400 MHz, $CDCl_3$) of **3**.

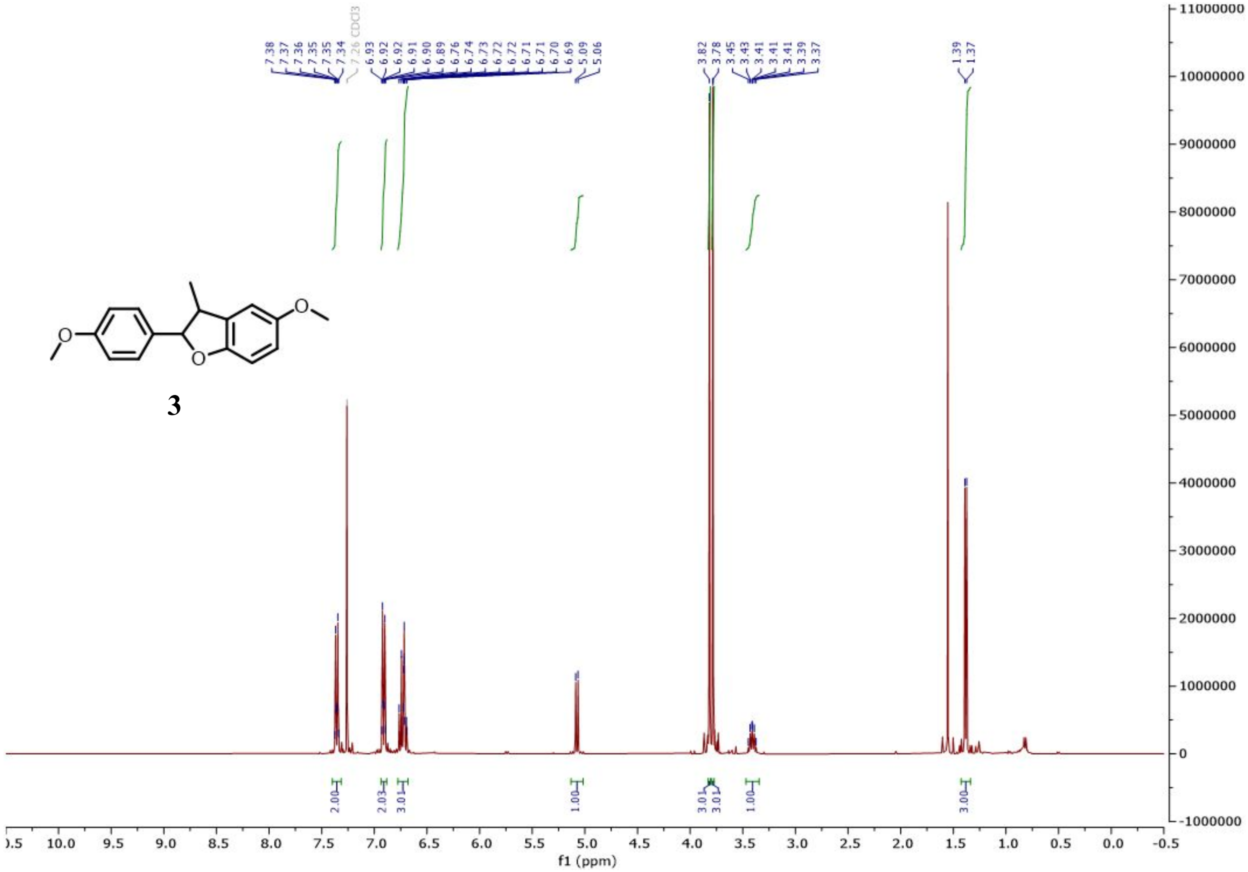

^{13}C-{^{1}H} NMR spectrum (101 MHz, $CDCl_3$) of **3**.

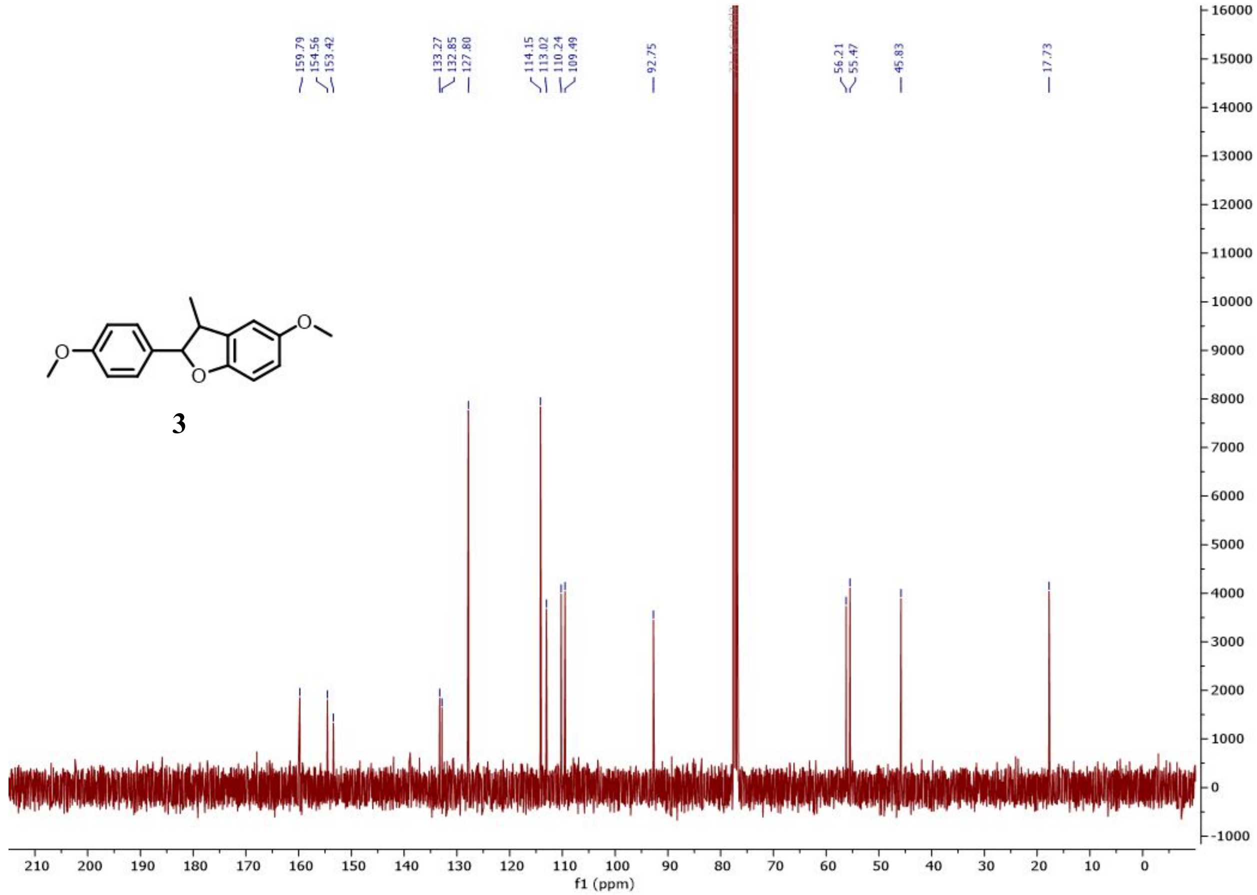

^{1}H NMR spectrum (400 MHz, $CDCl_3$) of **5**.

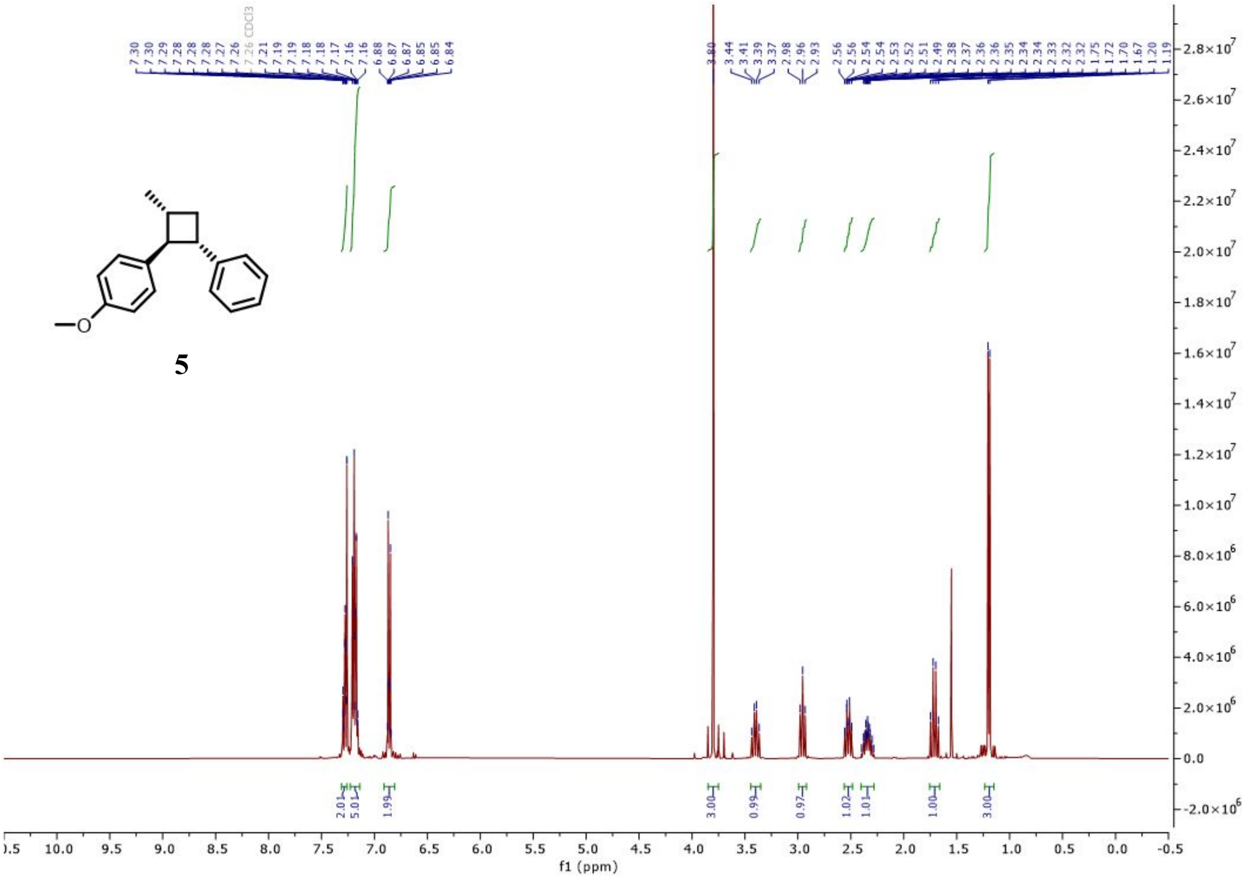

^{13}C-{^{1}H} NMR spectrum (101 MHz, $CDCl_3$) of **5**.

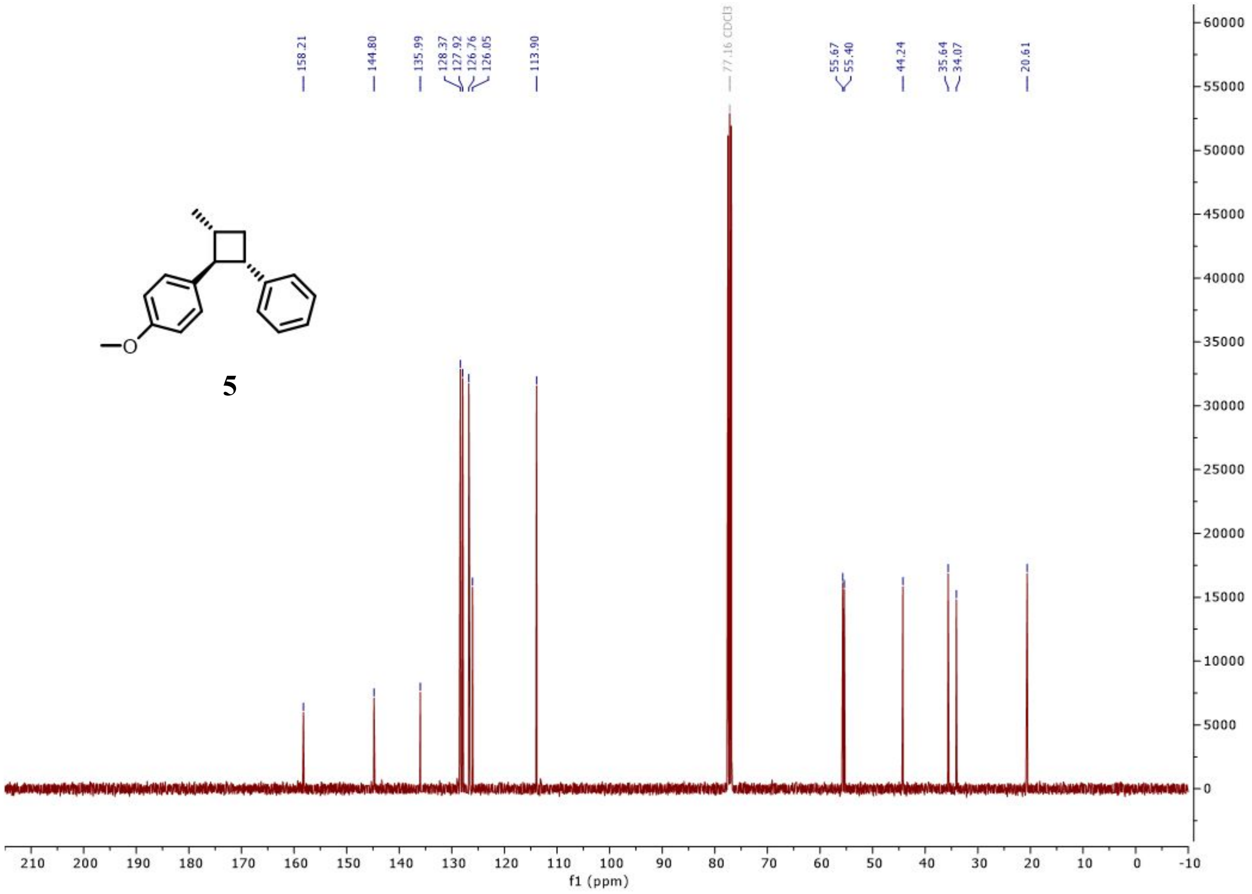

^{1}H NMR spectrum (400 MHz, $CDCl_3$) of **7**.

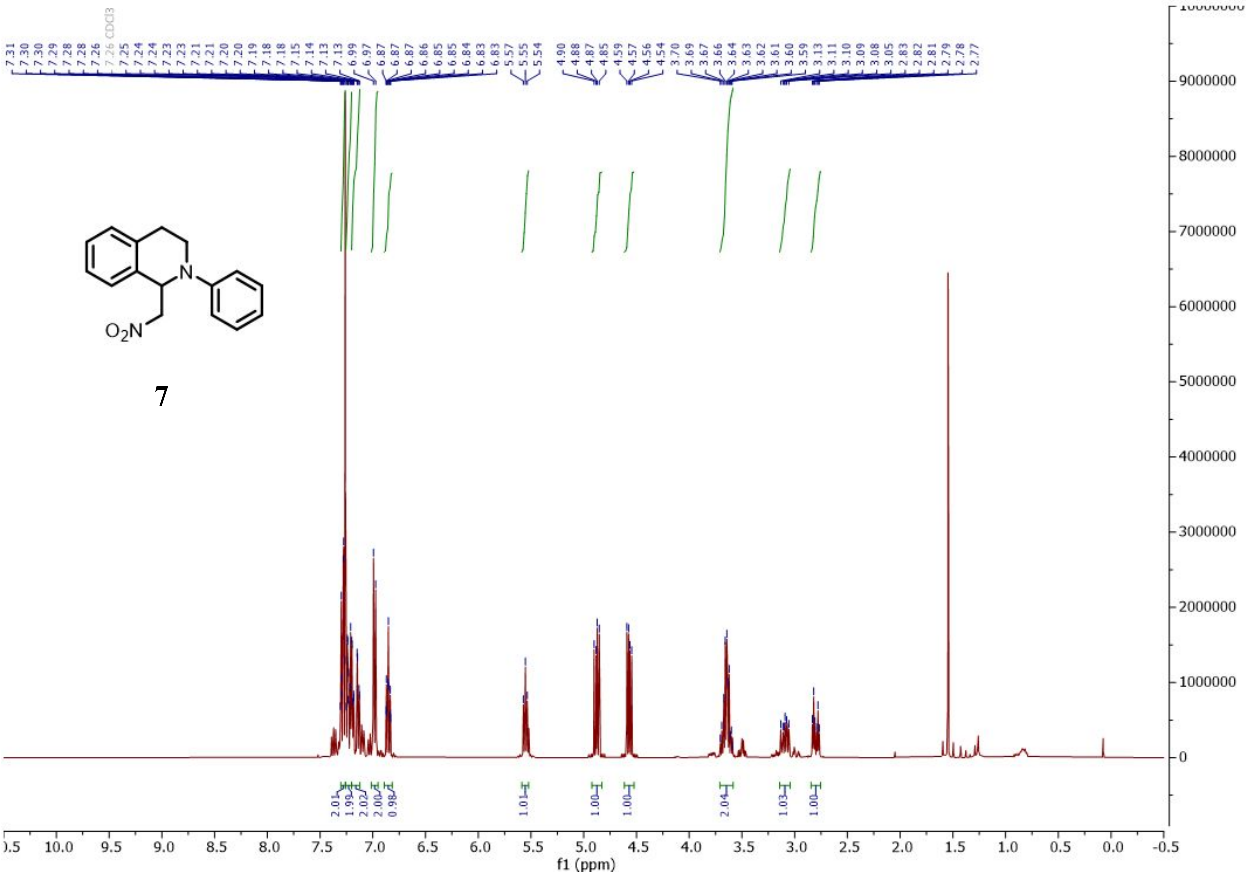

^{13}C-{^{1}H} NMR spectrum (101 MHz, $CDCl_3$) of **7**.

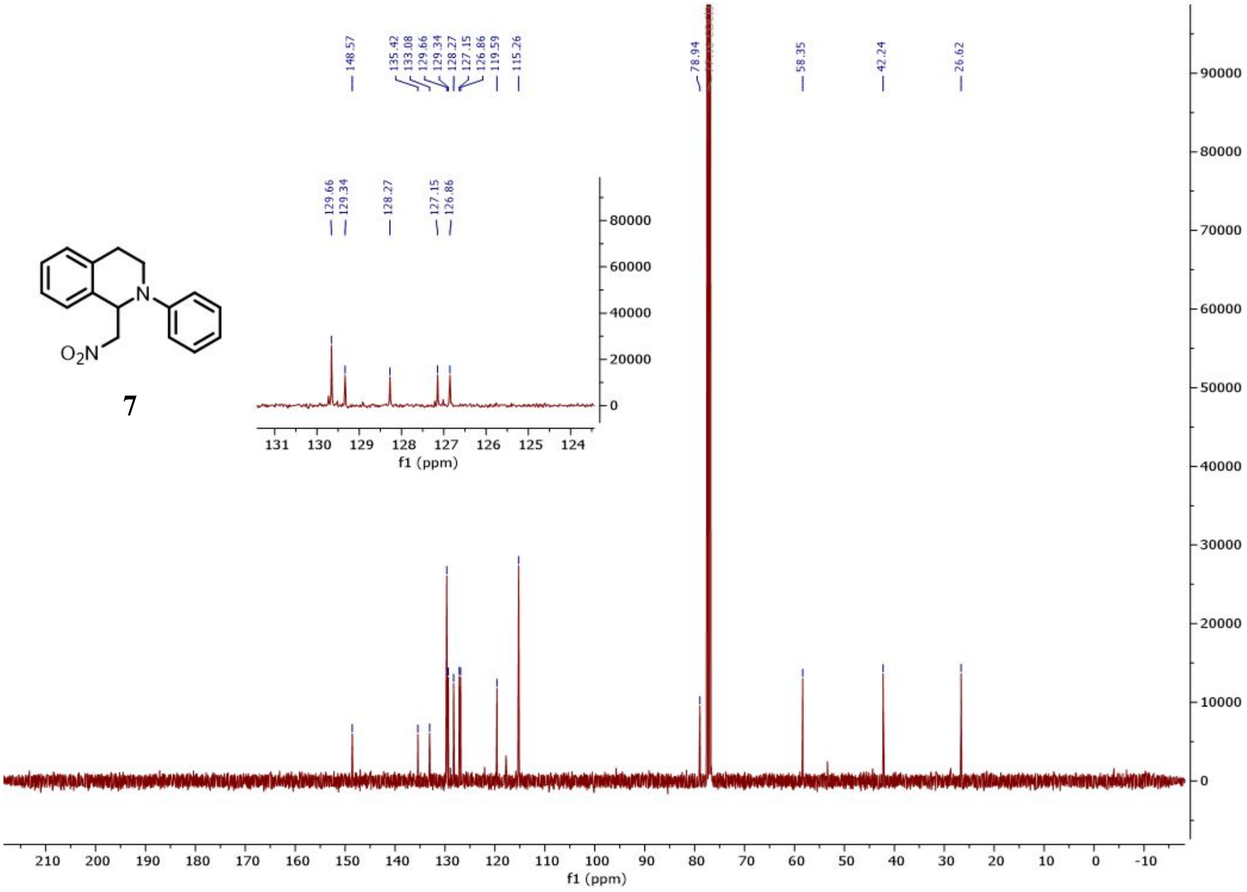

^{1}H NMR spectrum (400 MHz, D_2O) of **10**.

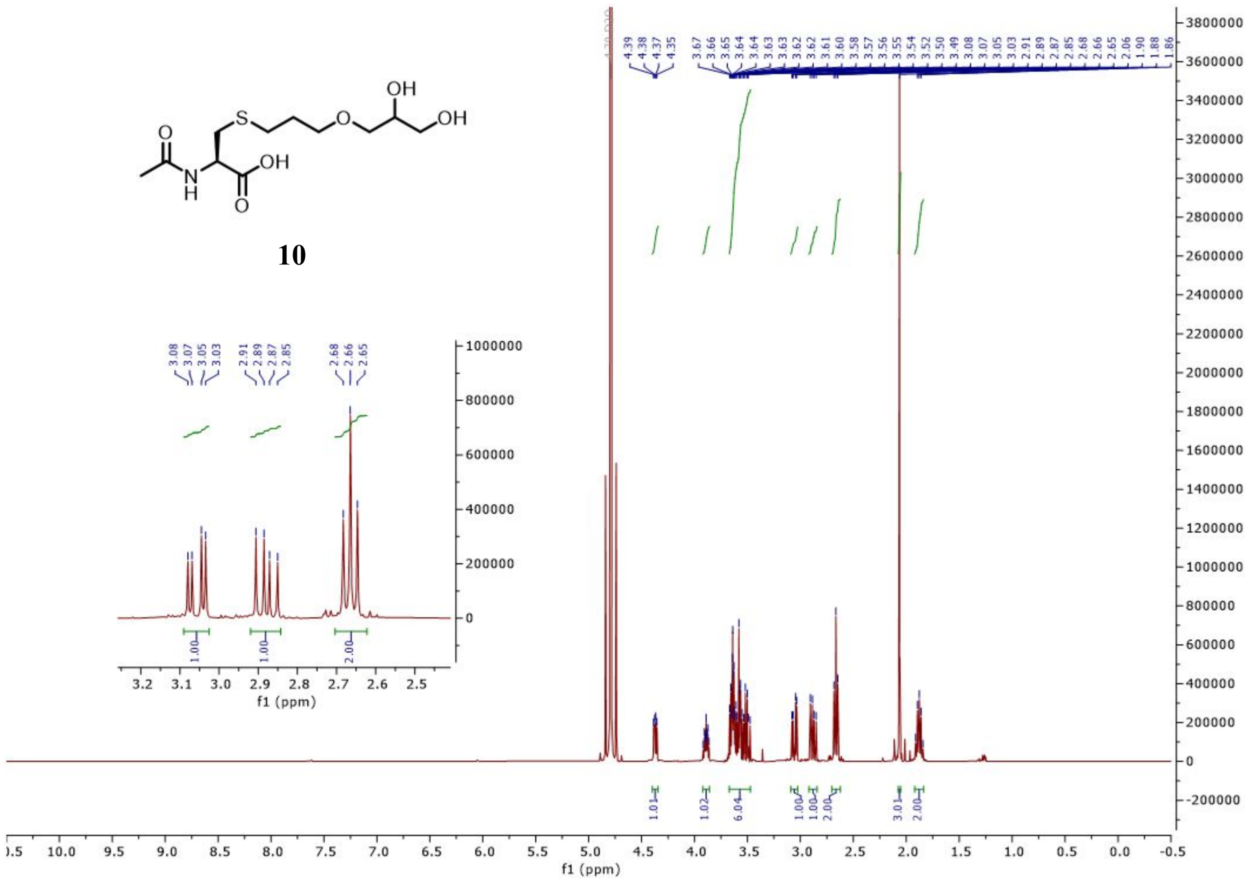

^{13}C-{^{1}H} NMR spectrum (101 MHz, D_2O) of **10**.

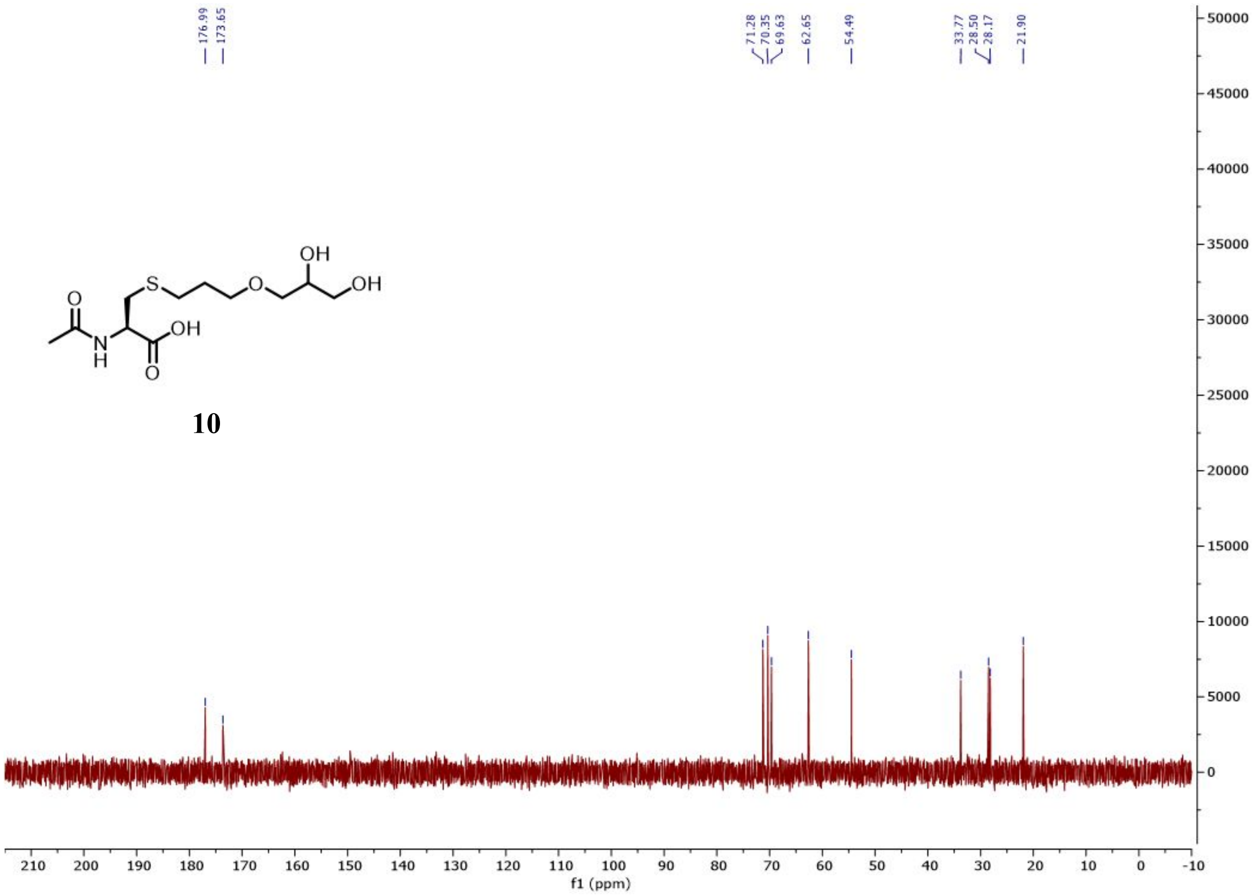

10

^{1}H NMR spectrum (400 MHz, D_2O) of **11**.

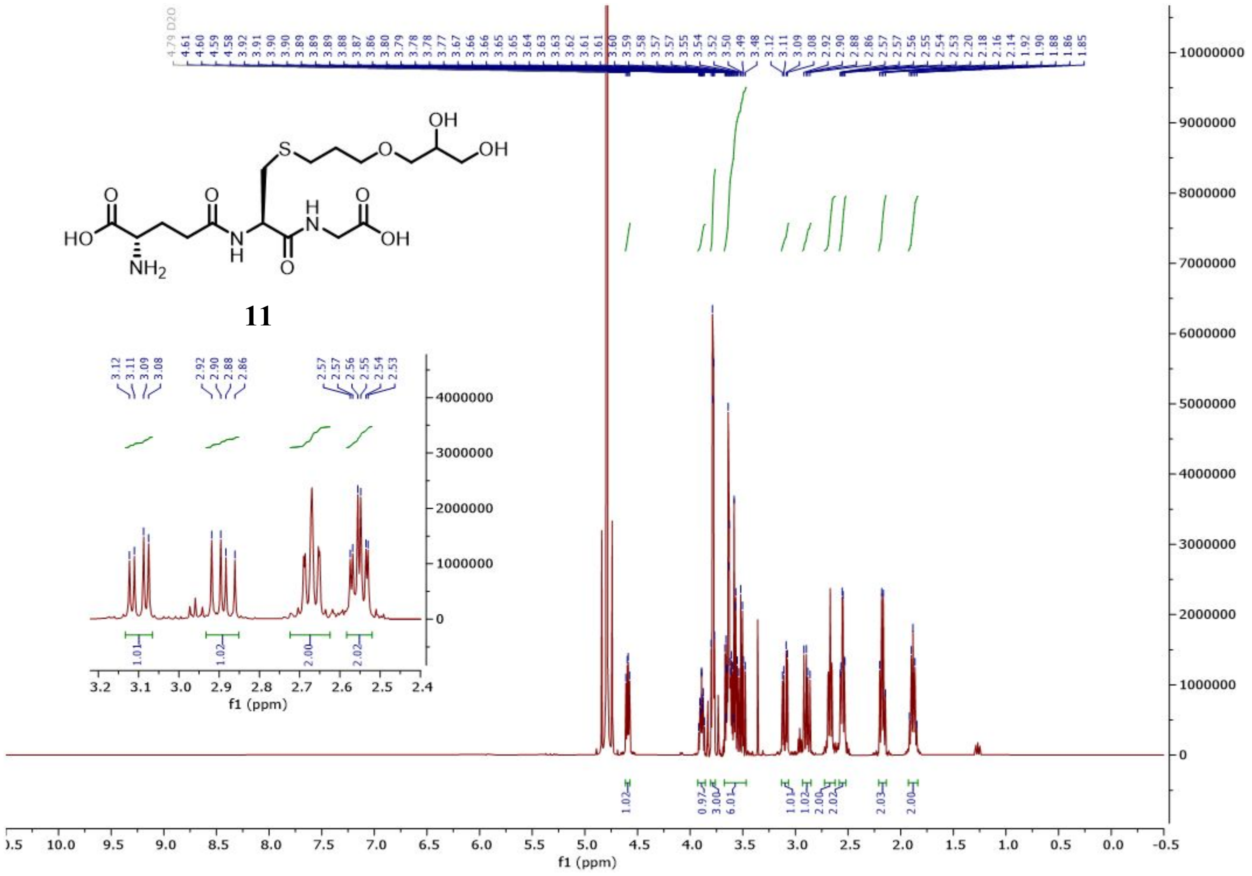

^{13}C-{^{1}H} NMR spectrum (101 MHz, D_2O) of **11**.

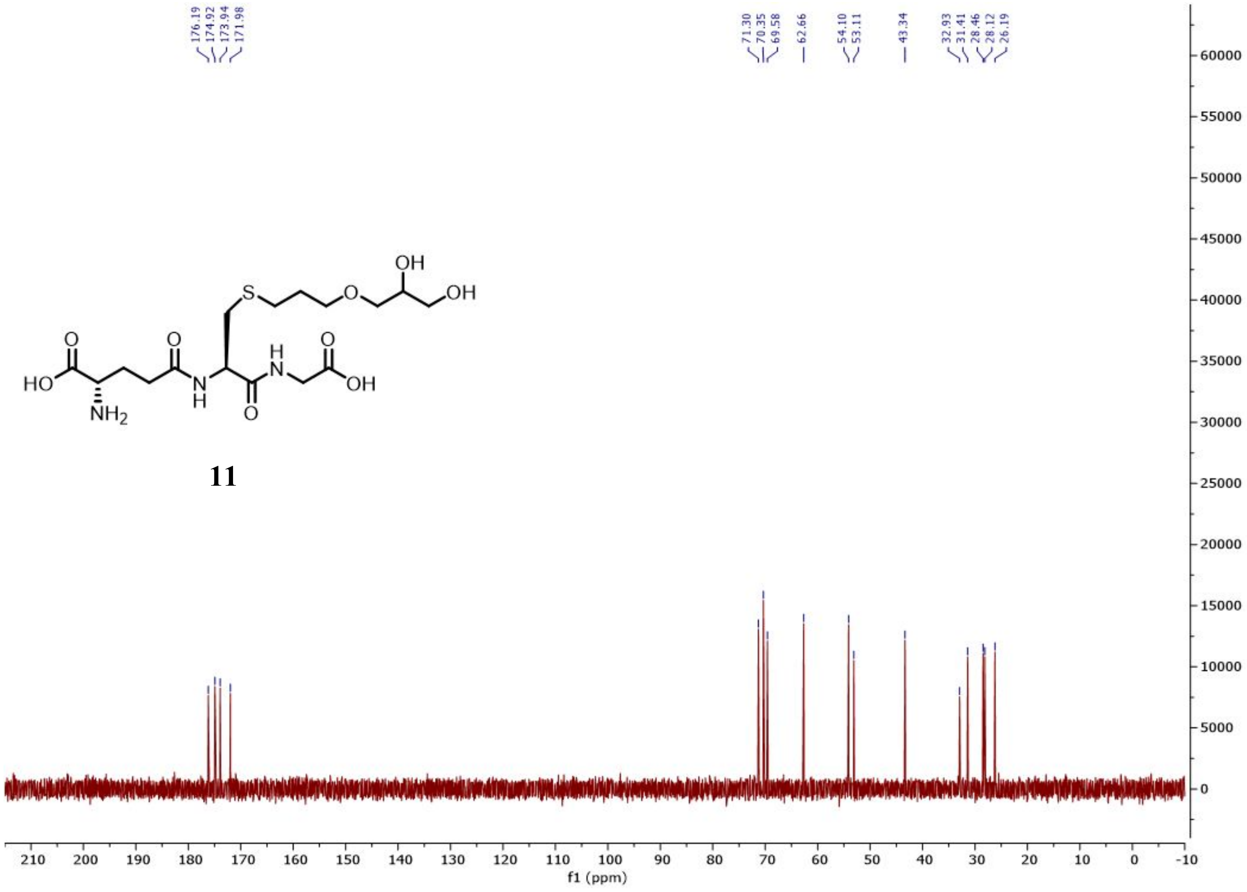

^{1}H NMR spectrum (400 MHz, D_2O) of **12**.

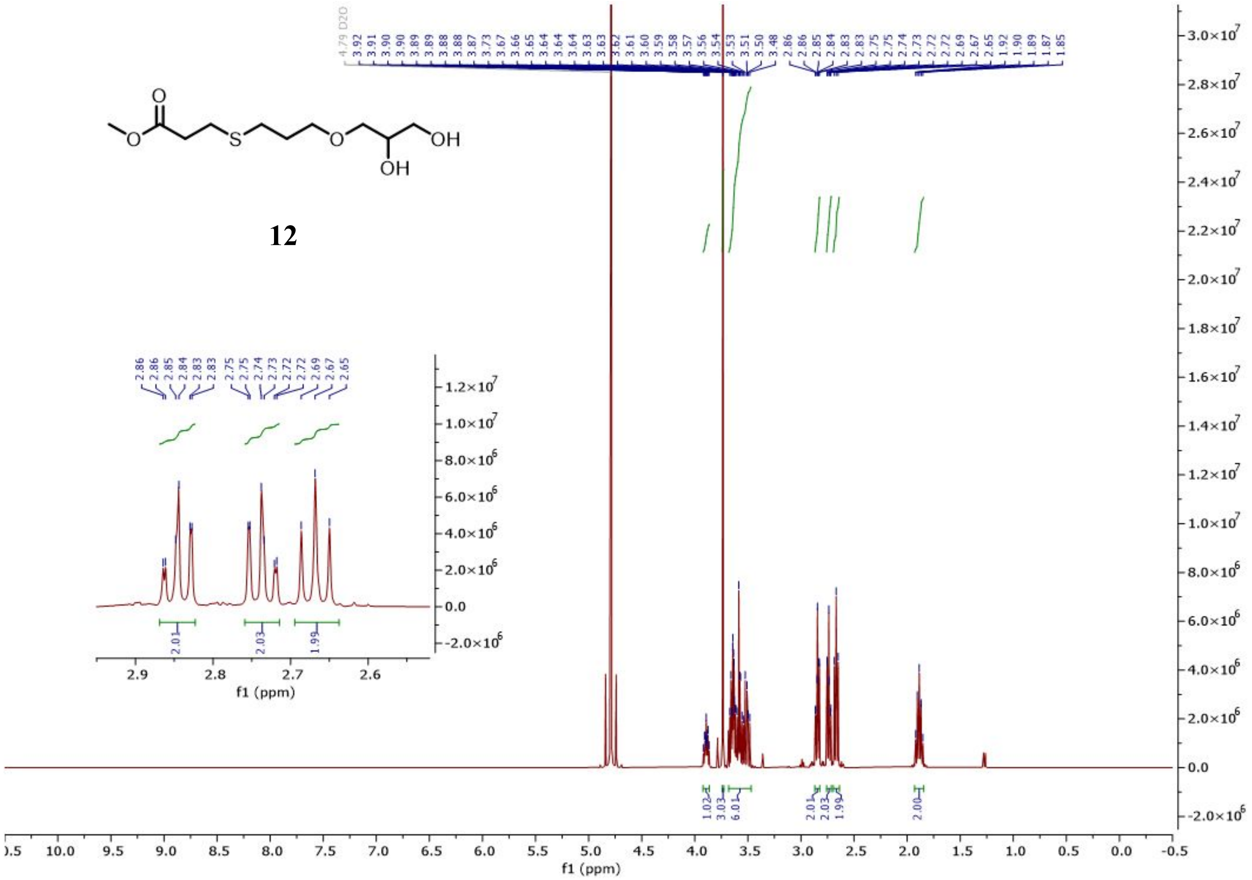

12

^{13}C-{^{1}H} NMR spectrum (101 MHz, D_2O) of **12**.

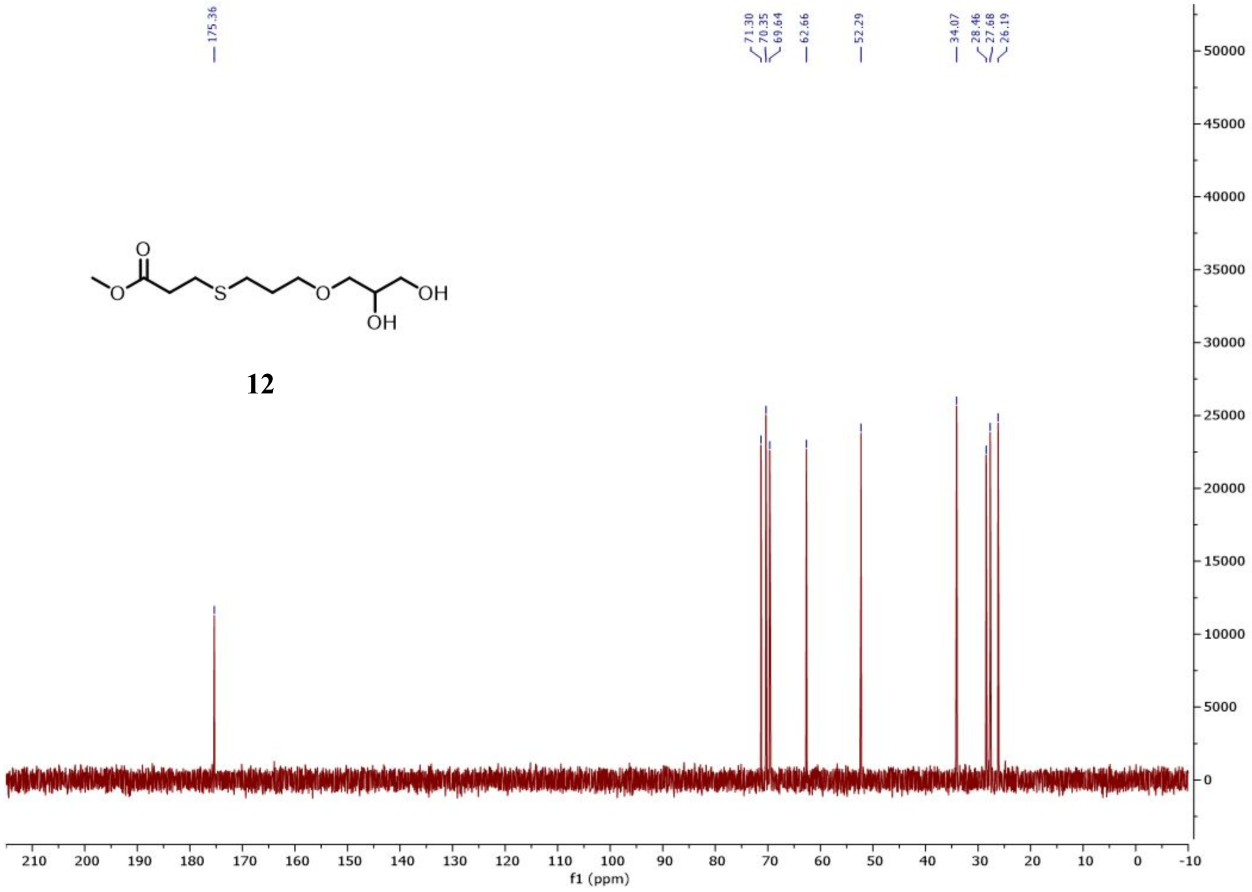

^{1}H NMR spectrum (400 MHz, D_2O) of **13**.

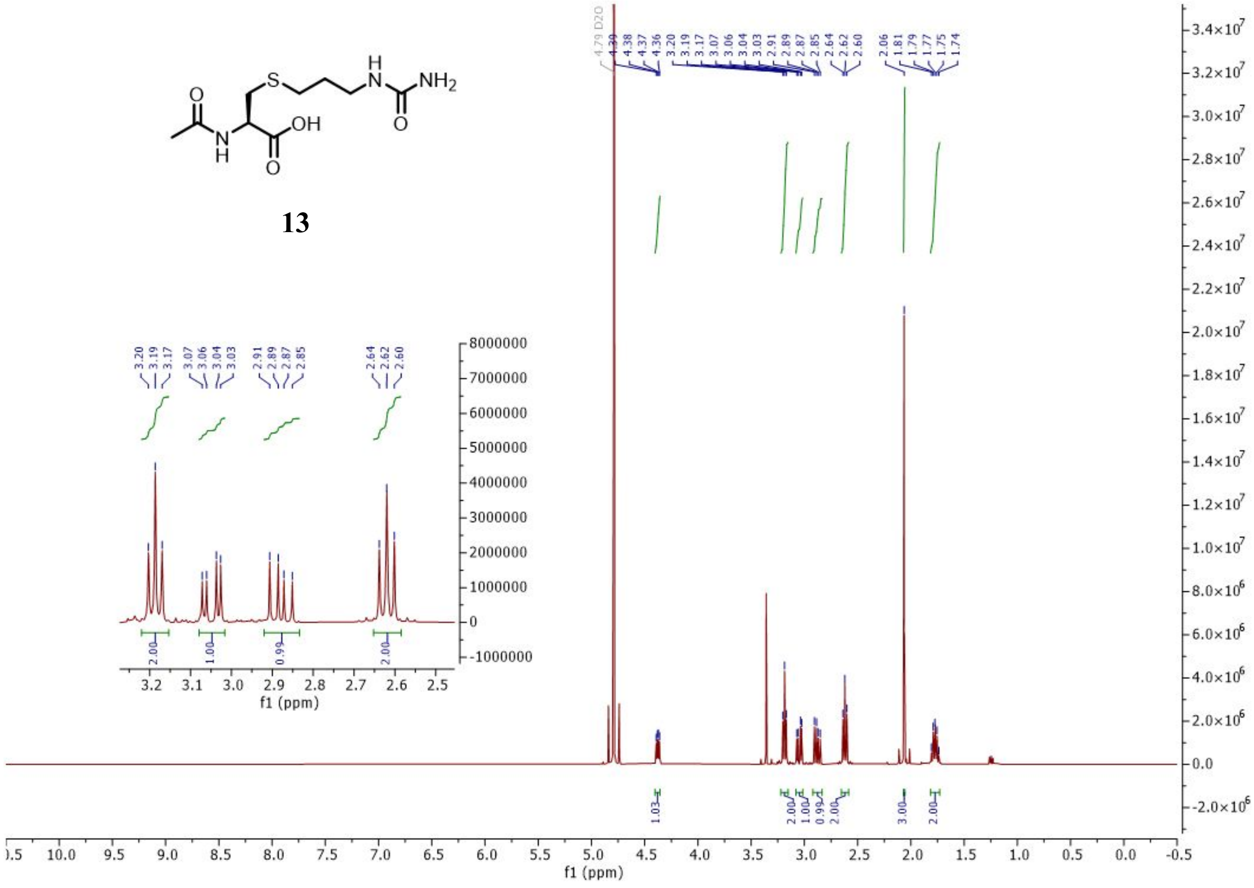

^{13}C-{^{1}H} NMR spectrum (101 MHz, D_2O) of **13**.

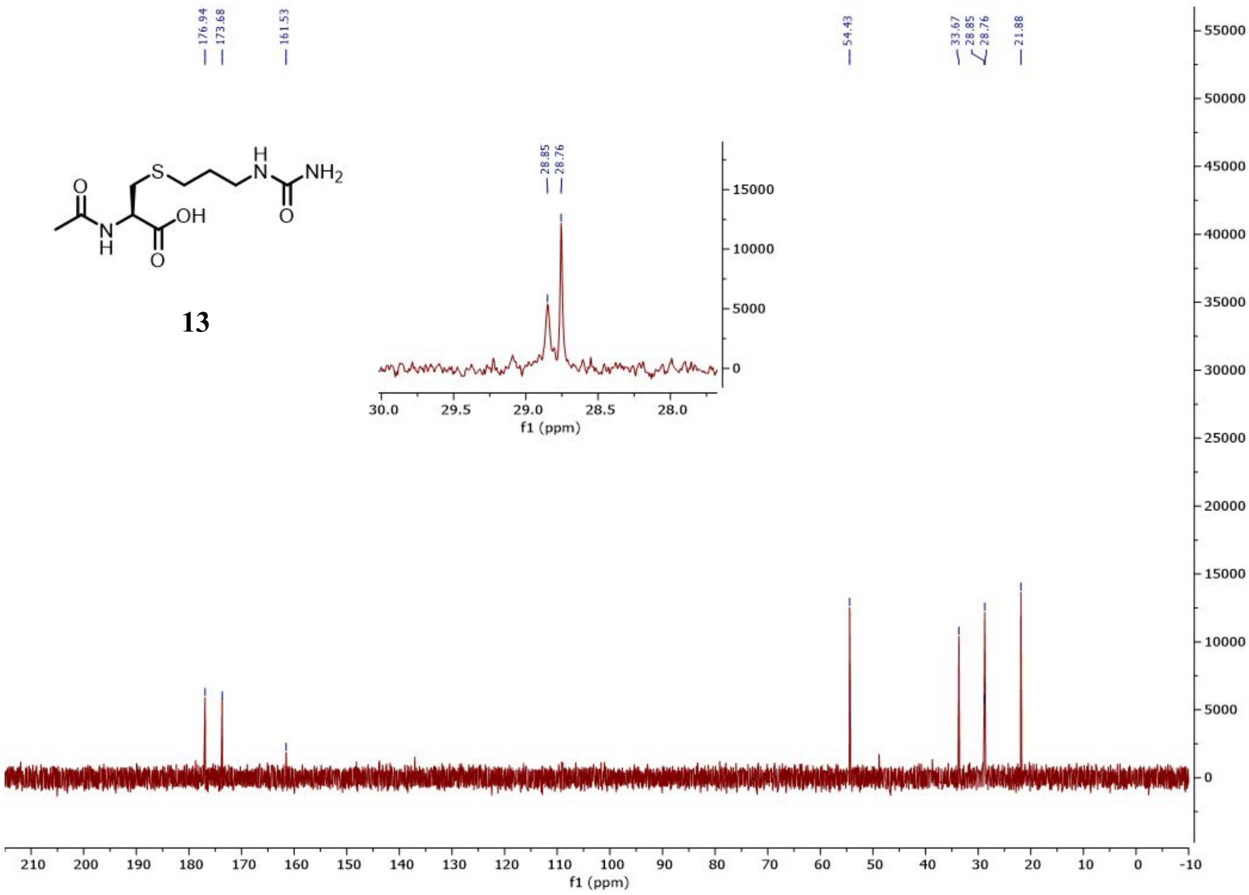

^1H NMR spectrum (400 MHz, CD_2Cl_2) of 1-(benzylamino)-2-methyl-1-oxopropan-2-yl dodecyl carbonotrithioate, CTA-benzylamine conjugate

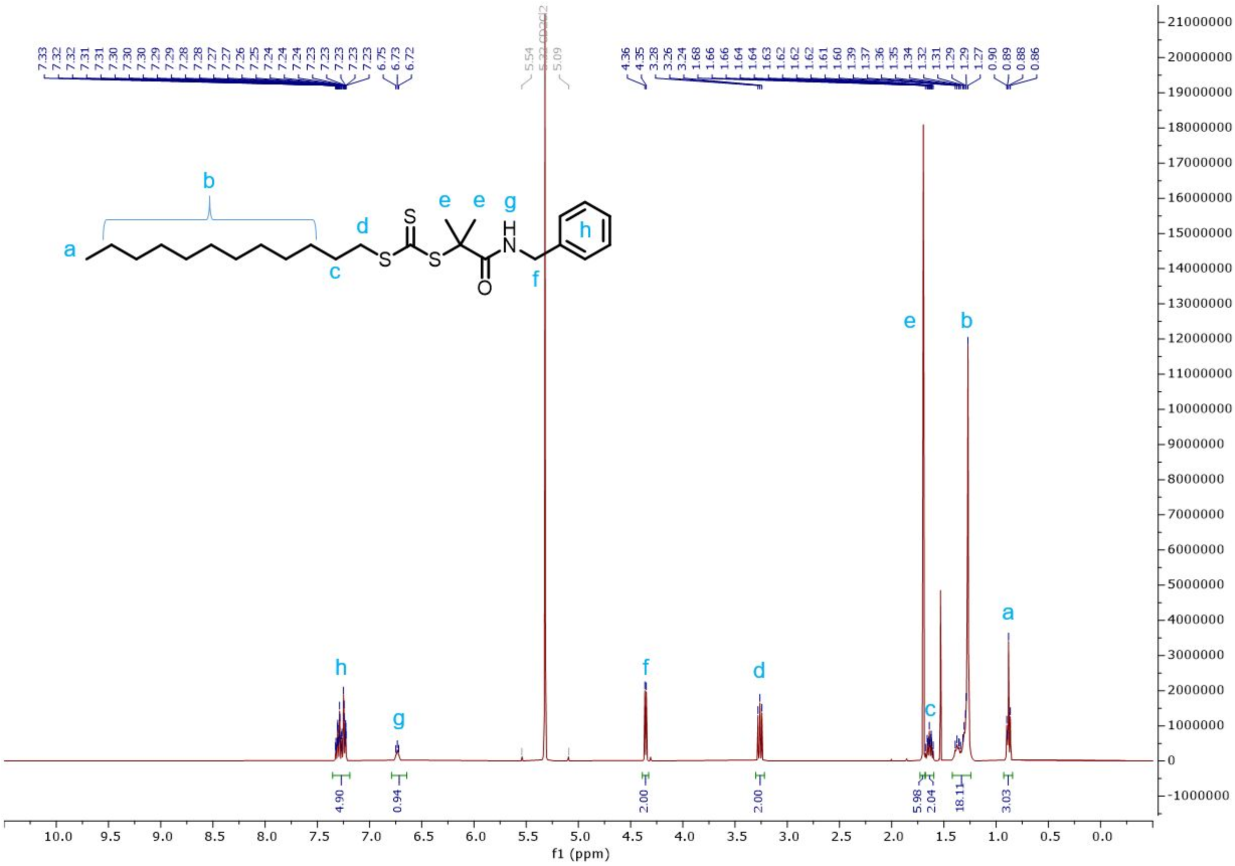

^1H NMR (400 MHz, CD_2Cl_2): δ [ppm] 7.35 – 7.19 (m, 5H), 6.73 (t, J = 5.6 Hz, 1H), 4.36 (d, J = 5.7 Hz, 2H), 3.30 – 3.22 (m, 2H), 1.70 (s, 6H), 1.68 – 1.60 (m, 2H), 1.27 (m, 18H), 0.93 – 0.84 (m, 3H).

^{13}C-{^{1}H} NMR spectrum (101 MHz, CD_2Cl_2) of CTA-benzylamine conjugate.

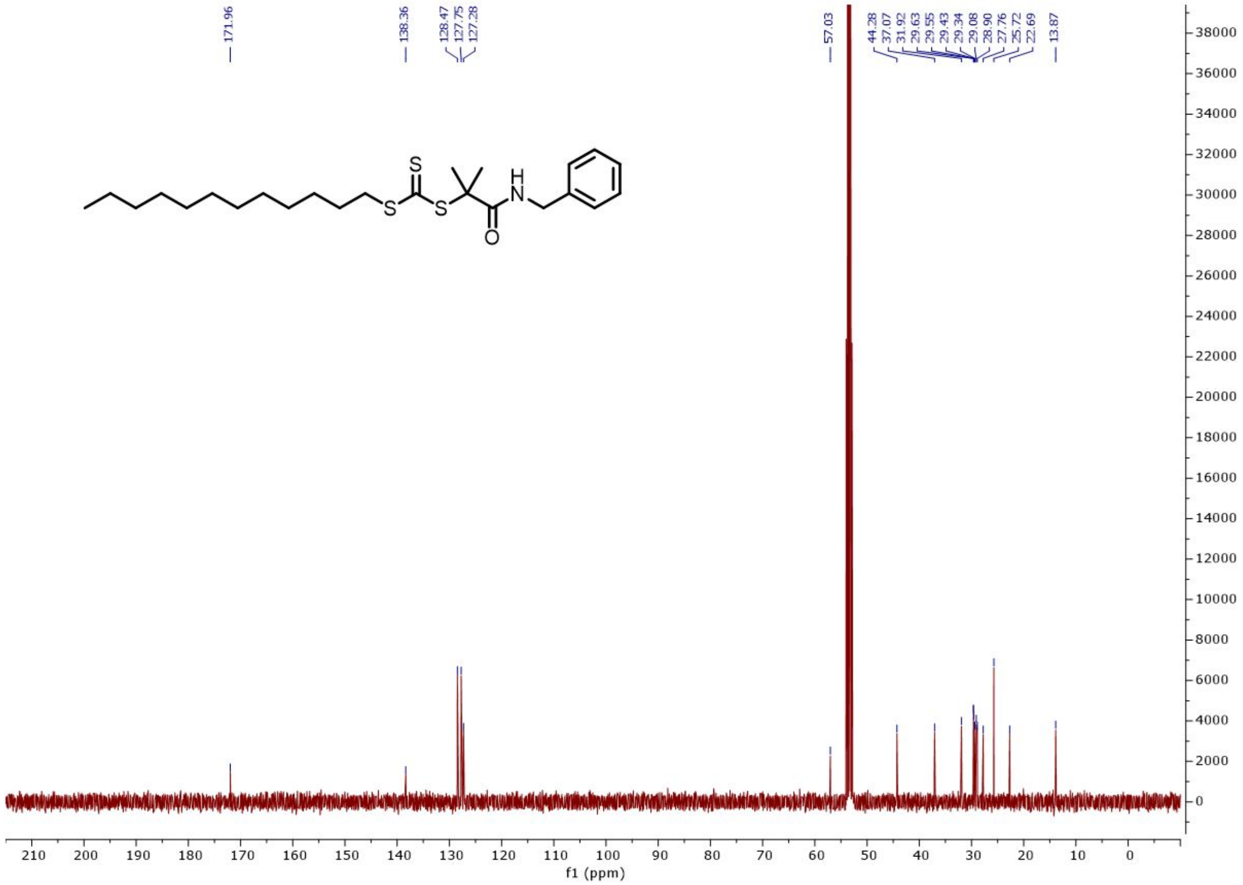

^{13}C NMR (101 MHz, CD_2Cl_2): δ [ppm] 171.96, 138.36, 128.47, 127.75, 127.28, 57.03, 44.28, 37.07, 31.92, 29.63, 29.55, 29.43, 29.34, 29.08, 28.90, 27.76, 25.72, 22.69, 13.87.
